# Genome evolution and transcriptome plasticity is associated with adaptation to monocot and dicot plants in *Colletotrichum* fungi

**DOI:** 10.1093/gigascience/giae036

**Published:** 2024-06-28

**Authors:** Riccardo Baroncelli, José F Cobo-Díaz, Tiziano Benocci, Mao Peng, Evy Battaglia, Sajeet Haridas, William Andreopoulos, Kurt LaButti, Jasmyn Pangilinan, Anna Lipzen, Maxim Koriabine, Diane Bauer, Gaetan Le Floch, Miia R Mäkelä, Elodie Drula, Bernard Henrissat, Igor V Grigoriev, Jo Anne Crouch, Ronald P de Vries, Serenella A Sukno, Michael R Thon

**Affiliations:** Department of Agricultural and Food Sciences (DISTAL), University of Bologna, Viale Fanin 40-50, 40127 Bologna, Italy; Department of Microbiology and Genetics, Institute for Agribiotechnology Research (CIALE), University of Salamanca, Calle del Duero, 37185 Villamayor, Salamanca, Spain; Department of Food Hygiene and Technology and Institute of Food Science and Technology, University of León, Campus Vegazana, 24007 León, Spain; Center for Health and Bioresources, Austrian Institute of Technology (AIT), Konrad-Lorenz-Straße 24, 3430 Tulln an der Donau, Austria; Westerdijk Fungal Biodiversity Institute & Fungal Molecular Physiology, Fungal Physiology, Utrecht University, Uppsalalaan 8, 3584 CT Utrecht, The Netherlands; Westerdijk Fungal Biodiversity Institute & Fungal Molecular Physiology, Fungal Physiology, Utrecht University, Uppsalalaan 8, 3584 CT Utrecht, The Netherlands; Joint Genome Institute, Lawrence Berkeley National Laboratory, United States Department of Energy, McMillan rd, CA 94720 Berkeley, USA; Joint Genome Institute, Lawrence Berkeley National Laboratory, United States Department of Energy, McMillan rd, CA 94720 Berkeley, USA; Joint Genome Institute, Lawrence Berkeley National Laboratory, United States Department of Energy, McMillan rd, CA 94720 Berkeley, USA; Joint Genome Institute, Lawrence Berkeley National Laboratory, United States Department of Energy, McMillan rd, CA 94720 Berkeley, USA; Joint Genome Institute, Lawrence Berkeley National Laboratory, United States Department of Energy, McMillan rd, CA 94720 Berkeley, USA; Joint Genome Institute, Lawrence Berkeley National Laboratory, United States Department of Energy, McMillan rd, CA 94720 Berkeley, USA; Joint Genome Institute, Lawrence Berkeley National Laboratory, United States Department of Energy, McMillan rd, CA 94720 Berkeley, USA; Laboratory of Biodiversity and Microbial Ecology (LUBEM), IBSAM, ESIAB, EA 3882, University of Brest, Technopôle Brest-Iroise, Parv. Blaise Pascal, 29280 Plouzané, France; Department of Microbiology, Faculty of Agriculture and Forestry, University of Helsinki, Siltavuorenpenger 5, 00170 Helsinki, Finland; UMR 7257, Architecture et Fonction des Macromolécules Biologiques, The French National Centre for Scientific Research (CNRS), University of Aix-Marseille (AMU), 163 Avenue de Luminy, Parc Scientifique et Technologique de Luminy, 13288 Marseille, France; The French National Institute for Agricultural Research (INRA), USC 1408 AFMB, 163 Avenue de Luminy, Parc Scientifique et Technologique de Luminy, 13288 Marseille, France; UMR 7257, Architecture et Fonction des Macromolécules Biologiques, The French National Centre for Scientific Research (CNRS), University of Aix-Marseille (AMU), 163 Avenue de Luminy, Parc Scientifique et Technologique de Luminy, 13288 Marseille, France; The French National Institute for Agricultural Research (INRA), USC 1408 AFMB, 163 Avenue de Luminy, Parc Scientifique et Technologique de Luminy, 13288 Marseille, France; Department of Biological Sciences, King Abdulaziz University, 23453 Jeddah, Saudi Arabia; Joint Genome Institute, Lawrence Berkeley National Laboratory, United States Department of Energy, McMillan rd, CA 94720 Berkeley, USA; Department of Plant and Microbial Biology, University of California Berkeley, Berkeley, CA, USA; Mycology and Nematology Genetic Diversity and Biology Laboratory, Agricultural Research Service, United States Department of Agriculture, 10300 Baltimore Ave, MD 20705, Beltsville, USA; Westerdijk Fungal Biodiversity Institute & Fungal Molecular Physiology, Fungal Physiology, Utrecht University, Uppsalalaan 8, 3584 CT Utrecht, The Netherlands; Department of Microbiology and Genetics, Institute for Agribiotechnology Research (CIALE), University of Salamanca, Calle del Duero, 37185 Villamayor, Salamanca, Spain; Department of Microbiology and Genetics, Institute for Agribiotechnology Research (CIALE), University of Salamanca, Calle del Duero, 37185 Villamayor, Salamanca, Spain

**Keywords:** fungal genomics, comparative transcriptomics, fungal evolution, anthracnose, plant cell walls

## Abstract

**Background:**

*Colletotrichum* fungi infect a wide diversity of monocot and dicot hosts, causing diseases on almost all economically important plants worldwide. *Colletotrichum* is also a suitable model for studying gene family evolution on a fine scale to uncover events in the genome associated with biological changes.

**Results:**

Here we present the genome sequences of 30 *Colletotrichum* species covering the diversity within the genus. Evolutionary analyses revealed that the *Colletotrichum* ancestor diverged in the late Cretaceous in parallel with the diversification of flowering plants. We provide evidence of independent host jumps from dicots to monocots during the evolution of *Colletotrichum*, coinciding with a progressive shrinking of the plant cell wall degradative arsenal and expansions in lineage-specific gene families. Comparative transcriptomics of 4 species adapted to different hosts revealed similarity in gene content but high diversity in the modulation of their transcription profiles on different plant substrates. Combining genomics and transcriptomics, we identified a set of core genes such as specific transcription factors, putatively involved in plant cell wall degradation.

**Conclusions:**

These results indicate that the ancestral *Colletotrichum* were associated with dicot plants and certain branches progressively adapted to different monocot hosts, reshaping the gene content and its regulation.

## Introduction

The plant cell wall (PCW) consists of many different interconnected polysaccharides, providing strength and structure. In addition, PCWs are determinants of immune responses since modification of their composition affects disease resistance and fitness in plants [1–3].

The PCW can be seen as one of the first layers of defense where the arms race between the pathogen and the host takes place but also as a complex ecological niche where the fungi (pathogenic as well as mutualistic) retrieve most of the nutrients from the host during the interaction. To release the monomers present in these complex plant structures, fungi need to simultaneously secrete several plant biomass degrading enzymes, mainly associated with hydrolytic and oxidative functions [2]. Plants protect themselves against degradation of their cell walls by producing proteins that inhibit microbial cell wall degrading enzymes (CWDEs); for example, inhibitors of pectin degrading enzymes are common in dicots and noncommelinoid monocots, and inhibitors of xylan degrading enzymes are common in the Poaceae [4]. The production of these inhibitors by plants has, in turn, driven the evolution of some CWDE groups of phytopathogenic fungi toward inhibitor-resistant enzymes [5]. In some phytopathogenic fungi, there is evidence for production of different amounts of specific CWDEs, depending on whether the plant host is a monocot or dicot [6–8].

*Colletotrichum* is a genus of plant pathogenic fungi that are known for their wide host range and diversity of pathogenic and nonpathogenic lifestyles. They are responsible for a large number of diseases, collectively known as anthracnose, which can cause significant damage on a wide range of economically important plants [9]. In addition to their economic importance, *Colletotrichum* spp. have been extensively utilized as model species to investigate plant–fungus interactions. For all these reasons, *Colletotrichum* has been ranked among the top 10 most important fungal plant pathogens worldwide [10]. Some *Colletotrichum* species show a one-to-one relationship with a specific host while other species infect a wide range of hosts [6, 9, 11–13]. The biological diversity of *Colletotrichum* and the presence of very closely related species with different host ranges makes this genus an excellent model to investigate genomic signatures associated with the evolution of biological characters important for host interactions such as those involved in PCW degradation.

Since the first genome sequences of fungi became available, researchers have been analyzing gene content and genomic features to find associations that may explain the differences in fungal lifestyles, and varying patterns are beginning to emerge [6, 14, 15]. In contrast, gene loss or gain in families such as those encoding CAZymes and proteases could be associated with host range in *Colletotrichum* species [16]. The similar repertoires of CAZymes and secreted proteases found in relatively distant members of the *Colletotrichum acutatum* and *Colletotrichum gloeosporioides* species complexes suggest a recent and independent acquisition of this enzymatic arsenal or a progressive loss during the host specialization process [6, 16, 17]. While genome studies are useful tools to identify putative genes and to perform evolutionary analyses, transcriptomic data are required to better understand the genes involved in a complex process such as PCW interaction.

Plant pathogenic fungi have a close interaction with the PCW, and plants have evolved to recognize external attacks through the degradation of the PCW itself. This is especially true for hemibiotrophic plant pathogens as they interact with the PCW twice: initially when they enter the cell and later when they gain nutrients from it. This complexity is reflected by the wide arsenal of CAZymes encoded by *Colletotrichum* spp. being one of the most diverse in the fungal kingdom.

In this work, we used comparative genomics and transcriptomics to identify genes involved in the interaction between *Colletotrichum* spp. and the plant substrates (which are mainly composed by PCW), as well as evolutionary analyses to gain a better understanding of adaptation and specialization of these fungi to different plant substrates. Phylogenetic analyses revealed that the ancestral *Colletotrichum* was associated with dicots and that at least 3 independent jumps to monocots occurred. We also found that monocot-associated *Colletotrichum* species have undergone specific gene losses in PCW degrading enzyme families and expansions in lineage-specific genes. Comparing 4 different *Colletotrichum* species, we also found that, despite millions of years of divergent evolution, they have maintained highly similar gene content, with exceptions in the CAZymes and proteases, and show strong differences in gene modulation associated with different host substrates.

## Results

### The common ancestor of *Colletotrichum* parasitized dicots and specific lineages jumped independently to monocots

In this study, we present a comparative genomic analysis of 30 species from the genus *Colletotrichum*. Eleven of these (*Colletotrichum cereale, Colletotrichum eremochloae, Colletotrichum sublineola, Colletotrichum graminicola, Colletotrichum falcatum, Colletotrichum navitas, Colletotrichum caudatum, Colletotrichum somersetensis, Colletotrichum zoysiae, Colletotrichum orchidophilum*, and *Colletotrichum phormii*) are pathogens specialized to different taxonomic groups of monocots; seventeen (*Colletotrichum orbiculare, Colletotrichum noveboracense, Colletotrichum higginsianum, Colletotrichum tofieldiae, Colletotrichum salicis, Colletotrichum godetiae, Colletotrichum acutatum sensu stricto, Colletotrichum fioriniae, Colletotrichum abscissum, Colletotrichum lupini, Colletotrichum tamarilloi, Colletotrichum costaricense, Colletotrichum cuscutae, Colletotrichum paranaense, Colletotrichum melonis, Colletotrichum nymphaeae*, and *Colletotrichum simmondsii*) have been associated only with dicots while two of them (*Colletotrichum chlorophyti* and *Colletotrichum incanum*) are capable of infecting plants that belong to both groups.

**Figure 1:**
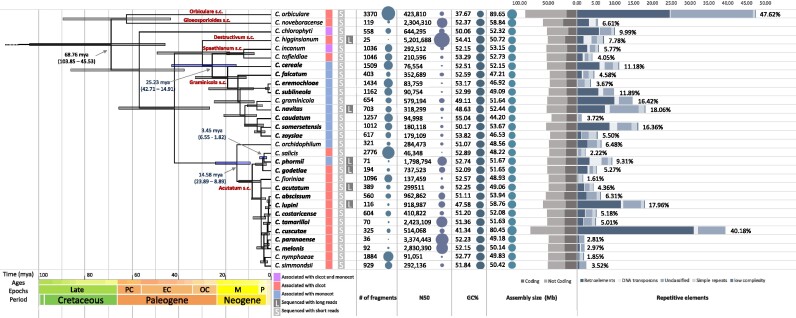
A timetree inferred by the RelTime method to the *Colletotrichum* phylogenomic tree. The branch lengths were calculated using the ordinary least squares method. All nodes are supported by Bayesian posterior probability of 1.00. Bars around each node represent 95% confidence intervals and light blue bars represent the 3 host jumps from dicot to monocot. This analysis involved 127 amino acid sequences and a total of 124,023 sites. *Colletotrichum* species complexes are indicated in red. Genomes sequenced in the present study are highlighted in bold. On the right side, 4 bubble plots illustrating assembly size, GC content, and assembly fragmentation parameters (number of contigs and N50 value) are reported on the right side. The bubble sizes have been scaled to each panel and are not comparable across panels. Gray bar diagram on the right reports the size of coding and noncoding regions, while the blue one represents the percentage of repetitive elements in each genome (Supplementary Table S2).

**Figure 2:**
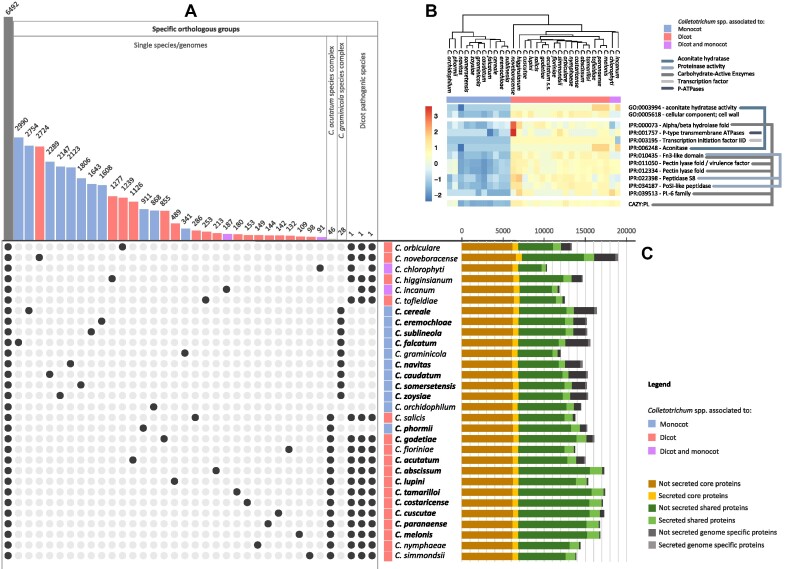
Comparative genomic analysis of *Colletotrichum* species. (A) UpsetR plot of the protein clustering analysis. Bars on the upper side represent the number of orthogroups shared by the species highlighted by the black dots reported on the bottom side. (B) Hierarchical clustering of disjoint sets of terms and gene families identified in *Colletotrichum* species associated with monocots and dicots hosts. Gene Ontology and InterPro terms corresponding to the rows are reported on the right; colored lines connect overlapping terms (Supplementary Tables S3, S4, S5, S6, S7, S8, and S9). Hierarchical clustering of genes and species was performed and visualized using the UPGMA algorithm, including overrepresented (orange to red) and underrepresented functional domains (blue). (C) Bar diagrams show the number of proteins shared with all included species (in yellow), shared with at least 2 but not all (in green), and those found in only 1 species (in gray). The light shading indicates for each group the portion of proteins predicted to be secreted.

All genomes have been analyzed for completeness to avoid a potential source of bias (Supplementary Table S1). The analyzed genomes showed a large variation in size, ranging from 44.20 Mb in *C. caudatum* to 89.65 Mb in *C. orbiculare* (Fig. 1). While a large variation at the genus level was already reported [18] (more than 50% in our dataset), these results highlight an unexpected variation of more than 30 Mb (39%) between 2 closely related species such as *C. cuscutae* and *C. paranaense*. These 2 species belong to the Acutatum species complex and have been recognized as separate taxa only recently. As a general trend, species with larger genomes have approximately the same number of genes as smaller genomes and are characterized by a lower GC content (Figs. 1 and 2). Identification and characterization of repetitive elements reveal a high diversity in repeat content among different *Colletotrichum* species and demonstrate the proliferation of retroelements and other unclassified repeats in the genomes characterized by larger genome sizes.

Phylogenomic analyses calibrated with 3 fungal fossils show age estimates for *Colletotrichum* spp. and enable the identification of time frames of specific evolutionary events (Fig. 1).

*Colletotrichum* species diverged from members of the closest related genus *Verticillium* in the late Jurassic around 136.43 million years ago (mya) (186.35–99.88) (Supplementary Fig. S1). The diversification of species within the genus, based on the estimation of divergence between the 2 most distantly related species *C. orbiculare* and *C. abscissum*, took place during the Upper (or Late) Cretaceous period, 68.76 mya (103.85–45.53). These results suggest that the common ancestor of *Colletotrichum* was associated with dicots, and at least 3 independent host jumps between dicots and monocots took place during the evolution of this pathogen. The first took place in the Paleogene (around 25 mya) when species of the Graminicola complex diverged from those belonging to the Spaethianum complex. Interestingly, the diversification of *Colletotrichum* species, adapted to plant species belonging to the Poaceae, happened around 20 mya, coinciding with the expansions of grasses from their water-bank habitat into open tracts and their diversification [19]. The second happened around 15 mya when *C. orchidophilum* diverged from the ancestor of the Acutaum species complex. The third host jump occurred in the Neogene around 3.5 mya when the flax pathogenic species *C. phormi* diverged from its closest related species, *C. salicis*.

### *Colletotrichum* species associated with monocots have gone through expansions of lineage-specific genes and losses of degradative enzymes and other conserved functions

To examine core features shared by all *Colletotrichum* species, complexes, individual species, and features specific to dicot- and monocot-associated species, all predicted proteomes were clustered into groups of orthologous genes (Fig. 2A). This approach enabled the identification of the core, shared, and species-specific proteins and orthologs only present in species associated with dicot or monocot hosts. Enrichment analyses of the core, shared, and lineage-specific (secreted and nonsecreted) protein encoding genes did not identify functional category or gene family expansions associated with host range. Considering that the analyses carried out are affected by the sampling, as closely related species are likely to have more shared genes compared to species that are more distant from others, our analyses also highlight that monocot pathogenic species have generally more lineage-specific genes compared to dicot pathogenic species (Fig. 2A, C). The lineage-specific genes of 2 closely related pairs of species were compared to their counterpart’s genome (Supplementary Fig. S2). Interestingly, most of the lineage-specific genes have homology to the closely related genome, but manual inspection of the sequence alignments revealed that most have deletions and/or nucleotide substitutions, suggesting that the lineage-specific genes are the result of gene loss in the other species. While no orthogroups specific to the monocot pathogenic species were identified, we found 3 orthogroups only present in those species capable of infecting dicot plants. These were OG0010350, with 1 or 2 copies of the gene present in all dicot pathogenic species and in *C. incanum* and characterized as a secreted β-glucosidase (CAZy—GH3/FN3); OG0010637, with 1 or 2 copies of the gene present in all dicot pathogenic species and in *C. incanum* and characterized as a secreted protein with unknown function containing a (FAD)-binding domain; and OG0011101, present in all dicot pathogenic species and in those that have been associated with dicot and monocot and described as an α-1,2-mannosidase (CAZy—GH92).

Analyses of functional annotations highlighted 2 Gene Ontology (GO), 12 InterPro (IPR) terms and 2 gene families expanded in dicot-associated species compared to the monocot-associated species (Fig. 2B; Supplementary Tables S3, S4, and S10). No terms were expanded in monocot-associated *Colletotrichum* spp., confirming the pattern observed in the analyses based on protein similarity, and the 2 species capable of infecting both hosts (*C. chlorophyti* and *C. incanum*) cluster with the dicot-associated pathogens. As many IPR and GO terms overlap, the results were manually inspected to avoid redundancy.

Overall, terms identified as expanded in dicot-associated pathogens could be clustered into 5 functional groups (Fig. 2B): (i) aconitases are genes encoding for enzymes that catalyze the stereo-specific isomerization of citrate to isocitrate in the Krebs cycle, and while dicot pathogens have 3 or 4 copies of this gene, monocot pathogens have only 2; (ii) P-ATPases are proteins that are involved in transport of a variety of different compounds; (iii) transcription initiation factor IID is a general transcription factor (GTF) involved in accurate initiation of transcription by RNA polymerase II; (iv) serine proteases belonging to the MEROPS peptidase family S8; and (v) several terms identified, such as the alpha/beta hydrolase fold, the pectin lyase fold, the PL6 family domains, and others are associated with CAZymes.

Dicot-infecting species have a higher overall number of genes encoding putative plant biomass degrading enzymes than the species with monocot hosts (Supplementary Table S10), which confirms previous studies [6]. This is also clear by the number of CAZy families encoding carbohydrate esterases (CEs), glycoside hydrolases (GHs), or polysaccharide lyases (PLs), for which the dicot-infecting species have a significantly higher number of genes. In contrast, higher gene numbers per family for the monocot-infecting species are only present in CE1, GH10, GH11, GH13_1, GH45, and GH62. Interestingly CE1, GH10, GH11, and GH62 are all involved in xylan degradation, a prominent component of monocot cell walls. CAZy families encoding putative pectinolytic enzymes have higher numbers of genes in the dicot-infecting species, such as CE8, CE12, GH28, GH43, GH52, GH53, GH78, GH88, GH93, PL1, PL3, PL11, and PL26. However, also CAZy families with putative enzymes targeting lignin (AA1), cellulose (GH1, GH3, GH5, GH7), and hemicellulose (CE16, GH12, GH27, GH36, GH74, GH115) are enriched in the dicot-infecting species. At the individual species level, *C. noveboracense* stands out with an increased number of genes in several CAZy families (AA1_3, CE1, GH1, GH2, GH7, GH28, GH43, GH78). The *Colletotrichum* species lack the subfamily AA1_1 *sensu stricto* laccases but possess putative laccase-like multicopper oxidase encoding genes from the subfamilies AA1_2 and AA1_3. A previously described laccase (*lac2*), which is involved in melanization in appressorial cells of *C. orbiculare* [20], is categorized as a member of family AA1 without a subfamily division, whereas a *C. orbiculare lac1* that does not have a role in melanin biosynthesis or pathogenicity [20] is cataloged to AA1_3. For 3 of the species, *C. acutatum, C. higginsianum*, and *C. graminicola*, growth profiles on plant biomass–related substrates are available in the FUNG-GROWTH database [21]. Comparison of the CAZome of these 3 species (Supplementary Tables S9 and S10) to their growth profiles did not provide clear correlations. Growth on xylan, galactomannan (guar gum), and inulin is relatively poor for *C. higginsianum* compared to the other 2 species, but no strong reduction in xylanolytic, mannanolytic, or inulinolytic genes can be found in its genome. This evidence also suggests that the CAZyme content in the genome can only partially explain its degradative capability.

To confirm these results and to gain a better understanding on the evolution of the genes identified using both approaches (similarity-based protein clustering and protein terms enrichment), further analyses were carried out. Results of selected CAZy families (GH3 and GH92), aconitases, and transcription initiation factors IID (Supplementary Fig. S3) revealed gene losses in the monocot-associated species lineages.

### Transcriptome profiles on different plant substrates reveal strong variation among species

To identify genes involved in the interaction with the PCW, we performed a transcriptome analysis of 4 reference species, with 2 dicot pathogens (*C. higginsianum, C. nymphaeae*) and 2 monocot pathogens (*C. graminicola* and *C. phormii*), on 3 different substrates: D-glucose, sugar beet pulp (dicot substrate: DS), and maize powder (derived from complete plants without cobs as monocot substrate: MS). Species used in the transcriptomic approach have been selected because they represent model systems (e.g., *C. graminicola* and *C. higginsianum*) and are based on differences in evolutionary history of host association (species that have a long history of host association with monocots like *C. graminicola* and species that have adapted to monocots more recently like *C. phormi*).

The selected substrates differ in sugar/polysaccharide composition, with sugar beet pulp being rich in cellulose, pectin, and xyloglucan [22], while maize powder is rich in cellulose and hemicellulose, particularly glucuronoarabinoxylan [23]. Both plant substrates have been used as valuable waste biomass for industrial applications [24–26] and therefore largely used as substrates in similar studies to address the microbial degradation performance/requirements [27–29].

**Figure 3:**
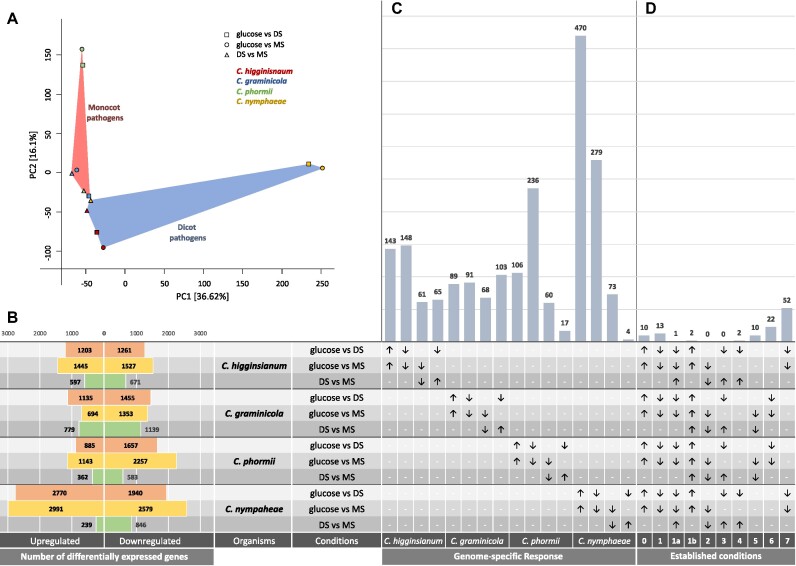
Comparative transcriptomic analysis of selected *Colletotrichum* species (*C. higginsianum, C. graminicola, C. phormii*, and *C. nymphaeae*) on 3 different carbon sources: D-glucose, sugar beet pulp (as dicot substrate: DS), and maize powder (as monocot substrate: MS). (A) Principal components analysis of all the orthogroups identified in the 4 species analyses and associated expression profiles (Supplementary Table S11). (B) Number of differentially expressed genes of each *Colletotrichum* species and in each condition analyzed. (C, D) Genome-specific response represented as the number of genes differentially expressed. For each pairwise comparison and species, overexpressed genes are indicated by an arrow pointing up while those underexpressed are indicated by an arrow pointing down. (C) Genes overexpressed in D-glucose for each genome are reported in the first column on the left, those overexpressed in PS are reported in the second column, those overexpressed in MS are reported in third column, and those overexpressed in DS are reported in forth column. (D) Numbers of genes showing the same expression patterns in the established conditions as described in the Materials and Methods section.

The 4 species show different evolutionary histories and genetic distances with *C. phormii* and *C. nymphaeae* being closely related members of the same complex but associated with monocot and dicot hosts, respectively. *C. higginsianum, C. phormii*, and *C. nymphaeae* have similar patterns of gene expression when the pairwise comparisons of transcriptome patterns are plotted in a principal component analysis (Fig. 3A). In these 3 species, the comparison of genes differentially expressed in DS versus MS shows a lower diversity compared to the one highlighted in the comparison of genes differentially expressed in both substrates versus D-glucose (Fig. 3A). This pattern is also confirmed by the overall number of differentially expressed genes (DEGs), where *C. higginsianum, C. phormii*, and *C. nymphaeae* have the lowest number of both up- and downregulated DEGs in DS versus MS while *C. graminicola* has a comparable number of DEGs in other pairwise comparisons (Fig. 3B). The differences shown by *C. graminicola* might reflect the longer evolutionary history of association with its host as well as the differences in plant substrate composition between the hosts. Among the 4 species, *C. nymphaeae* regulate differentially more genes compared to the other species.

To better understand the specificity of the response to different substrates, we identified species-specific genes overexpressed in the presence of D-glucose, DS, MS, plant substrate (PS: as those genes overexpressed in the presence of both DS and MS), and those shared among all 4 species, among the dicot pathogens and among the monocot pathogenic species (Fig. 3C, D). Results highlighted a strong specific response by the 4 species, as the majority of the DEGs are not shared between the 4 genomes but are specific for each organism.

Comparative analysis of enrichment profiles highlighted 5 terms enriched among overexpressed genes in dicot pathogens on DS (condition 7), all of which (GO:0000981, GO:0006355, IPR001138, IPR036864, PF00172) are associated with the Zn(2)-Cys(6) fungal-type DNA-binding domain and transcription regulation. Functional annotation of genes identified in Fig. 3D revealed that more than one-third of all genes identified (32/112) were assigned to 3 major groups: transporters, CAZymes, and transcription factors.

We identified 10 orthologous genes overexpressed in the presence of D-glucose compared to plant substrate (condition 0). Among these, 4 are transporters, 3 are associated with primary metabolism (such as citrate and fatty acid synthase and sorbitol dehydrogenase), 1 is a secreted flavoenzyme, and 2 are secreted proteins of unknown function. Sixteen orthologous genes in each species were upregulated in the presence of the plant substrates (conditions 1, 1A, and 1B). In this set, we identified 4 transporters; 2 transcription factors; 3 genes belonging to CAZy families GH27, GH5_16, and GH43; and 1 subclass M28 peptidase. Interestingly, 1 orthogroup (OG_12813) assigned to condition 1a and therefore to genes overexpressed in the presence of plant substrate by all 4 species, but more overexpressed in dicot pathogenic species compared to the monocot pathogenic species, has been assigned to the CAZy subfamily GH43 (Table 1) that contains xylan and pectin degrading enzymes.

**Table 1:** Description of transcription profiles, number of genes identified, and main biological functions in each condition

Condition	Conditions of overexpression	# genes	Main biological function/description
0	In the presence of glucose	10	Primary metabolism; transporters
1	In the presence of PS	13	CAZy GH27/GH5/GH43; transporters; 2 transcription factors
1a	In the presence of PS and overexpressed in DS in eudicot pathogens	1	CAZy GH43
1b	In the presence of PS and overexpressed in MS in monocot pathogens	2	Sugar transport; alkaline phosphatases
2	In the presence of MS	0	NA
3	In the presence of DS	0	NA
4	In the presence of DS only in dicot pathogens	2	CAZy GH142; transmembrane protein
5	In the presence of MS only in monocot pathogens	10	CAZy GH11 (CBM1); transmembrane proteins; 2 transcription factor
6	In the presence of PS only in monocot pathogens	22	CAZy GH43/GH62 (CBM1); transporters, oxidoreductase activity; 3 transcription factors
7	In the presence of PS only in dicot pathogens	52	Unknown functions, zinc finger—nucleic acid binding; 6 transcription factors

Two orthogroups were identified as overexpressed in the presence of DS only by dicot pathogens (condition 4), and 10 orthogroups were identified as overexpressed in the presence of the MS only by monocot pathogens (condition 5). This suggests a certain level of specificity by the dicot and monocot pathogenic species. The main differences between the 2 sets of genes are the presence of specific transcription factors in the response of the monocot pathogens while the response of the dicot pathogens lacks specific transcription factors. Another difference is highlighted by differences in genes encoding for CAZy (GH142 in condition 4 and GH11 [CBM1] in condition 5). An opposite situation was observed in condition 6 compared to condition 7, where the number of orthogroups overexpressed by dicot pathogenic species was more than double of those overexpressed by monocot pathogenic species in the plant substrates (MS or DS). Both sets are rich in transcription factors, but while *C. graminicola* and *C. phormii* overexpressed several shared genes encoding for CAZymes (such as GH62, AA3_2, and 2 different genes belonging to the GH43), *C. nymphaeae* and *C. higginsianum* overexpressed only 1 (also belonging to GH43).

### Expression patterns of CAZy encoding genes are unique to each *Colletotrichum* species

**Figure 4:**
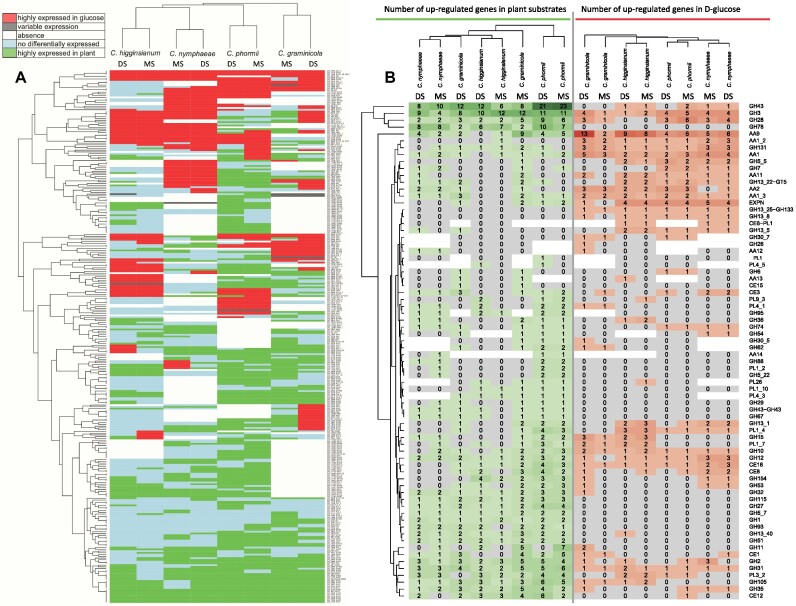
(A) Comparison of differential gene expression of CAZy ortholog groups on plant substrates (MS as monocot substrates and DS and dicot substrates) or D-glucose. The genes were binned into the following 5 categories: highly expressed in D-glucose, variable expression, absence, not differentially expressed, and lowly expressed in D-glucose for each ortholog gene(s) of each species in each specific comparison and shown in different colors. (B) Comparison of the number of CAZy genes upregulated on plant substrates (MS as monocot substrates and DS and dicot substrates) or D-glucose for the tested species. The number of highly and lowly expressed genes detected in D-glucose condition is marked in red and green, respectively. The ortholog genes missed in the specific species are indicated in white.

In contrast to the small differences in gene numbers per CAZy family, comparison of the transcriptome profiles of *C. higginsianum, C. nymphaeae, C. phormii*, and *C. graminicola* revealed high diversity between them. Based on the expression differences of CAZy genes between transcriptome of fungi growth in D-glucose and the other 2 substrates (DS and MS), the expression of the orthologous genes was clustered for the 4 fungal species (Fig. 4A).

This demonstrated that the transcriptional profiles of the same fungus grown on 2 different substrates (maize powder and sugar beet pulp) cluster together, indicating that the fungal species is more strongly associated with the expression pattern than the monocot or dicot nature of the substrate. The dicot-infecting fungal species (*C. higginsianum, C. nymphaeae*) were most similar to each other, while the 2 monocot-infecting species (*C. phormii, C. graminicola*) were more distinct. This effect seems to be mainly at the individual orthogroup level, as more similarity can be observed between the fungal species when the number of genes upregulated on plant substrates or on D-glucose was compared between the species for each CAZy family (Fig. 4B). In this comparison, the clustering of the dicot fungal-infecting species was no longer observed, suggesting strong differences in the transcriptional response of the individual species.

### Dicot-associated *Colletotrichum* spp. have more complex regulatory response to PS and revealed potential new regulatory elements

The expression patterns of *C. higginsianum, C. nymphaeae, C. phormii*, and *C. graminicola* revealed the presence of several genes encoding transcription factors (TFs) and other regulatory genes showing interesting patterns of expression (Table 2).

**Table 2:** Transcription factors and other genes involved in modulating gene expression identified in the transcriptome dataset

Condition	Conditions of overexpression	Orthogroup	Domain	Predicted/putative function
1	In presence of PS	OG_1905	Cys6Zn2 TF	Unknown/vegetative asexual development
		OG_7409	Cys6Zn2 TF	Activator of stress 1 (ASG1)/hyphal growth
5	In presence of MS only in monocot pathogens	OG_8644	Methyltransferase	Secondary metabolism
		OG_1140	Cys2His2 TF	Unknown
6	In presence of PS only in monocot pathogens	OG_6982	Methyltransferase	Unknown/putative growth control
		OG_401	Cys6Zn2 TF	Activator of purine utilization
		OG_1148	Cys6Zn2 TF	Secondary metabolism
7	In presence of PS only in dicot pathogens	OG_2149	Cys6Zn2 TF	Conidiophore development, hyphal growth
		OG_2547	SFN2 helicase	Chromatin remodeling/DNA repair
		OG_3693	Cys6Zn2 TF	Unknown
		OG_2666	Cys6Zn2 TF	Cutinase transcription factor 1 (CTF1)
		OG_2742	GATA-like TF	Development and disease
		OG_5209	E3 ubiquitin ligase	Proteasome-mediated ubiquitin-dependent protein catabolic process
		OG_7815	bZIP TF	Oxidative stress/pathogenicity
		OG_8935	GATA TF	Sensing
		OG_94	E3 ubiquitin ligase	Ubiquitin ligase/histone regulation

Surprisingly, none of them are orthologs of already characterized TFs directly involved in plant cell wall degradation, some of which are unknown, or we could not identify a clear function. Indeed, all 4 fungal species overexpressed only 2 TFs in the presence of PS (condition 1), which have a putative function in vegetative and stress growth, suggesting that the saprophytic stage of *Colletotrichum* spp. required a reshaping of the growth *modus operandi*. Interestingly, no TFs were overexpressed in the 4 fungal species growing on MS (condition 2) or DS (condition 3), matching with the CAZymes’ expression pattern where species appeared to have a higher influence than the nature of the substrate. Monocot- and dicot-associated pathogens responded differently to PS at the regulatory level. Monocot pathogens specifically overexpressed a narrow set of TFs (5 in total), mainly involved in growth control and secondary metabolism. Moreover, only monocot pathogens appeared to be partially adapted to their natural substrate as 2 TFs were overexpressed in MS only in monocot pathogenic species (condition 5) while no TFs were differentially expressed in dicot pathogenic species on DS. These 2 TFs show an interesting behavior: the methyltransferase OG_8644 is present in all 4 species but differentially expressed only in monocot-associated pathogens on MS, while the unknown Cys_2_His_2_ TF OG_1140 is present only in *Colletotrichum* spp. associated with monocots, suggesting that it has been acquired during the adaptation toward monocot hosts.

In contrast to monocot-associated pathogens, dicot pathogens had more expanded and complex regulatory responses with more than half of the total differentially expressed TFs, with no TFs specifically differentially expressed in DS (condition 4), suggesting that these strains have a less substrate specific response.

Six TFs and 3 regulatory factors were overexpressed in both plant substrates (MS and DS) only by dicot-associated pathogens (condition 7), although they are present in all 4 genomes. This evidence suggests that these regulatory genes may have lost the function to respond to plant cell walls during the process of adaptation to monocot hosts. Most of such TFs appear to have putative functions in virulence and pathogenicity. The other regulatory genes found in this category have functions in chromatin remodeling and posttranscription regulation, suggesting that the adaptation to dicot hosts also required adaptations at the posttranscriptional and translational level. Confirming this hypothesis, in this category we found several genes involved in translation process/modification, especially at the tRNA level (Supplementary Table S12). This indicates that the chromatin remodeling and the posttranslation processes are important for the dicot-associated pathogens for host interactions and/or plant cell wall interaction.

## Discussion

The ancestral *Colletotrichum* was associated with dicot plants and certain branches progressively adapted to different monocot hosts. The diversification of species inside the genus took place during the Upper (or Late) Cretaceous, 68.76 mya (103.85–45.53). This period was characterized by the ecological success of angiosperms that appeared in the fossil records (between 145 and 66 mya) [30]. Previous studies indicate that ancestral angiosperms lived in low evaporative niches during the Early Cretaceous [31] before their quick diversification in the Mid-Cretaceous [32]. During the Late Cretaceous, evolving angiosperms spread toward the poles [33] and gained ecological dominance in most of the world’s ecosystems by replacing gymnosperms in the evaporatively more demanding upper canopy [34]. In our dataset, at least 3 different events of host jumps and specialization to monocots were detected, the first when species belonging to the Graminicola complex diverged from those belonging to the Spaethianum complex around 25.23 mya (42.71–14.91), the second when *C. orchidophilum* diverged from the common ancestor of species belonging to the Acutatum complex around 14.58 mya (23.89–8.89), and the third event when *C. phormii* diverged from the closely related species *C. salicis* around 3.45 mya (6.55–1.82).

All members of the Graminicola complex are pathogenic to species belonging to the Poaceae. However, while most of the species can infect plants belonging to the Panicoideae subfamily (PACMAD clade), *C. zoysiae* is pathogenic to *Zoysia tenuifolia*, which belongs to the Chloridoideae subfamily (PACMAD clade), and *C. cereale* is pathogenic to *Poa annua*, which belongs to the Pooideae subfamily (BOP clade). The ancestor of all hosts of the Graminicola species can be placed at the crown node of BOP and PACMAD that is dated at 57 mya (75–51 mya) in the late Paleogene [19]. This event happened before the differentiation of species belonging to the Graminicola complex and those belonging to the Spaethianum complex, while the quick species diversification into Graminicola species took place between the Miocene and the Oligocene, 18.59 mya (32.56–10.62 mya), overlapping with the occupation of open habitats in Africa of their hosts that occurred in the late Eocene–early Oligocene. The Oligocene period was considerably drier than the rest of the Tertiary, and these factors might have had an effect on the decrease of the forest cover and the expansion of open habitats [35]. The second jump to monocot hosts happened when *C. orchidophilum* diverged from the ancestor in common with species belonging to the Acutatum complex around 14.58 mya (23.89–8.89). *C. orchidophilum* is host specific, infecting different species belonging to the Orchidaceae, including species belonging to *Phalaenopsis, Cycnoches, Dendrobium*, and *Vanilla*genera [11, 12, 36], covering the entire diversity of the Orchidaceae. Previous studies reported that the common ancestor of orchids was supposed to have existed much earlier, between 76 and 84 mya [37]. The last of the 3 monocot specialization events happened when *C. phormii* diverged from the closely related species *C. salicis* in the Neogene, around 3.45 mya (6.55–1.82). *C. phormii* is a worldwide-distributed pathogen of *Phormium* spp. *Dianella*-like fossils from the Eocene have been placed at the crown of the genera *Phormium* and *Dianella*, dating the divergence between these 2 genera to around 45 mya (SD = 1.0) [38], which is much earlier than the estimated appearance of *C. phormii*. Among the 3 events described, *C. orchidophilum* and *C. phormii* might have acquired a key gene or genes that allow the host jump after the appearance of the host, while the ancestor of species belonging to the Graminicola complex has evolved simultaneously with its hosts. Interestingly, all lineages of *Colletotrichum* associated with monocots show a certain level of host specificity that could reflect their more recent host jumps.

Analysis of the plant cell wall degradation-related CAZome of the different *Colletotrichum* species did not reveal large differences, especially when compared to similar studies in the genus *Aspergillus* [39, 40]. The dicot-infecting species have a higher overall number of genes encoding putative plant biomass degrading enzymes than the species with monocot hosts, which confirms results found on a previous study comparing the *C. higginsianum* and *C. graminicola* genome [6]. This is also apparent by the number of CAZy families encoding CEs, GHs, or PLs, for which the dicot-infecting species have a significantly higher number of genes, even though this difference per family is often small. In contrast, higher gene numbers per family for the monocot-infecting species are involved in xylan degradation, a prominent component of monocot cell walls. This difference between the monocot- and dicot-infecting species reflects the more diverse cell walls of dicots [41], which would require a broader set of enzymes to efficiently degrade them. A clear difference between monocot- and dicot-infecting species was found in the number of genes encoding putative pectin degrading enzymes. Pectin is a major component of dicot cell walls but nearly absent in monocots [41]. Studies of specific CAZymes in *Colletotrichum* spp. are relatively few, and they only address some of the enzymes involved in plant biomass degradation [42, 43]. Hydrogen peroxide (H_2_O_2_) may have multiple roles in plant pathogenic fungi because 2 subfamily AA5_2 alcohol oxidases have been characterized from *C. graminicola* and *C. gloeosporioides* [44, 45]. These enzymes have broad substrate ranges and oxidize aliphatic primary alcohols to the corresponding aldehydes, by simultaneously reducing oxygen to hydrogen peroxide. Although their natural substrates have not yet been identified, these enzymes were suggested to have a role in plant cell wall degradation. In addition, an AA5_2 raffinose oxidase that uses trisaccharide raffinose as its preferred substrate has been characterized from *C. graminicola* [46]. Moreover, a recent study showed that another AA5_2 paralog from *C. graminicola* oxidizes aryl alcohols to the corresponding aldehydes, thus describing aryl alcohol oxidase activity in the CAZy family AA5, which is traditionally related to AA3 glucose methanol choline (GMC) oxidoreductases [47].

Overall, the transcriptome analysis indicates a higher substrate specificity in the monocot pathogenic species *C. graminicola* and *C. phormii* while the response of the dicot pathogens does not seem to discriminate between the different plant substrates. In contrast to the low differences in gene numbers per CAZy family, comparison of the transcriptomes of *C. higginsianum, C. nymphaeae, C. phormii*, and *C. graminicola* revealed high diversity in gene expression. In *Aspergillus*, proteomic comparisons of a large number of species revealed a much higher diversity than was expected based on genome content and differences were more associated with taxonomic distance [48, 49]. These results in part match with previous studies of the production of plant biomass degrading enzymes in *Colletotrichum. C. graminicola* has been shown to produce β-glucosidase, β-xylosidase, and xylanase activity during solid-state fermentation on different plant biomass substrates. Enzyme families containing these activities (GH1, GH3, GH10, GH11, GH43) were also expressed on plant biomass in our study. Studies into the expression of specific genes revealed monomeric inducers of the responsible regulatory systems. An endopolygalacturonase encoding gene of *C. lindemuthianum* was expressed in the presence of L-arabinose and L-rhamnose [50]. Several of the CAZy genes of *Colletotrichum* have been implicated in pathogenicity [51, 52]. Transcriptome profiling of *C. graminicola* and *C. higginsianum* has revealed highly dynamic expression of CAZy genes during the infection process. For example, in *C. graminicola* and *C. higginsianum*, significant upregulation of several genes encoding cellulolytic enzymes was observed during the necrotrophic phase compared to the biotrophic phase, during the *in vitro* growth or the formation of the penetration appressorium [3, 6]. In *C. higginsianum* and *C. graminicola*, an orthologous GH131 encoding gene was highly upregulated during both biotrophic and necrotrophic phases, whereas in *C. higginsianum*, another GH131 family gene was also upregulated during appressorial penetration and biotrophic phase [53]. In addition, the corresponding recombinant GH131 proteins were demonstrated to have broad specificity toward substrates with β-1,3- and β-1,4-glucosidic linkages, and they were suggested as either breaking down the hemicellulose heteropolymeric structure or facilitating other enzymes to access cellulose [53]. In *C. fructicola*, a transcriptomic study of 4 types of infection-related structures revealed an upregulated expression of 27 CAZy genes during appressorium formation [54]. Among these genes, 14 encode for redox enzymes, with the highest enrichments from AA2 (heme-containing peroxidases) and AA5 (copper radical oxidases). Under cellophane infectious hyphae, high expression of GH7, AA9, PL1, and CBM1 family members was also detected. As in our study only a single time point was analyzed, this could explain the absence of the induction of some of these genes in our results. Previous studies have reported gene duplications within the CAZy genes in species characterized by a broad host range [16, 17]. Interestingly, different members of the CAZy family GH43 have been identified in 3 different conditions. Both results suggest that the GH43 may be an important family for plant substrate interaction and/or degradation in *Colletotrichum* species.

The expression of the TFs and other regulatory genes of *C. higginsianum, C. nymphaeae, C. phormii*, and *C. graminicola* was analyzed based on the orthogroups clustering and according to the different conditions. Unexpectedly, none of the major known TFs involved in plant biomass utilization [2] passed our requirements/cutoff, while most differentially expressed regulatory genes identified were TFs with uncharacterized function or other regulatory factors, mainly involved in chromatin remodeling. We found differentially expressed TFs specific to plant substrates, to monocot pathogenic species, and to dicot pathogenic species on both MS and DS. We did not identify differentially expressed TFs specific for the dicot substrate. Exceptions are monocot-associated pathogens, which overexpressed 1 TF and 1 methyltransferase in the monocot substrate, and 2 TFs and 1 methyltransferase in both plant substrates. This suggests that adaptation to monocots required changes not only at the transcriptional level but also at the chromatin access level. However, half of such TFs and other regulatory factors were overexpressed only by dicot pathogens, suggesting that dicot pathogens have a more complex regulation, most likely reflecting the substrate complexity of their host plants. The majority of DE TFs identified in this study do not have a clear function or have a very general role, but our results suggest that at least some of them may have a potential role in plant interaction.

Despite millions of years of divergent evolution, gene content among the species is, overall, highly similar, with the main differences being in plant biomass degradation, separating monocot and dicot pathogens. However, a much stronger level of diversity appears to occur at the transcriptional level. This can in part be assigned to the use of nonorthologous members of the same CAZy family by different *Colletotrichum* species. Our results indicate a higher substrate specificity in the monocot pathogenic species *C. graminicola* and *C. phormii* while the response of the dicot pathogenic species seems to be more associated with the general presence of plant substrates.

This work utilized genome sequences of 30 *Colletotrichum* spp., and at the time of this writing, more than 283 *Colletotrichum* spp. genomes are available at the NCBI genomes database, of which 67 were sequenced using long-read technology [55–62]. These data represent useful resources for future studies of gene family evolution and adaptation to different hosts and incorporating more diverse sampling of *Colletotrichum* spp. lineages.

## Materials and Methods

### Strains and nucleic acids purification

The genomes of 18 *Colletotrichum* species were sequenced and compared to the genomes of publicly available representative species (Table 3). Total genomic DNA was extracted using modified CTAB methods [63, 64]. Total RNA was extracted from frozen mycelium ground in a Tissue Lyser (QIAGEN) using TRIzol reagent (Invitrogen) according to the manufacturer’s instructions. RNA integrity and quantity were analyzed on a 1% agarose electrophoresis gel and with the RNA6000 Nano Assay, using the Agilent 2100 Bioanalyzer (Agilent Technologies) [65]. Further details are provided in Supplementary File S1.

**Table 3:** *Colletotrichum* spp. genomes used in this study

JGI code	Organisms	Complex	Strain	Host	Host clade	Origin
Colorb1	*Colletotrichum orbiculare*	Orbiculare	MAFF 240422	*Cucumis sativus*	Dicot	Japan
Gloci1	*Colletotrichum noveboracense*	Gloeosorioides	23	Unknown	Dicot	unknown
Colch1	*Colletotrichum chlorophyti*	none	NTL11	*Solanum lycopersicum*	Dicot	Japan
Colhig2	*Colletotrichum higginsianum*	Destructivum	IMI 349063	*Brassica rapa*	Dicot	Trinidad & Tobago
Colin1	*Colletotrichum incanum*	Spaethianum	MAFF 238712	*Raphanus sativus*	Dicot	Japan
Colto1	*Colletotrichum tofieldiae*	Spaethianum	861	*Arabidopsis thaliana*	Dicot	Spain
**Colce1**	***Colletotrichum cereale***	**Graminicola**	**CBS 129662**	***Poa annua***	**Monocot**	**USA**
**Coler1**	***Colletotrichum eremochloae***	**Graminicola**	**CBS 129661**	***Eremochloa ophiuroides***	**Monocot**	**USA**
**Colsu1**	***Colletotrichum sublineola***	**Graminicola**	**CBS 131301**	***Sorghum bicolor***	**Monocot**	**Burkina Fasso**
**Colfa1**	***Colletotrichum falcatum***	**Graminicola**	**MAFF 306170**	***Saccharum officinarum***	**Monocot**	**Japan**
Colgr1	*Colletotrichum graminicola*	Graminicola	M1.001	*Zea mays*	Monocot	USA
**Colna1**	***Colletotrichum navitas***	**Graminicola**	**CBS 125086**	***Panicum virgatum***	**Monocot**	**USA**
**Colca1**	***Colletotrichum caudatum***	**Graminicola**	**CBS 131602**	***Sorghastrum nutans***	**Monocot**	**USA**
**Colso1**	***Colletotrichum somersetensis***	**Graminicola**	**CBS 131599**	***Sorghastrum nutans***	**Monocot**	**USA**
**Colzo1**	***Colletotrichum zoysiae***	**Graminicola**	**MAFF 235873**	***Zoysia tenuifolia***	**Monocot**	**Japan**
Color1	*Colletotrichum orchidophilum*	none	IMI 309357	*Phalaenopsis sp*.	Monocot	United Kingdom
Colsa1	*Colletotrichum salicis*	Acutatum	CBS 607.94	*Salix sp*.	Dicot	Netherlands
**Colph1**	***Colletotrichum phormii***	**Acutatum**	**CBS 102054**	***Phormium sp*.**	**Monocot**	**New Zealand**
**Colgo1**	***Colletotrichum godetiae***	**Acutatum**	**CBS 193.32**	***Olea europaea***	**Dicot**	**Greece**
Colfi1	*Colletotrichum fioriniae*	Acutatum	IMI 504882	*Fragaria x ananassa*	Dicot	New Zealand
**Colac2**	***Colletotrichum acutatum s.s*.**	**Acutatum**	**CBS 112980**	***Pinus radiata***	**Dicot**	**South Africa**
**Colab1**	***Colletotrichum abscissum***	**Acutatum**	**IMI 504890**	***Citrus x sinensis***	**Dicot**	**USA**
**Collu1**	***Colletotrichum lupini***	**Acutatum**	**CBS 109225**	***Lupinus albus***	**Dicot**	**Ukraine**
**Colta1**	***Colletotrichum tamarilloi***	**Acutatum**	**CBS 129955**	***Solanum betaceum***	**Dicot**	**Colombia**
**Colco1**	***Colletotrichum costaricense***	**Acutatum**	**IMI 309622**	***Coffea sp*.**	**Dicot**	**Costa Rica**
**Colcu1**	***Colletotrichum cuscutae***	**Acutatum**	**IMI 304802**	***Cuscuta sp*.**	**Dicot**	**Dominica**
**Colpa1**	***Colletotrichum paranaense***	**Acutatum**	**IMI 384185**	***Caryocar brasiliense***	**Dicot**	**Brazil**
**Colme1**	***Colletotrichum melonis***	**Acutatum**	**CBS 134730**	***Malus domestica***	**Dicot**	**Brazil**
Colny1	*Colletotrichum nymphaeae*	Acutatum	IMI 504889	*Fragaria x ananassa*	Dicot	Denmark
Colsi1	*Colletotrichum simmondsii*	Acutatum	CBS 122122	*Carica papaya*	Dicot	Australia

Genomes produced in this work and species sequenced in this work are in bold.

### Genome sequencing, assembly, and annotation

Selected strains were sequenced using Pacific Biosciences RSII sequencer using Version C4 according to the manufacturer’s instructions. The filtered subread data were assembled using Falcon version 0.2.2 (RRID:SCR_016089) improved with finisherSC v2.0 [66] and polished with Quiver v smrtanalysis_2.3.0.140936.p5. Further details are provided in the Supplementary File S1.

For the other strains, quantified libraries were prepared for sequencing on the Illumina HiSeq sequencing platform utilizing a TruSeq paired-end cluster kit, v4. Sequencing of the flowcell was performed on the Illumina HiSeq2500 sequencer (RRID:SCR_016383). Raw reads filtered for artifact and process contamination were assembled with Velvet v1.2.10 (RRID:SCR_010755) [67] or SPAdes v3.8.2 (RRID:SCR_000131) [68]. BUSCO v5.5.0 (RRID:SCR_015008) [69] was used to search the selected genomes for 758 fungal orthologous genes (*fungi_odb10.2019–11-20* dataset) to assess the completeness of the genome sequences.

The genome sequences were annotated using the JGI annotation pipeline [70] or MAKER2 v2.31.8 annotation pipeline [71] as previously described [16]. Repetitive sequences were identified using RepeatModeler (RRID:SCR_015027) [72] and RepeatMasker (RRID:SCR_012954) [73] on the Galaxy platform (RRID:SCR_006281) [74].

### Phylogeny and divergence date estimation

A selection of 126 genomes covering the Pezizomycotina plus the genome of *Saccharomyces cerevisiae* as an outgroup were selected from the MycoCosm (RRID:SCR_005312) database (Supplementary File S1) and analyzed. The proteomes were clustered with OrthoFinder v0.4 (RRID:SCR_017118) [75], and single-copy gene families were aligned with MAFFT 7 (RRID:SCR_011811) [76] and then concatenated. A substitution model and its parameter values were selected using ProtTest 3.4 [77]. A phylogenetic tree was reconstructed using Bayesian Markov chain Monte Carlo (MCMC) analysis from the concatenated alignment under the WAG + I evolutionary model and the gamma distribution calculated using 4 rate categories and homogeneous rates across the tree. The calibrated tree was inferred by applying the RelTime method [78, 79] to the supplied phylogenetic tree whose branch lengths were calculated using the ordinary least squares method using MEGA X v10.1.7 [80].

The timetree was computed using 5 calibration point [81–87]. Further details are provided in the Supplementary File S1. The Tao method was used to set minimum and maximum time boundaries on nodes for which calibration densities were provided [88]. The evolutionary distances were computed using the Poisson correction method [89] and are in the units of the number of amino acid substitutions per site. Evolutionary analyses were conducted in MEGA X [80].

### Annotation of specific gene categories

Proteins that are transported out of the cell and into the extracellular space were identified with SignalP-4.1 [90]. Protein domains were annotated using Pfam [91] and InterPro [92] and mapped to GO terms [93]. CAZymes were annotated using CAZy pipeline [94].

Peptidases were annotated with the MEROPS database (RRID:SCR_007777), a hierarchical, structure-based classification for peptidases, organized into families and clans [95].

BLASTp (RRID:SCR_001010) [96] and RunIprScan results were used to manually identify genes encoding enzymes that are signatures of backbone secondary metabolite genes in the Ascomycota [97]: nonribosomal peptide synthetases (IPR010071, IPR006163, IPR001242), polyketide synthases (IPR013968), DMATS-family aromatic prenyltransferases (IPR017795, Pfam PF11991), and terpene synthases/cyclases (IPR008949).

Transcription factors were identified using BLASTp against NCBI nonredundant protein sequences (nr) database and the Aspergillus Genome Database (AspGD) [98]. *P* value of 1e-10 was used as a cutoff in both cases. NCBI conserved Domains Database (CCD) and EMBL Simple Modular Architecture Research Tool (SMART) (RRID:SCR_005026) [99] were used to manually assign putative function(s) to uncharacterized transcription factors.

Cys_6_Zn_2_ and Cys_2_His_2_ regulators were also analyzed by phylogenetic analyses (NJ) using orthologs of all kingdoms of known regulators involved in plant biomass degradation [2].

### Comparative genomics

#### Ortholog identification and protein cluster analyses

The Markov cluster algorithm implemented in mcl v14-137 [100] was used for the identification of protein clusters while (co-)orthologous groups were identified by Proteinortho v5.16b (RRID:SCR_024177) [101].

#### Identification of expansions and contractions of gene families associated with PS

Functional categories associated with monocot or dicot pathogenic species were identified using 2 different statistical analyses.

Disjoint sets calculated as:

Set 1 = monocot pathogens
Set 2 = dicot pathogens
if (Min Set1 > Max Set2), then term is overrepresented in Set1
if (Min Set2 > Max Set1), then term is overrepresented in Set2

Terms enriched based on Fisher’s exact test were calculated for each in each genome in the following subset: secretomes, all core proteins, secreted core proteins, all shared proteins, secreted shared proteins, all species-specific proteins, and secreted species-specific proteins. Profiles were compared to identify terms enriched only in monocot or dicot pathogens.

#### Transcriptomic analyses

A transfer experiment was performed for transcriptomics. Then, 250 mL of complete medium [102] containing 2% D-glucose in 1-L Erlenmeyer flasks was inoculated with 2.5 × 10^8^ fresh spores, harvested from a malt extract agar (MEA) medium plate, and incubated in a rotatory shaker at 25°C for 20 hours at 140 rpm. The mycelium was harvested by filtration and washed with liquid minimal medium (MM) [102] (without carbon source), and 2.5 g mycelium (wet weight) was transferred to 125-mL Erlenmeyer flasks containing 25 mL MM with 1% of maize powder (MS) or sugar beet pulp (DS) and incubated in a rotatory shaker at 25°C and 140 rpm. After preculturing and after 96 hours of incubation in MS or DS, the mycelium was harvested by vacuum filtration, dried between tissue paper, directly frozen in liquid nitrogen, and stored at −80°C [65]. All experiments were performed in triplicate. Further details are provided in the Supplementary File.

### Identification and analysis of differential gene expression

For transcriptomes, stranded complementary DNA libraries were generated using the Illumina Truseq Stranded mRNA Library Prep kit. Sequencing was performed using Illumina HiSeq2500 following a 2 × 100 indexed run recipe. RNA sequencing (RNA-seq) raw reads were assembled into consensus sequences using either Rnnotator v3.3.2 (RRID:SCR_011897) [103] or Trinity ver. 2.1.1 (RRID:SCR_013048) [104] and used as biological evidence for the gene prediction. Raw reads were filtered and trimmed for quality and contamination. Filtered RNA-seq reads from each library (Supplementary Fig. S4) were aligned to the corresponding reference genome using HISAT version 0.1.4-beta (RRID:SCR_015530) [105]. FeatureCounts (RRID:SCR_012919) [106] was used to generate the raw gene counts using genome annotations. Only primary hits assigned to the reverse strand were included in the raw gene counts (-s 2 -p –primary options). DESeq2 version 1.10.0 (RRID:SCR_015687) [107] was subsequently used to determine which genes were differentially expressed between pairs of conditions. The parameters used to call a gene differentially expressed between conditions were log2FoldChange > 2 and *P* < 0.05 (Supplementary Fig. S5). Further details are provided in the Supplementary File S1.

#### Comparative transcriptomics

A custom script *orthoexpress.py* was developed based on Proteinortho v5.16b (RRID:SCR_024177) [101] output (e-value: 1e-05; percent identity of best blast hits: 25%; minimum coverage of best blast alignments: 50%; using the synteny of the genomes as input and excluding the singletones genes) to identify groups of genes showing specific expression patterns (log2FoldChange > 2 and *P* < 0.05). Recent duplications were manually checked. In case of different behavior of paralogs, both forms of the (co-)orthologous groups were analyzed independently.

Seven logical conditions (Table 4) were established to identify genes differentially expressed in specific organisms/conditions.

**Table 4:** Conditions established for the identification of specific differentially expressed genes

Host	Dicot pathogenic			Monocot pathogenic			Monocot pathogenic			Dicot pathogenic			
species	*C. higginsianum*			*C. graminicola*			*C. phormii*			*C. nympaheae*			
Condition	Glucose vs DS	Glucose vs MS	DS vs MS	Glucose vs DS	Glucose vs MS	DS vs MS	Glucose vs DS	Glucose vs MS	DS vs MS	Glucose vs DS	Glucose vs MS	DS vs MS	Genes overexpressed in presence of:
0	↑	↑		↑	↑		↑	↑		↑	↑		Glucose
1	↓	↓		↓	↓		↓	↓		↓	↓		PS
1a	↓	↓	↑	↓	↓		↓	↓		↓	↓	↑	PS and overexpressed in DS in dicot pathogens
1b	↓	↓		↓	↓	↓	↓	↓	↓	↓	↓		PS and overexpressed in MS in monocot pathogens
2		↓	↓		↓	↓		↓	↓		↓	↓	MS
3	↓		↑	↓		↑	↓		↑	↓		↑	DS
4	↓		↑							↓		↑	DS only in dicot pathogens
5					↓	↓		↓	↓				MS only in monocot pathogens
6				↓	↓		↓	↓					PS only in monocot pathogens
7	↓	↓								↓	↓		PS only in dicot pathogens

Arrows pointing up indicate overexpressed genes, while arrows pointing down indicate downregulated genes.

## Supplementary Material

giae036_GIGA-D-23-00216_Original_Submission

giae036_GIGA-D-23-00216_Revision_1

giae036_Response_to_Reviewer_Comments_Original_Submission

giae036_Reviewer_1_Report_Original_Submission Jamie McGowan -- 12/17/2023 Reviewed

giae036_Reviewer_2_Report_Original_Submission Nicolas Lapalu -- 12/22/2023 Reviewed

giae036_Supplemental_Figures_and_Tables

## Data Availability

The genome sequencing data, assembly, and annotations are available at DDBJ/EMBL/GenBank. Genome nucleotide accession numbers, BioProject, and BioSamples are reported in Supplementary Table S1 while transcriptomic data are reported in Supplementary Table S13. All the data are also available at the JGI fungal genome portal MycoCosm [70]. All additional supporting data are available in the *GigaScience* repository, GigaDB [108].

## Additional Files

**Supplementary Fig. S1**. Time-calibrated phylogenomic tree of 123 fungal genomes belonging to the Pezizomycotina subdivision; *Saccharomyces cerevisiae* genome was used as the outgroup. Bars around each node represent 95% confidence intervals. The timetree was computed using 5 calibration points highlighted with red dots (1, 2, and 3 are fossils and 4 and 5 are estimated constraints); see details in the Materials and Methods section. Major taxonomic classes and respective crown divergent times are reported in green while the crown of *Colletotrichum* is highlighted in orange. Mya, million years ago.

**Supplementary Fig. S2**. Summary of BLASTN (e-value < 1e-3) searches of lineage-specific genes (CDS transcripts) versus the genome sequences of the closely related species. (A) Number and percentage of lineage-specific genes that lack homology in the target genome. (B) Scatterplots showing the distribution (percent query coverage and percent identity) of the top BLAST hit of each lineage-specific gene in the target genome.

**Supplementary Fig. S3**. Phylogenetic tree of selected gene families based on InterPro (IPR) domain distribution: PL-6 family—IPR039513; Transcription initiation factor IID, subunit 13–IPR003195; Aconitase, mitochondrial-like—IPR006248; PoSI-like peptidase domain—IPR034187. Red taxa indicate dicot pathogenic species, blue taxa indicate monocot pathogenic species, and purple taxa indicate *Colletotrichum* species that can infect both plant hosts. Pink boxes indicate gene lineages specific of the dicot pathogens. Number next to the nodes represents support values expressed as a percentage while thicker branches indicate a support value of 100%.

**Supplementary Fig. S4**. Correlation matrix of 9 RNA-seq libraries. Pairwise Pearson correlation coefficients (PCCs) were calculated for comparison among transcriptomes of various combinations of *Colletotrichum* spp. and substrates. Samples were hierarchically clustered with the Euclidean distance method. The color scale indicates the degree of correlation.

**Supplementary Fig. S5**. Volcano plots showing for each pairwise comparison analyzed the genes considered differentially expressed (green dots) based on log2FoldChange > 2 and *P* < 0.05.

**Supplementary File S1**. Extended version of materials and methods used.

**Supplementary Table S1**. Genomes used in this study and relative information. *Tree position refers to the order of the genomes in the phylogenetic tree shown in Fig. 1.

**Supplementary Table S2**. Summary of repetitive elements identified with RepeatModeler in the genomes analyzed.

**Supplementary Table S3**. Gene Ontology (GO) enrichment analysis. For each genome, the number of encoded proteins associated with a specific GO term is reported. The gene number differences in each GO term between monocot and dicot infecting species were statistically compared with the Wilcoxon rank-sum test and for disjoint sets (for further details, see “Identification of expansions and contractions of gene families associated with PS” in the Materials and Methods). *“Both” indicates those species capable of infecting dicot and monocot plants.

**Supplementary Table S4**. InterPro (IPR) enrichment analysis. For each genome, the number of encoded proteins associated with a specific IPR term is reported. The gene number differences in each IPR term between monocot and dicot infecting species were statistically compared with the Wilcoxon rank-sum test and for disjoint sets (for further details, see “Identification of expansions and contractions of gene families associated with PS” in the Materials and Methods). *“Both” indicates those species capable of infecting dicot and monocot plants.

**Supplementary Table S5**. Pfam protein family enrichment analysis. For each genome, the number of encoded proteins associated with a specific Pfam term is reported. The gene number differences in each Pfam term between monocot and dicot plant-infecting species were statistically compared with the Wilcoxon rank-sum test and for disjoint sets (for further details, see “Identification of expansions and contractions of gene families associated with PS” in the Materials and Methods). *“Both” indicates those species capable of infecting dicot and monocot plants.

**Supplementary Table S6**. Comparison of the gene content of 30 *Colletotrichum* species with respect to putative peptidases and their inhibitors. *“Both” indicates those species capable of infecting dicot and monocot plants.

**Supplementary Table S7**. Comparison of the gene content of 30 *Colletotrichum* species with respect to putative transporters. *“Both” indicates those species capable of infecting dicot and monocot plants.

**Supplementary Table S8**. Comparison of the gene content of 30 *Colletotrichum* species with respect to putative transcription factors. The gene number differences in each transcription factor family terms between monocot and dicot plant infecting species were statistically compared with the Wilcoxon rank-sum test. *“Both” indicates those species capable of infecting dicot and monocot plants.

**Supplementary Table S9**. Carbohydrate-Active enZYmes (CAZy) encoding gene enrichment analysis. For each genome, the number of encoded CAZy is reported. The gene number differences in each CAZy family between monocot and dicot infecting species were statistically compared with the Wilcoxon rank-sum test and for disjoint sets (for further details, see “Identification of expansions and contractions of gene families associated with PS” in the Materials and Methods). *“Both” indicates those species capable of infecting dicot and monocot plants.

**Supplementary Table S10**. Comparison of the genome content of 30 *Colletotrichum* species with respect to putative genes involved in plant biomass degradation. Overall comparison of the species with respect to relevant CAZy families. Statistical comparison of the gene number differences in each CAZy family between monocot and dicot infecting species were compared with the Wilcoxon rank-sum test for disjoint sets (for further details, see “Identification of expansions and contractions of gene families associated with PS” in the Materials and Methods). MCO = multicopper oxidase, CDH = cellobiose dehydrogenase, GMC = glucose-methanol-choline oxidoreductase, LPMO = lytic polysaccharide monooxygenases, AXE = acetyl xylan esterase, FAE = feruloyl esterase, PME = pectin methyl esterase, RGAE = rhamnogalacturonan acetyl esterase, GE = glucuronoyl esterase, HAE = hemicellulose acetyl esterase, BGL = β-glucosidase, MND = β-mannosidase, LAC = β-galactosidase, GUS = β-glucuronidase, BXL = β-xylosidase, EGL = endoglucanase, MAN = endomannanase, CBH = cellobiohydrolase, XLN = endoxylanase, XEG = xyloglucanase, AMY = α-amylase, AGD = α-glucosidase, GLA = glucoamylase, AGL = α-galactosidase, PGA = endopolygalacturonase, PGX = exopolygalacturonase, RHG = endorhamnogalacturonase, RGX = exorhamnogalacturonase, XGH = xylogalacturonase, AFC = α-fucosidase, XBH = xylobiohydrolase, AXL = α-xylosidase, INV = invertase, INU = endoinulinase, INX = exoinulinase, ABF = α-arabinofuranosidase, ABN = endoarabinanase, GAL = endogalactanase, AXH = arabinoxylan arabinofuranohydrolase, AGU = α-glucuronidase, RHA = α-rhamnosidase, UGH = unsaturated galacturonan hydrolase, ABX = exoarabinanase, URGH = unsaturated rhamnogalacturonan hydrolase, AMG = amylo-α-1,6-glucosidase, PLY = pectate lyase, PEL = pectin lyase, RGL = rhamnogalacturonan lyase. *“Both” indicates those species capable of infecting dicot and monocot plants.

**Supplementary Table S11**. List of orthogroups and orthologous expression changes among the 4 species analyzed. Empty cells are either missing genes or genes present but not considered differentially expressed. The parameters used to call a gene differentially expressed between conditions were log2FoldChange > 2 and *P* < 0.05.

**Supplementary Table S12**. List of orthogroups and main biological functions related to the genes identified based on specific expression patterns in each condition. Information is included such as fold change (positive values indicating overexpressed genes are highlighted in red while negative values indicate downregulated genes and are highlighted in blue). Conserved domains, gene families, and locus tags are also reported.

**Supplementary Table S13**. Summary of the RNA-seq libraries sequenced and analyzed in this study.

## Abbreviations

BUSCO: Benchmarking Universal Single-Copy Orthologs; CE: carbohydrate esterase CWDE: cell wall degrading enzyme; DEG: differentially expressed gene; DS: dicot substrate; GH: glycoside hydrolase; GMC: glucose methanol choline; GO: Gene Ontology; GTF: general transcription factor; IPR: InterPro; MS: monocot substrate mya: million years ago; PCW: plant cell wall; PL: polysaccharide lyase; PS: plant substrate; RNA-seq: RNA sequencing; TF: transcription factor.
