# Supplementary material for: Genome evolution and transcriptome plasticity is associated with adaptation to monocot and dicot plants in Colletotrichum fungi
Source: Gigascience. 2024 Jun 28;13:giae036. doi: 10.1093/gigascience/giae036 (PMC11212070; doi:10.1093/gigascience/giae036)
Supplement: giae036_GIGA-D-23-00216_Original_Submission [file giae036_giga-d-23-00216_original_submission.pdf]

GigaScience

Genome evolution and transcriptome plasticity associated with adaptation to monocot and dicot plants in Colletotrichum fungi.

--Manuscript Draft--

**Manuscript Number:**	GIGA-D-23-00216
**Full Title:**	Genome evolution and transcriptome plasticity associated with adaptation to monocot and dicot plants in Colletotrichum fungi.
**Article Type:**	Research
**Funding Information:**	Ministerio de Ciencia e Innovación (AEI/10.13039/501100011033) — Not applicable<br>Joint Genome Institute (10.46936/10.25585/60000617) — Not applicable<br>Ministerio de Universidades (AGL2015-66362-R) — Not applicable<br>Ministerio de Ciencia e Innovación (RTI2018-093611-B-I00) — Not applicable<br>Joint Genome Institute (10.46936/10.25585/60000725) — Not applicable
**Abstract:**	SUMMARY<br><br>Colletotrichum fungi infect a wide diversity of monocot and dicot hosts, causing diseases on almost all economically important plants worldwide. Colletotrichum is also a suitable model for studying gene family evolution on a fine scale to uncover events in the genome associated with biological changes. Here we present the genome sequences of 30 Colletotrichum species covering the diversity within the genus. Evolutionary analyses revealed that the Colletotrichum ancestor diverged in the late Cretaceous in parallel with the diversification of flowering plants. We provide evidence of independent host jumps from dicots to monocots during the evolution of Colletotrichum, coinciding with a progressive shrinking of the plant cell wall degradative arsenal and expansions in lineagespecific gene families.
**Corresponding Author:**	Riccardo Baroncelli<br>University of Bologna<br>Bologna, Emilia-Romagna ITALY
**Corresponding Author Secondary Information:**	
**Corresponding Author's Institution:**	University of Bologna
**Corresponding Author's Secondary Institution:**	
**First Author:**	Riccardo Baroncelli
**First Author Secondary Information:**	
**Order of Authors:**	Riccardo Baroncelli
	José F. Cobo-Díaz
	Tiziano Benocci
	Mao Peng
	Evy Battaglia
	Sajeet Haridas
	William Andreopoulos
	Kurt LaButti
	Jasmyn Pangilinan

Powered by Editorial Manager® and ProduXion Manager® from Aries Systems Corporation

	Anna Lipzen
	Maxim Koriabine
	Diane Bauer
	Gaetan Le Floch
	Miia R. Mäkelä
	Elodie Drula
	Bernard Henrissat
	Igor V. Grigoriev
	Jo Anne Crouch
	Ronald P. de Vries
	Serenella A. Sukno
	Michael R. Thon
**Order of Authors Secondary Information:**	
**Additional Information:**	
**Question**	**Response**
Are you submitting this manuscript to a special series or article collection?	No
**Experimental design and statistics**<br><br>Full details of the experimental design and statistical methods used should be given in the Methods section, as detailed in our Minimum Standards Reporting Checklist. Information essential to interpreting the data presented should be made available in the figure legends.<br><br>Have you included all the information requested in your manuscript?	Yes
**Resources**<br><br>A description of all resources used, including antibodies, cell lines, animals and software tools, with enough information to allow them to be uniquely identified, should be included in the Methods section. Authors are strongly encouraged to cite Research Resource Identifiers (RRIDs) for antibodies, model organisms and tools, where possible.	Yes

Powered by Editorial Manager® and ProduXion Manager® from Aries Systems Corporation

Have you included the information requested as detailed in our Minimum Standards Reporting Checklist?	
**Availability of data and materials**<br><br>All datasets and code on which the conclusions of the paper rely must be either included in your submission or deposited in publicly available repositories (where available and ethically appropriate), referencing such data using a unique identifier in the references and in the “Availability of Data and Materials” section of your manuscript.<br><br>Have you have met the above requirement as detailed in our Minimum Standards Reporting Checklist?	Yes

Powered by Editorial Manager® and ProduXion Manager® from Aries Systems Corporation

Manuscript Click here to access/download;Manuscript;Manuscript.docx

Genome evolution and transcriptome plasticity associated with adaptation to monocot and dicot plants in *Colletotrichum* fungi.

Riccardo Baroncelli[1,2,*], José F. Cobo-Díaz[3], Tiziano Benocci[4], Mao Peng[5], Evy Battaglia[5], Sajeet Haridas[6], William Andreopoulos[6], Kurt LaButti[6], Jasmyn Pangilinan[6], Anna Lipzen[6], Maxim Koriabine[6], Diane Bauer[6], Gaetan Le Floch[7], Miia R. Mäkelä[8], Elodie Drula[9,10], Bernard Henrissat[9,10,11], Igor V. Grigoriev[6,12], Jo Anne Crouch[13], Ronald P. de Vries[5], Serenella A. Sukno[2], Michael R. Thon[2,*].

[1] University of Bologna, Department of Agricultural and Food Sciences (DISTAL), Bologna, Italy.

[2] University of Salamanca, Department of Microbiology and Genetics, Institute for Agribiotechnology Research (CIALE), Villamayor, Salamanca, Spain.

[3] University of León, Department of Food Hygiene and Technology and Institute of Food Science and Technology, Leon, Spain.

[4] Austrian Institute of Technology (AIT), Center for Health and Bioresources, Tulln, Austria

[5] Utrecht University, Westerdijk Fungal Biodiversity Institute & Fungal Molecular Physiology, Fungal Physiology, Utrecht, The Netherlands

[6] United States Department of Energy, Joint Genome Institute, Lawrence Berkeley National Laboratory, Berkeley, CA, USA

[7] University of Brest, Laboratory of Biodiversity and Microbial Ecology (LUBEM), IBSAM, ESIAB, EA 3882, Plouzané, France

[8] University of Helsinki, Department of Microbiology, Faculty of Agriculture and Forestry, Helsinki, Finland.

[9] University of Aix-Marseille (AMU), UMR 7257, Architecture et Fonction des Macromolécules Biologiques, The French National Centre for Scientific Research (CNRS), Marseille, France

[10] The French National Institute for Agricultural Research (INRA), USC 1408 AFMB, Marseille, France

[11] King Abdulaziz University, Department of Biological Sciences, Jeddah, Saudi Arabia

[12] University of California Berkeley, Department of Plant and Microbial Biology, Berkeley, USA

[13] United States Department of Agriculture, Mycology and Nematology Genetic Diversity and Biology Laboratory, Agricultural Research Service, Beltsville, USA

[*] Correspondence should be addressed to R.B. (riccardo.baroncelli@unibo.it) or M.R.T. (mthon@usal.es).

Abstract

Background: *Colletotrichum* fungi infect a wide diversity of monocot and dicot hosts, causing diseases on almost all economically important plants worldwide. *Colletotrichum* is also a suitable model for studying gene family evolution on a fine scale to uncover events in the genome associated with biological changes.

Results: Here we present the genome sequences of 30 *Colletotrichum* species covering the diversity within the genus. Evolutionary analyses revealed that the *Colletotrichum* ancestor diverged in the late Cretaceous in parallel with the diversification of flowering plants. We provide evidence of independent host jumps from dicots to monocots during the evolution of *Colletotrichum*, coinciding with a progressive shrinking of the plant cell wall degradative arsenal and expansions in lineage-specific gene families. Comparative transcriptomics of four species adapted to different hosts revealed similarity in gene content but high diversity in the modulation of their transcription profiles on different plant substrates. Combining genomics and transcriptomics we identified a set of core genes such as specific transcription factors, putatively involved in plant cell wall degradation.

Conclusions: These results indicate that the ancestral *Colletotrichum* were associated with dicot plants and certain branches progressively adapted to different monocot hosts, reshaping the gene content and its regulation.

Keywords: Fungal genomics, comparative transcriptomics, fungal evolution, anthracnose, plant cell walls

Introduction

The plant cell wall (PCW) consists of many different polysaccharides that are attached not only to each other through a variety of linkages providing the main strength and structure for the PCW. In addition, PCWs are determinants of immune responses since modification of their composition affect disease resistance and fitness on plants [1–3].

The PCW can be seen as one of the first layers of defense where the arms race between the pathogen and the host takes place, but also as a complex ecological niche where the fungi (pathogenic as well as mutualistic) retrieve most of the nutrients from the host during the interaction. To release the monomers present in these complex plant structures, fungi need to simultaneously secrete several plant biomass degrading enzymes, mainly associated with hydrolytic and oxidative functions [2]. Plants protect themselves against degradation of their cell walls by producing proteins that inhibit microbial cell wall degrading enzymes (CWDEs), e.g., inhibitors of pectin-degrading enzymes are common in dicots and non-commelinoid monocots, and inhibitors of xylan-degrading enzymes are common in the Poaceae [4]. The production of these inhibitors by plants has, in turn, driven the evolution of some CWDE groups of phytopathogenic fungi toward inhibitor-resistant enzymes [5]. In some phytopathogenic fungi, there is evidence for production of different amounts of specific CWDEs, depending on whether the plant host is a monocot or dicot [6–8].

Some *Colletotrichum* species show a one-to-one relationship with a specific host while other species infect a wide range of hosts [6,9–12]. The biological diversity of *Colletotrichum* and the presence of very closely related species with different host ranges makes this genus an excellent model to investigate genomic signatures associated with the evolution of biological characters important for host interactions such as those involved in PCW degradation.

Since the first genome sequences of fungi became available, researchers have been analyzing gene content and genomic features to find associations that may explain the differences in fungal lifestyles and varying patterns are beginning to emerge [6,13,14]. In contrast, gene loss or gain in families such as those encoding CAZymes and proteases could be associated with host range in *Colletotrichum* species [15]. The similar repertoires of CAZymes and secreted proteases found in relatively distant members of the *C. acutatum* and *C. gloeosporioides* species complexes suggest a recent and independent acquisition of this enzymatic arsenal or a progressive loss during the host specialization

process [6,15,16]. While genome studies are useful tools to identify putative genes and to perform evolutionary analyses, transcriptomic data is required to better understand the genes involved in a complex process such as PCW interaction.

Plant pathogenic fungi have a close interaction with the PCW and plants have evolved to recognize external attacks through the degradation of the PCW itself. This is especially true for hemibiotrophic plant pathogens as they interact with the PCW twice: initially when they enter the cell and later when they gain nutrients from it. This complexity is reflected by the wide arsenal of CAZymes encoded by *Colletotrichum* spp. being one of the most diverse in the fungal kingdom.

In this work, we used comparative genomics and transcriptomics to identify genes involved in the interaction between *Colletotrichum* spp. and the plant substrates (which are mainly composed by PCW), as well as evolutionary analyses to gain a better understanding of adaptation and specialization of these fungi to different plant substrates. Phylogenetic analyses revealed that the ancestral *Colletotrichum* was associated with dicots and that at least 3 independent jumps to monocots occurred. We also found that monocot associated *Colletotrichum* species have undergone specific gene losses in PCW degrading enzyme families and expansions in lineage specific genes. Comparing four different *Colletotrichum* species we also found that, despite millions of years of divergent evolution, they have maintained highly similar gene content, with exceptions in the CAZymes and proteases, and show strong differences in gene modulation associated with different host substrates.

Results

The common ancestor of *Colletotrichum* parasitized dicots and specific lineages jumped independently to monocots.

In this study, we present a comparative genomic analysis of 30 species from the genus *Colletotrichum*. Eleven of these (*C. cereale*, *C. eremochloae*, *C. sublineola*, *C. graminicola*, *C. falcatum*, *C. navitas*, *C. caudatum*, *C. somersetensis*, *C. zoysiae*, *C. orchidophilum* and *C. phormii*) are pathogens specialized to different taxonomic groups of monocots; seventeen (*C. orbiculare*, *C. noveboracense* , *C. higginsianum*, *C. tofieldiae*, *C. salicis*, *C. godetiae*, *C. acutatum sensu stricto*, *C. fioriniae*, *C. abscissum*, *C. lupini*, *C. tamarilloi*, *C. costaricense*, *C. cuscutae*, *C. paranaense*, *C. melonis, C. nymphaeae* and *C.*

simmondsii) have been associated only with dicots while two of them (*C. chlorophyti* and *C. incanum*) are capable of infecting plants that belong to both groups.

The analyzed genomes showed a large variation in size, ranging from 44.20 Mb in *C. caudatum* to 89.65 Mb in *C. orbiculare* (Figure 1). While a large variation at the genus level was already reported [17] (more than 50% in our dataset) these results highlight an unexpected variation of more than 30 Mb (39%) between two closely related species such as *C. cuscutae* and *C. paranaense*. These two species belong to the Acutatum species complex and have been recognized as separate taxa only recently. As a general trend, species with bigger genomes are characterized by a lower GC content. This evidence coupled with the lack of correlation between genome size and number of predicted genes suggest that the genome size in *Colletotrichum* is mainly correlated with the amount of non-coding DNA (Figures 1 and 2). Phylogenomic analyses calibrated with three fungal fossils show age estimates for *Colletotrichum* spp. and enable the identification of time frames of specific evolutionary events (Figure 1).

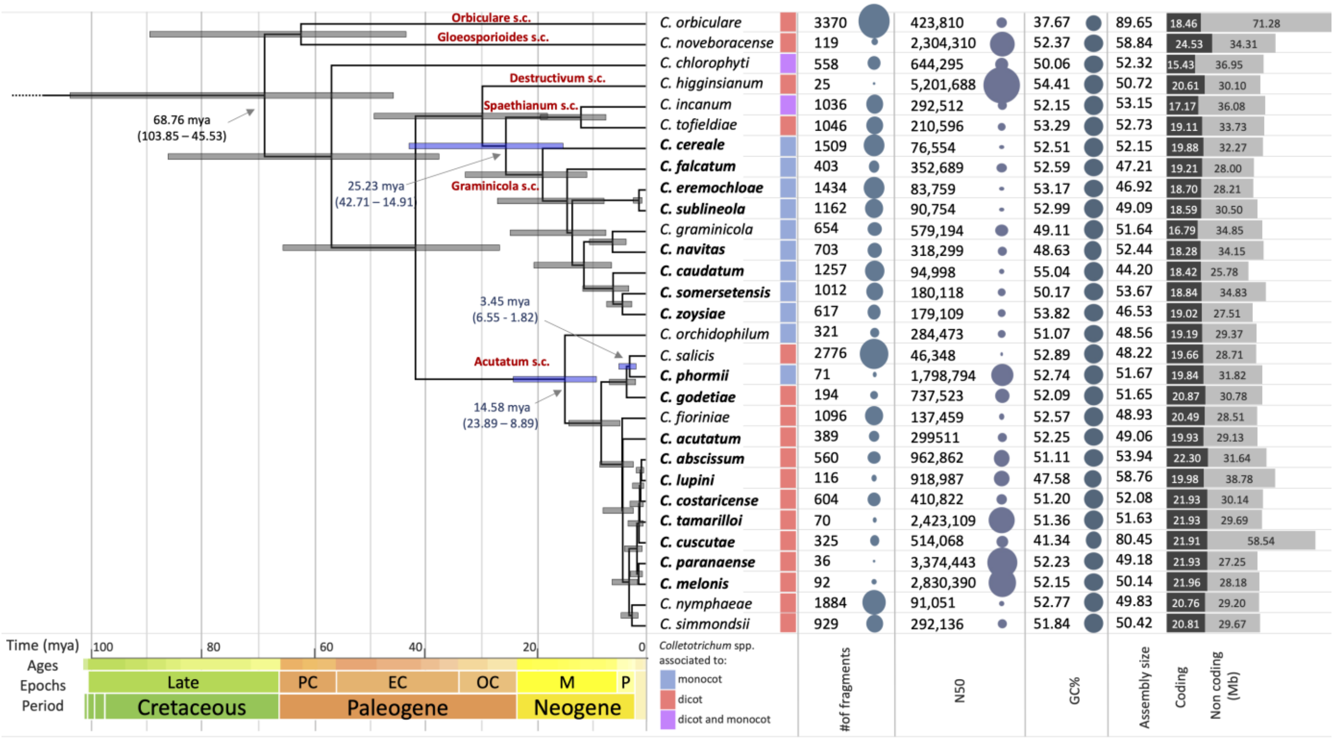

Figure 1. A timetree inferred by the RelTime method to the *Colletotrichum* phylogenomic tree. The branch lengths were calculated using the Ordinary Least Squares method. All nodes are supported by Bayesian posterior probability of 1.00. Bars around each node represent 95% confidence intervals and light blue bars represent the three host jumps from dicot to monocot. This analysis involved 127 amino acid sequences and a total of 124023 sites. *Colletotrichum* species

complexes are indicated in red. Genomes sequenced in the present study are highlighted in bold. On the right side four bubble plots illustrating assembly size, GC content and assembly fragmentation parameters (number of contigs and N50 value) and are reported in the right side. The bubble sizes have been scaled to each panel and are not comparable across panels. Gray bar diagram on the right reports the size of coding and non-coding regions.

Colletotrichum species diverged from members of the closest related genus *Verticillium* in the late Jurassic around 136.43 million years ago (mya) (186.35 - 99.88) (Supplementary 1). The diversification of species within the genus, based on the estimation of divergence between the two most distantly related species *C. orbiculare* and *C. abscissum*, took place during the Upper (or Late) Cretaceous period, 68.76 mya (103.85 – 45.53). These results suggest that the common ancestor of *Colletotrichum* was associated with dicots and at least three independent host jumps between dicots and monocots took place during the evolution of this pathogen. The first took place in the Paleogene (around 25 mya) when species of the Graminicola complex diverged from those belonging to the Spaethianum complex. Interestingly, the diversification of *Colletotrichum* species adapted to plant species belonging to the Poaceae, happened around 20 mya, coinciding with the expansions of grasses from their water-bank habitat into open tracts and their diversification [18]. The second happened in around 15 mya when *C. orchidophilum* diverged from the ancestor of the Acutaum species complex. The third host jump occur in the Neogene around 3.5 mya when the flax pathogenic species *C. phormi* diverged from its closest related species *C. salicis.*

Colletotrichum species associated with monocots have gone through expansions of lineage specific genes and losses of degradative enzymes and other conserved functions.

To examine core features shared by all *Colletotrichum* species, by complexes, by individual species, as well as features specific of dicot and monocot associated species, all predicted proteomes were clustered into groups of orthologous genes (Figure 2A). This approach enabled the identification of the core, shared and species-specific proteins and orthologs only present in species associated with dicot or monocot hosts. Enrichment analyses of the core, shared and lineage specific (secreted and non-secreted) protein encoding genes did not identify functional category or gene family expansions associated with host range. Considering that the analyses carried out are affected by the sampling, as

closely related species are likely to have more shared genes compared to species that are more distant from others, our analyses also highlight that monocot pathogenic species have generally more lineage-specific genes compared to dicot pathogenic species (Figure2A and 2C). While no orthogroups specific to the monocot pathogenic species were identified, we found three orthogroups only present in those species capable of infecting dicot plants. These were OG0010350, with one or two copies of the gene present in all dicot pathogenic species and in *C. incanum* and characterized as a secreted β-glucosidase (CAZy - GH3/FN3), OG0010637 with one or two copies of the gene present in all dicot pathogenic species and in *C. incanum* and characterized as a secreted protein with unknown function containing a (FAD)-binding domain, and OG0011101 present in all dicot pathogenic species and in those that have been associated with dicot and monocot and described as an α-1,2-mannosidase (CAZy - GH92).

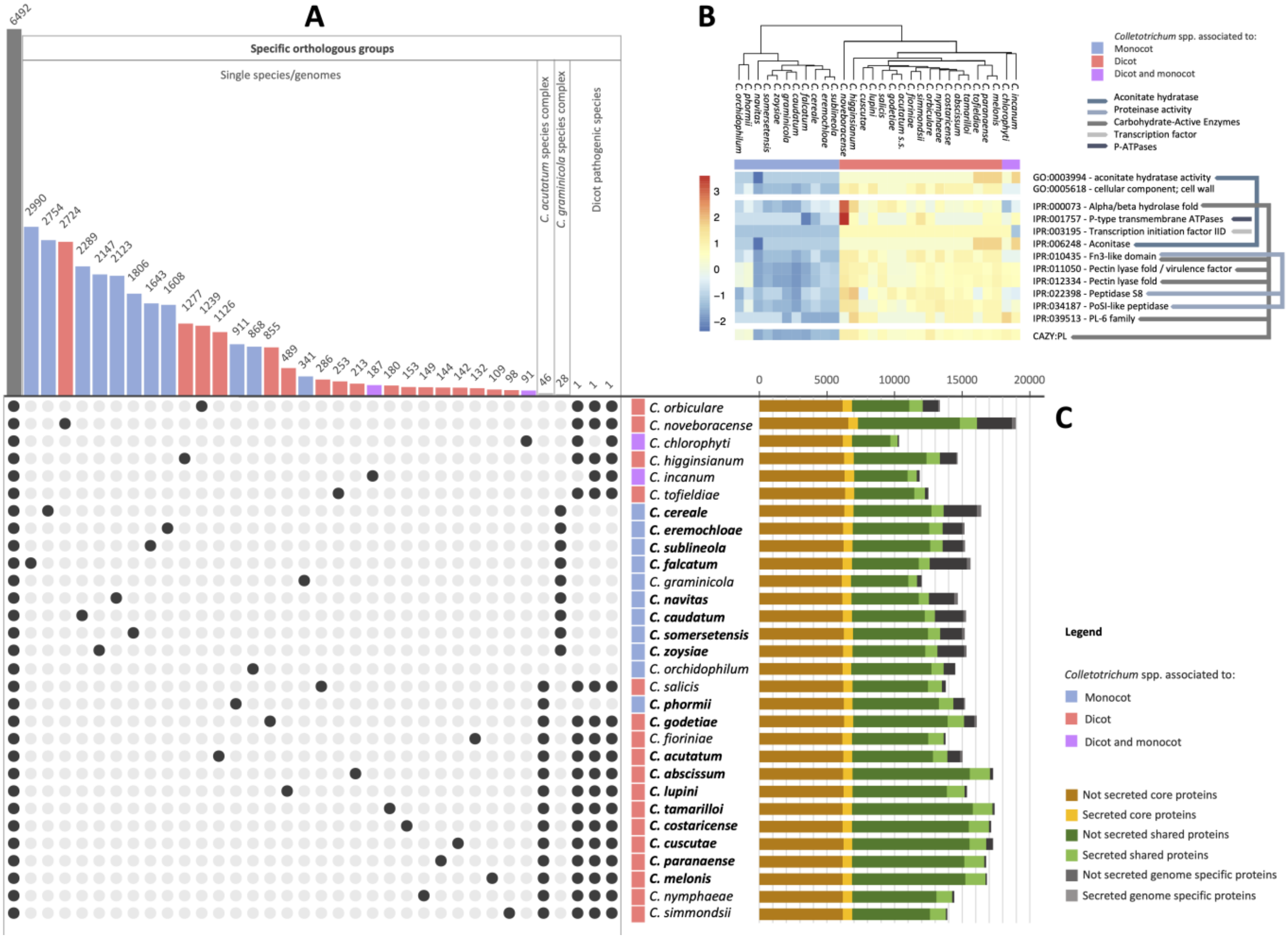

Figure 2. Comparative genomic analysis of *Colletotrichum* species. (**A**) UpsetR plot of the protein clustering analysis. Bars in the upper side represent the number of orthogroups shared by the species highlighted by the black dots reported in the bottom side. (**B**) Hierarchical clustering of disjoint sets of terms and gene families identified in *Colletotrichum* species

associated with monocots and dicots hosts. Gene Ontology and InterPro terms corresponding to the rows are reported on the right; coloured lines connect overlapping terms. Hierarchical clustering of genes and species was performed and visualized using the UPGMA algorithm. Overrepresented (orange to red) and underrepresented functional domains (blue). (**C**) Bar diagrams showing the number of proteins shared with all included species (in yellow), shared with at least two but not all (in green) and those found in only one species (in grey). The light shading indicates for each group the portion of proteins predicted to be secreted.

Analyses of functional annotations highlighted two gene ontology (GO), 12 InterPro (IPR) terms and two gene families expanded in dicot associated species compared to the monocot associated species (Figure2B). No terms were expanded in monocot associated *Colletotrichum* spp. confirming the pattern observed in the analyses based on protein similarity and the two species capable of infecting both hosts (*C. chlorophyti* and *C. incanum*) cluster with the dicot associated pathogens. As many IPR and GO terms overlap, the results were manually inspected to avoid redundancy.

Overall, terms identified as expanded in dicot associated pathogens could be clustered into five functional groups (Figure 2B): 1) aconitases are genes encoding for enzymes that catalyse the stereo-specific isomerization of citrate to isocitrate in the Krebs cycle; while dicot pathogens have three or four copies of this gene, monocot pathogens have only two. 2) P-ATPases are proteins that are involved in transport of a variety of different compounds. 3) Transcription initiation factor IID is a general transcription factor (GTF) involved in accurate initiation of transcription by RNA polymerase II. 4) Serine proteases belonging to the MEROPS peptidase family S8. 5) Several terms identified, such as the alpha/beta hydrolase fold, the pectin lyase fold, the PL6 family domains as well as others are associated with CAZymes.

Dicot infecting species have a higher overall number of genes encoding putative plant biomass degrading enzymes than the species with monocot hosts (Suppl7_CAZYmes_PBD.xlsx), which confirms previous studies [6]. This is also clear by the number of CAZy families encoding carbohydrate esterases (CE), glycoside hydrolases (GH) or polysaccharide lyases (PL), for which the dicot infecting species have a significantly higher number of genes. In contrast, higher gene numbers per family for the monocot infecting species are only present in CE1, GH10, GH11, GH13_1, GH45 and GH62. Interestingly CE1, GH10, GH11 and GH62 are all involved in xylan degradation, a prominent component of monocot cell walls. CAZy families encoding putative pectinolytic enzymes have higher

numbers of genes in the dicot infecting species, such as CE8, CE12, GH28, GH43, GH52, GH53, GH78, GH88, GH93, PL1, PL3, PL11 and PL26. However, also CAZy families with putative enzymes targeting lignin (AA1), cellulose (GH1, GH3, GH5, GH7) and hemicellulose (CE16, GH12, GH27, GH36, GH74, GH115) are enriched in the dicot infecting species. At the individual species level, *C. noveboracense* stands out with an increased number of genes in several CAZy families (AA1_3, CE1, GH1, GH2, GH7, GH28, GH43, GH78). The *Colletotrichum* species lack the subfamily AA1_1 *sensu stricto* laccases but possess putative laccase-like multicopper oxidase encoding genes from the subfamilies AA1_2 and AA1_3. A previously described laccase (*lac2*), which is involved in melanisation in appressorial cells of *C. orbiculare* [19], is categorized as a member of family AA1 without a subfamily division, whereas a *C. orbiculare lac1* which does not have a role in melanin biosynthesis or pathogenicity [19], is catalogued to AA1_3. For three of the species, *C. acutatum*, *C. higginsianum* and *C. graminicola*, growth profiles on plant biomass related substrates are available in the FUNG-GROWTH database (https://www.fung-growth.org/). Comparison of the CAZome of these three species (Suppl7_CAZYmes_PBD.xlsx) to their growth profiles did not provide clear correlations. Growth on xylan, galactomannan (guar gum) and inulin is relatively poor for *C. higginsianum* compared to the other two species, but no strong reduction in xylanolytic, mannanolytic or inulinolytic genes can be found in its genome. This evidence also suggests that the CAZyme content in the genome can only partially explain its degradative capability.

To confirm these results and to gain a better understanding on the evolution of the genes identified using both approaches (similarity-based protein clustering and protein terms enrichment) further analyses were carried out. Results of selected CAZy families (GH3 and GH92), aconitases and transcription initiation factors IID (Suppl11_Evo_genefamilies) revealed gene losses in the monocot associated species lineages.

Transcriptome profiles on different plant substrates reveal strong variation among species.

To identify genes involved in the interaction with the PCW, we performed a transcriptome analysis of four reference species, two dicot pathogens: *C. higginsianum*, *C. nymphaeae* and two monocot pathogens: *C. graminicola* and *C. phormii*, on three different substrates: D-glucose, sugar beet pulp (dicot substrate: DS) and maize powder (derived from complete plants without cobs as monocot

substrate: MS). These substrates differ in sugar/polysaccharide composition with, sugar beet pulp being rich in cellulose, pectin and xyloglucan [21], while maize powder is rich in cellulose and hemicellulose, particularly glucuronoarabinoxylan [22]. Both plant substrates have been used as valuable waste biomass for industrial applications [23–25] and therefore largely used as substrates in similar studies to address the microbial degradation performance/requirements [26–28].

The four species show different evolutionary histories and genetic distances with *C. phormii* and *C. nymphaeae* being closely related members of the same complex, but associated with monocot and dicot hosts, respectively. *C. higginsianum*, *C. phormii* and *C. nymphaeae* have similar patterns of gene expression when the pairwise comparisons of transcriptome patterns are plotted in a principal component analysis (PCA; Figure 3A). In these three species the comparison of genes differentially expressed in DS vs MS show a lower diversity compared to the one highlighted in the comparison of genes differentially expressed in both substrates vs. D-glucose (Figure 3A). This pattern is also confirmed by the overall number of differentially expressed genes (DEGs), where *C. higginsianum*, *C. phormii* and *C. nymphaeae* have the lowest number of both up- and downregulated DEGs in DS vs MS while *C. graminicola* has a comparable number of DEGs in other pairwise comparisons (Figure 3B). The differences shown by *C. graminicola* might reflect the longer evolutionary history of association with its host as well as the differences in plant substrate composition between the hosts. Among the four species, *C. nymphaeae* regulate differentially more genes compared to the other species.

To better understand the specificity of the response to different substrates, we identified species specific genes overexpressed in presence of D-glucose, dicot substrate (DS), monocot substrate (MS), plant substrate (PS: as those genes overexpressed in presence of both DS and MS), as well as those shared among all four species, among the dicot pathogens and among the monocot pathogenic species (Figure 3C and 3D). Results highlighted a strong specific response by the four species, as the majority of the DEGs are not shared between the four genomes but are specific for each organism.

Comparative analysis of enrichment profiles highlighted five terms enriched among overexpressed genes in dicot pathogens on DS (condition 7), all of which (GO:0000981, GO:0006355, IPR001138, IPR036864, PF00172) are associated with Zn(2)-Cys(6) fungal-type DNA-binding domain and transcription regulation. As no other enrichment was identified we performed a manual annotation of

genes identified in Figure 3D. As a result, more than one third of all genes identified (32/112) were assigned to three major groups, i.e., transporters, CAZymes and transcription factors.

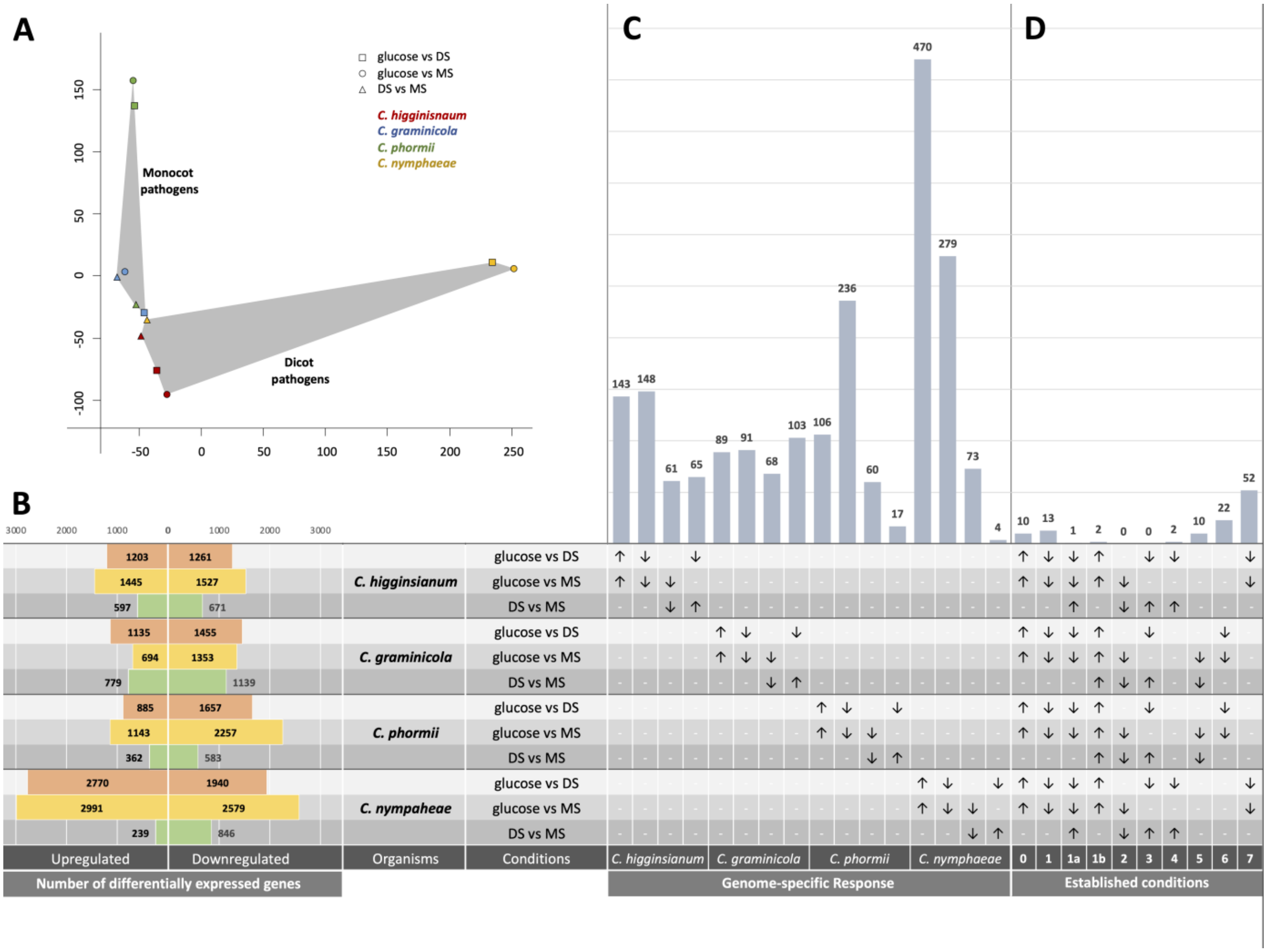

Figure 3. Comparative transcriptomic analysis of selected *Colletotrichum* species (*C. higginsianum*, *C. graminicola*, *C. phormii* and *C. nymphaeae*) on three on different carbon sources: D-glucose, sugar beet pulp (as dicot substrate: DS) and maize powder (as monocot substrate: MS). **(A)** Principal components analysis (PCA) of differentially expressed genes in all conditions analysed; grey areas highlight patterns of monocot and dicot pathogens. **(B)** Number of differentially expressed genes of each *Colletotrichum* species and in each condition analysed. **(C&D)** Genome specific response represented as the number of genes differentially expressed. For each pairwise comparison and species over expressed genes are indicated by an arrow pointing up while those under expressed are indicated by an arrow pointing down **(C)** Number of genes overexpressed in D-glucose for each genome are reported in the first column on the left, those overexpressed in PS are reported in the second column, those overexpressed in MS are reported in third column and those overexpressed in DS are reported in forth column. (**D**) Numbers of genes showing the same expression patterns in the established conditions as described in the materials and methods section.

We identified ten orthologous genes overexpressed in the presence of D-glucose compared to plant substrate (condition 0). Among these, four are transporters, three are associated with primary metabolism (such as citrate and fatty acid synthase and sorbitol dehydrogenase), one is a secreted flavoenzyme and two are secreted proteins of unknown function. Sixteen orthologous genes in each species were upregulated in the presence of the plant substrates (condition 1, 1A and 1B). In this set we identified four transporters, two transcription factors, three genes belonging to CAZy families GH27, GH5_16 and GH43, and one subclass M28 peptidase. Interestingly one orthogroup (OG_12813) assigned to condition 1a and therefore to genes overexpressed in the presence of plant substrate by all four species but more overexpressed in dicot pathogenic species compared to the monocot pathogenic species, has been assigned to the CAZy subfamily GH43 (Table 1) that contains xylan and pectin degrading enzymes.

Table 1. Description of transcription profiles, number of genes identified and main biological functions in each condition.

Condition	Conditions of overexpression	# genes	Main biological function / description
0	in the presence of glucose	10	primary metabolism; transporters
1	in the presence of PS	13	Cazy GH27 / GH5 / GH43; transporters; 2 transcription factors
1a	in the presence of PS and overexpressed in DS in eudicot pathogens	1	Cazy GH43
1b	in the presence of PS and overexpressed in MS in monocot pathogens	2	Sugar transport; alkaline phosphatases
2	in the presence of MS	0	NA
3	in the presence of DS	0	NA
4	in the presence of DS only in dicot pathogens	2	Cazy GH142; transmembrane protein
5	in the presence of MS only in monocot pathogens	10	Cazy GH11(CBM1); transmembrane proteins; 2 transcription factor
6	in the presence of PS only in monocot pathogens	22	Cazy GH43 /GH62(CBM1); transporters, oxidoreductase activity; 3 transcription factors
7	in the presence of PS only in dicot pathogens	52	Unknown functions, Zinc finger – nucleic acid binding; 6 transcription factors

* PS: plant substrate; MS: monocot; DS: dicot substrate.

Two orthogroups were identified as overexpressed in the presence of dicot substrate (DS) only by dicot pathogens (condition 4) and ten orthogroups were identified as overexpressed in the presence of the monocot substrate (MS) only by monocot pathogens (condition 5). This suggests a certain level

of specificity by the dicot and monocot pathogenic species. The main differences between the two sets of genes are the presence of specific transcription factors in the response of the monocot pathogens while the response of the dicot pathogens lacks specific transcription factors. Another difference is highlighted by differences in genes encoding for CAZy (GH142 in condition 4 and GH11 (CBM1) in condition 5). An opposite situation was observed in condition 6 compared to condition 7, where the number of orthogroups overexpressed by dicot pathogenic species is more than double of those overexpressed by monocot pathogenic species in the plant substrates (MS or DS). Both sets are rich in transcription factors, but while *C. graminicola* and *C. phormii* overexpressed several shared genes encoding for CAZymes (such as GH62, AA3_2 and two different genes belonging to the GH43), *C. nymphaeae* and *C. higginsianum* overexpressed only one (also belonging to GH43).

Expression patterns of CAZy encoding genes are unique to each *Colletotrichum* species.

In contrast to the small differences in gene numbers per CAZy family, comparison of the transcriptome profiles of *C. higginsianum*, *C. nymphaeae*, *C. phormii* and *C. graminicola* revealed high diversity between them. Based on the expression differences of CAZy genes between transcriptome of fungi growth in D-glucose and other two substrates (DS and MS), the expression of the orthologous genes was clustered for the four fungal species (Figure 4A).

This demonstrated that the transcriptional profiles of the same fungus grown on two different substrates (maize powder and sugar beet pulp) cluster together, indicating that the fungal species is more strongly associated with the expression pattern than the mono- or dicot nature of the substrate. The dicot infecting fungal species (*C. higginsianum*, *C. nymphaeae*) were most similar to each other, while the two monocot infecting species (*C. phormii*, *C. graminicola*) were more distinct. This effect seems to be mainly at the individual orthogroup level, as more similarity can be observed between the fungal species when the number of genes that were upregulated on plant substrates or on D-glucose were compared between the species for each CAZy family (Figure 4B). In this comparison, the clustering of the dicot fungal infecting species was no longer observed, suggesting strong differences in the transcriptional response of the individual species.

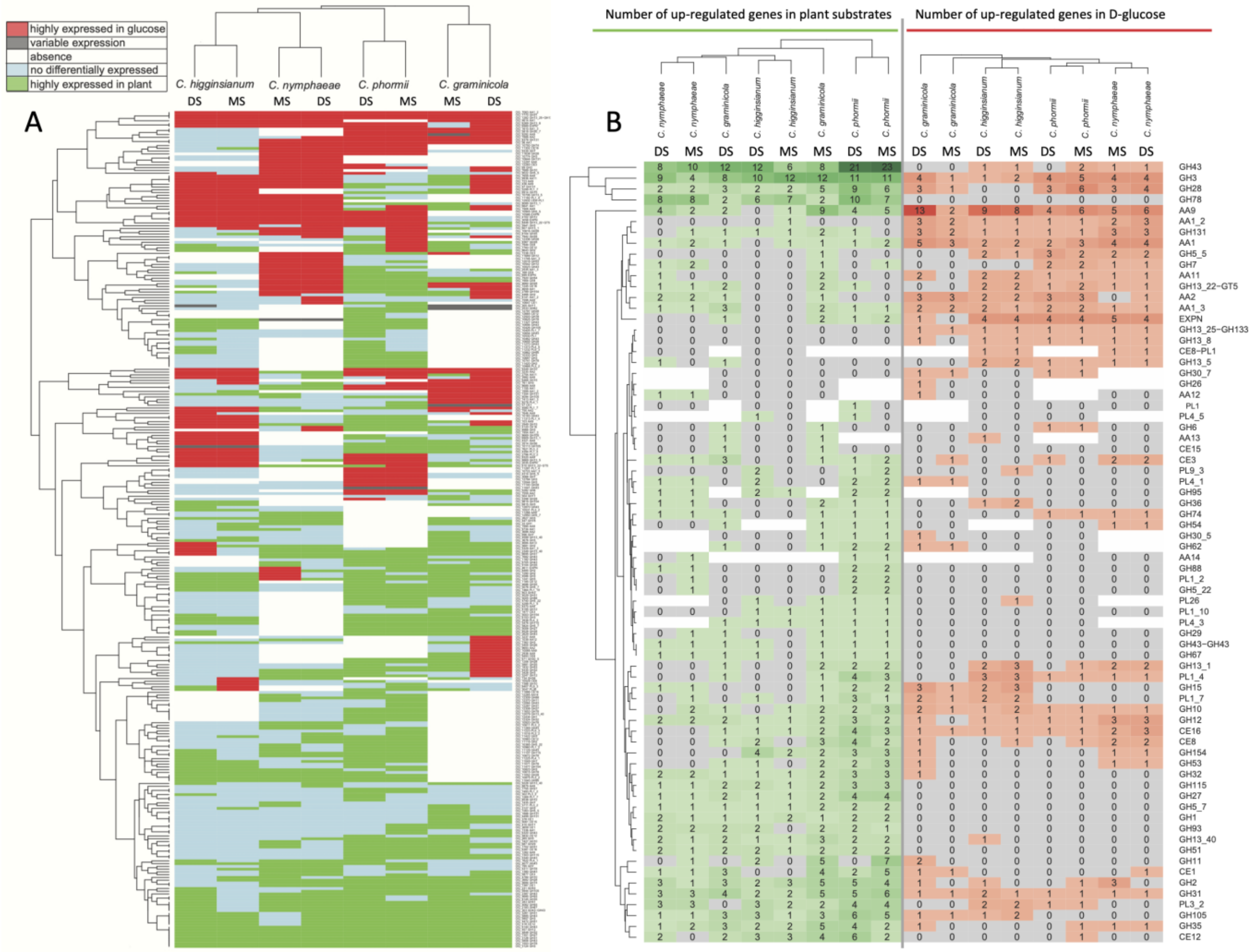

Figure 4. (A) Comparison of differential gene expression of CAZy ortholog groups on plant substrates (MS as monocot substrates and DS and dicot substrates) or D-glucose. The genes were binned into the following five categories: highly expressed in D-glucose, variable expression, absence, not differentially expressed and lowly expressed in D-glucose for each ortholog gene(s) of each species in each specific comparison and shown in different colours on the figure. **(B)** Comparison of the number of CAZy genes upregulated on plant substrates (MS as monocot substrates and DS and dicot substrates) or D-glucose for the tested species. The number of highly and lowly expressed genes detected in D-glucose condition were marked with red and green, respectively. The ortholog genes missed in the specific species were indicated with white colour.

Dicot associated *Colletotrichum* spp. have more complex regulatory response to PS and revealed potential new regulatory elements

The expression patterns of *C. higginsianum*, *C. nymphaeae*, *C. phormii* and *C. graminicola* revealed the presence of several genes encoding transcription factors (TFs) and other regulatory genes showing interesting patterns of expression (Table 2).

Table 2. Transcription factors and other genes involved in modulating gene expression identified in the transcriptome dataset.

Condition	Conditions of overexpression	Orthogroup	Domain	Predicted/putative function
1	in presence of PS	OG_1905	Cys6Zn2 TF	Unknown/vegetative asexual development
		OG_7409	Cys6Zn2 TF	Activator of stress 1 (ASG1)/hyphal growth
5	in presence of MS only in monocot pathogens	OG_8644	Methyltransferase	Secondary metabolism
		OG_1140	Cys2His2 TF	Unknown
6	in presence of PS only in monocot pathogens	OG_6982	Methyltransferase	Unknown/putative growth control
		OG_401	Cys6Zn2 TF	Activator of purine utilization
		OG_1148	Cys6Zn2 TF	Secondary metabolism
7	in presence of PS only in dicot pathogens	OG_2149	Cys6Zn2 TF	Conidiophore development, hyphal growth
		OG_2547	SFN2 helicase	Chromatin remodeling/DNA repair
		OG_3693	Cys6Zn2 TF	Unknown
		OG_2666	Cys6Zn2 TF	Cutinase transcription factor 1 (CTF1)
		OG_2742	GATA-like TF	Development and disease
		OG_5209	E3 Ubiquitin ligase	Proteasome-mediated ubiquitin-dependent protein catabolic process
		OG_7815	bZIP TF	Oxidative stress/pathogenicity
		OG_8935	GATA TF	Sensing
		OG_94	E3 Ubiquitin ligase	Ubiquitin ligase/histone regulation

* PS: plant substrate; MS: monocot; DS: dicot substrate.

Surprisingly none of them are orthologs of already characterized TFs directly involved in plant cell wall degradation, some of which are unknown, or we could not identify a clear function. Indeed, all four fungal species overexpressed only two TFs in presence of PS (condition 1) which have putative function in vegetative and stress growth suggesting that the saprophytic stage of *Colletotrichum* spp. required a re-shaping of the growth *modus operandi*. Interestingly no TFs were overexpressed in the four fungal species growing on MS (condition 2) or DS (condition 3), matching with the CAZymes expression pattern where species appeared to have a higher influence than the nature of the substrate. Monocot and dicot associated pathogens responded differently to PS at the regulatory level. Monocot pathogens specifically overexpressed a narrow set of TFs (five in total), mainly involved in growth control and secondary metabolism. Moreover, only monocot pathogens appeared to be partially adapted to their natural substrate as two TFs are overexpressed in MS only in monocot pathogenic species (condition 5) while no TFs were differentially expressed in dicot pathogenic species on DS. These two TFs show an interesting behaviour: the methyltransferase OG_8644 is

present in all four species but differentially expressed only in monocot associated pathogens on MS, while the unknown Cys_2His_2 TF OG_1140 is present only in *Colletotrichum* spp. associated with monocots, suggesting that it has been acquired during the adaptation toward monocot hosts.

In contrast to monocot associated pathogens, dicot pathogens had more expanded and complex regulatory responses with more than half of the total differentially expressed TFs, with no TFs specifically differentially expressed in DS (condition 4), suggesting that these strains have a less substrate specific response.

Six TFs and three regulatory factors were overexpressed in both plant substrates (MS and DS) only by dicot associated pathogens (condition 7), although they are present in all four genomes. This evidence suggests that these regulatory genes may have lost the function to respond to plant cell walls during the process of adaptation to monocot hosts. Most of such TFs appear to have putative functions in virulence and pathogenicity. The other regulatory genes found in this category have functions in chromatin remodelling and post transcription regulation, suggesting that the adaptation to dicot hosts also required adaptations at the post-transcriptional and translational level. Confirming this hypothesis, in this category we found several genes involved in translation process/modification, especially at tRNA level (suppl. file 14). This indicating that the chromatin remodeling and the post-translation processes are important for the dicot associated pathogens for host interactions and/or plant cell wall interaction.

Discussion

The ancestral *Colletotrichum* was associated with dicot plants and certain branches progressively adapted to different monocot hosts. The diversification of species inside the genus took place during the Upper (or Late) Cretaceous, 68.76 mya (103.85 – 45.53). This period was characterized by the ecological success of angiosperms that appeared in the fossil records (between 145 and 66 mya) [29]. Previous studies indicate that ancestral angiosperms lived in low evaporative niches during the Early Cretaceous [30] before the period of their quick diversification in the Mid Cretaceous [31]. During the Late Cretaceous, evolving angiosperms spread towards the poles [32] and gained ecological dominance in most of the world’s ecosystems by replacing gymnosperms in the evaporatively more

demanding upper canopy [33]. In our dataset at least three different events of host jumps and specialization to monocots were detected, the first when species belonging to the Graminicola complex diverged from those belonging to the Spaethianum complex around 25.23 mya (42.71 – 14.91), the second when *C. orchidophilum* diverged from the common ancestor of species belonging to the Acutatum complex around 14.58 mya (23.89 – 8.89) and the third event when *C. phormii* diverged from the closely related species *C. salicis* around 3.45 mya (6.55 – 1.82).

All members of the Graminicola complex are pathogenic to species belonging to the Poaceae. However, while most of the species can infect plants belonging to the Panicoideae subfamily (PACMAD clade), *C. zoysiae* is pathogenic to *Zoysia tenuifolia* which belongs to the Chloridoideae subfamily (PACMAD clade) and *C. cereale* is pathogenic to *Poa annua* which belongs to the Pooideae subfamily (BOP clade). The ancestor of all hosts of the Graminicola species can be placed at the crown node of BOP and PACMAD that is dated at 57 mya (75 – 51 mya) in the late Paleogene [18]. This event happened before the differentiation of species belonging to the Graminicola complex and those belonging to the Spaethianum complex while the quick species diversification into Graminicola species took place between the Miocene and the Oligocene, 18.59 mya (32.56 – 10.62 mya) overlapping with the occupation of open habitats in Africa of their hosts that occurred in the late Eocene–early Oligocene. The Oligocene period was considerably drier than the rest of the Tertiary and these factors might have had an effect on the decrease of the forest cover and the expansion of open habitats [34]. The second jump to monocot hosts happened when *C. orchidophilum* diverged from the ancestor in common with species belonging to the Acutatum complex around 14.58 mya (23.89 – 8.89). *C. orchidophilum* is host specific, infecting different species belonging to the Orchidaceae including species belonging to *Phalaenopsis*, *Cycnoches*, *Dendrobium* and *Vanilla*genera [9,10,35] covering the entire diversity of the Orchidaceae. Previous studies reported that the common ancestor of orchids was supposed to have existed much earlier, between 76 and 84 mya [36]. The last of the three monocot specialization events happened when *C. phormii* diverged from the closely related species *C. salicis* in the Neogene, around 3.45 mya (6.55 – 1.82). *C. phormii* is a worldwide-distributed pathogen of *Phormium* spp. *Dianella*-like fossils from the Eocene have been placed at the crown of the genera *Phormium* and *Dianella*, dating the divergence between these two genera to around 45 mya (SD = 1.0) (McLay & Bayly, 2016), which is much earlier than the estimated appearance of *C. phormii*. Among the three events described, *C. orchidophilum* and *C. phormii* might

have acquired a key gene or genes that allow the host jump after the appearance of the host while the ancestor of species belonging to the Graminicola complex have evolved simultaneously with its hosts. Interestingly, all lineages of *Colletotrichum* associated with monocots show a certain level of host specificity which could reflect their more recent host jumps.

Analysis of the plant cell wall degradation related CAZome of the different *Colletotrichum* species did not reveal large differences, especially when compared to similar studies in the genus *Aspergillus* [38,39]. The dicot infecting species have a higher overall number of genes encoding putative plant biomass degrading enzymes than the species with monocot hosts, which confirms results found on a previous study comparing *C. higginsianum* and *C. graminicola* genome [6]. This is also apparent by the number of CAZy families encoding carbohydrate esterases (CE), glycoside hydrolases (GH) or polysaccharide lyases (PL), for which the dicot infecting species have a significantly higher number of genes, even though this difference per family is often small. In contrast, higher gene numbers per family for the monocot infecting species are involved in xylan degradation, a prominent component of monocot cell walls. This difference between the monocot and dicot infecting species reflects the more diverse cell walls of dicots [40], which would require a broader set of enzymes to efficiently degrade them. A clear difference between monocot and dicot infecting species was found in the number of genes encoding putative pectin degrading enzymes. Pectin is a major component of dicot cell walls, but nearly absent in monocots [40]. Studies of specific CAZymes in *Colletotrichum* spp. are relatively few, and they only address some of the enzymes involved in plant biomass degradation [41,42]. Hydrogen peroxide (H_2O_2) may have multiple roles in plant pathogenic fungi because two subfamily AA5_2 alcohol oxidases have been characterized from *C. graminicola* and *C. gloeosporioides* [43,44]. These enzymes have broad substrate ranges and oxidize aliphatic primary alcohols to the corresponding aldehydes, by simultaneously reducing oxygen to hydrogen peroxide. Although their natural substrates have not yet been identified, these enzymes were suggested to have a role in plant cell wall degradation. In addition, an AA5_2 raffinose oxidase that uses trisaccharide raffinose as its preferred substrate, has been characterized from *C. graminicola* [45]. Moreover, a recent study showed that another AA5_2 paralog from *C. graminicola* oxidizes aryl alcohols to the corresponding aldehydes, thus describing aryl alcohol oxidase activity in the CAZy family AA5, which is traditionally related to AA3 glucose methanol choline (GMC) oxidoreductases [46].

Overall, the transcriptome analysis indicates a higher substrate specificity in the monocot pathogenic species *C. graminicola* and *C. phormii* while the response of the dicot pathogens do not seem to discriminate between the different plant substrates. In contrast to the low differences in gene numbers per CAZy family, comparison of the transcriptomes of *C. higginsianum*, *C. nymphaeae*, *C. phormii* and *C. graminicola* revealed high diversity in gene expression. In *Aspergillus*, proteomic comparisons of a large number of species revealed a much higher diversity than was expected based on genome content and differences were more associated with taxonomic distance [47,48]. These results in part match with previous studies of the production of plant biomass degrading enzymes in *Colletotrichum*. *C. graminicola* has been shown to produce β-glucosidase, β-xylosidase and xylanase activity during solid-state fermentation on different plant biomass substrates. Enzyme families containing these activities (GH1, GH3, GH10, GH11, GH43) were also expressed on plant biomass in our study. Studies into the expression of specific genes revealed monomeric inducers of the responsible regulatory systems. An endopolygalacturonase encoding gene of *C. lindemuthianum* was expressed in the presence of L-arabinose and L-rhamnose [49]. Several of the CAZy genes of *Colletotrichum* have been implicated in pathogenicity [50,51]. Transcriptome profiling of *C. graminicola* and *C. higginsianum* has revealed highly dynamic expression of CAZy genes during the infection process. For example, in *C. graminicola* and *C. higginsianum*, significant upregulation of several genes encoding cellulolytic enzymes was observed during the necrotrophic phase compared to the biotrophic phase, during the *in vitro* growth or the formation of the penetration appressorium [3,6]. In *C. higginsianum* and *C. graminicola*, an orthologous GH131 encoding gene was highly upregulated during both biotrophic and necrotrophic phases, whereas in *C. higginsianum*, another GH131 family gene was also upregulated during appressorial penetration and biotrophic phase [52]. In addition, the corresponding recombinant GH131 proteins were demonstrated to have broad specificity towards substrates with β-1,3- and β-1,4-glucosidic linkages, and they were suggested to either breaking down the hemicellulose heteropolymeric structure or facilitating other enzymes to access cellulose [52]. In *C. fructicola*, a transcriptomic study of four types of infection-related structures revealed an upregulated expression of 27 CAZy genes during appressorium formation [53]. Among these genes, 14 encode for redox enzymes with the highest enrichments from AA2 (heme-containing peroxidases) and AA5 (copper radical oxidases, CROs). Under cellophane infectious hyphae, high expression of GH7, AA9, PL1 and CBM1 family members was also detected. As in our

study only a single time point was analyzed, this could explain the absence of the induction of some of these genes in our results. Previous studies have reported gene duplications within the CAZy genes in species characterized by a broad host range [15,16]. Interestingly, different members of the CAZy family GH43 have been identified in three different conditions. Both results suggest that the GH43 may be an important family for plant substrate interaction and/or degradation in *Colletotrichum* species.

The expression of the transcription factors (TFs) and other regulatory genes of *C. higginsianum*, *C. nymphaeae*, *C. phormii* and *C. graminicola* were analyzed based on the orthogroups clustering and according to the different conditions. Unexpectedly, none of the major known TFs involved in plant biomass utilization [2] passed our requirements/cut off, while most of differentially expressed regulatory genes identified were TFs with uncharacterized function or other regulatory factors, mainly involved in chromatin remodelling. We found differentially expressed TFs specific to plant substrates, to monocot pathogenic species and to dicot pathogenic species on both MS and DS. We did not identify differentially expressed TFs specific for the dicot substrate. Exceptions are monocot associated pathogens which overexpressed one TF and one methyltransferase in the monocot substrate, and two TFs and one methyltransferase in both plant substrates. This suggests that adaptation to monocots required changes not only at the transcriptional level, but also at the chromatin access level. However, half of such TFs and other regulatory factors were overexpressed only by dicot pathogens, suggesting that dicot pathogens have a more complex regulation, most likely reflecting the substrate complexity of their host plants. The majority of DE TFs identified in this study do not have a clear function or have a very general role, but our results suggest that, at least some of them, may have a potential role in plant interaction.

Despite millions of years of divergent evolution, gene content among the species is, overall, highly similar, with the main differences being in plant biomass degradation, separating monocot and dicot pathogens. However, a much stronger level of diversity appears to occur at the transcriptional level. This can in part be assigned to the use of non-orthologous members of the same CAZy family by different *Colletotrichum* species. Our results indicate a higher substrate specificity in the monocot pathogenic species *C. graminicola* and *C. phormii* while the response of the dicot pathogenic species seem to be more associated with the general presence of plant substrates.

Materials and Methods

Strains and nucleic acids purification

The genomes of 18 *Colletotrichum* species were sequenced and compared to the genomes of publicly available representative species (Table 3). Total genomic DNA was extracted using modified CTAB methods (Kim *et al.*, 1990; Baek & Kenerley, 1998). Total RNA was extracted from frozen mycelium ground in a Tissue Lyser (QIAGEN) using TRIzol reagent (Invitrogen) according to the manufacturer's instructions. RNA integrity and quantity were analysed on a 1% agarose electrophoresis gel and with the RNA6000 Nano Assay, using the Agilent 2100 Bioanalyzer (Agilent Technologies) [56]. Further details are provided in the supplementary file 15.

Table 3. *Colletotrichum* spp. genomes used in this study.

JGI code	Organisms	complex	Strain	Host	Host Clade	Origin
Colorb1	*Colletotrichum orbiculare*	orbiculare	MAFF 240422	*Cucumis sativus*	dicot	Japan
Gloci1	*Colletotrichum noveboracense*	gloeosorioides	23	unknown	dicot	unknown
Colch1	*Colletotrichum chlorophyti*	none	NTL11	*Solanum lycopersicum*	dicot	Japan
Colhig2	*Colletotrichum higginsianum*	destructivum	IMI 349063	*Brassica rapa*	dicot	Trinidad & Tobago
Colin1	*Colletotrichum incanum*	spaethianum	MAFF 238712	*Raphanus sativus*	dicot	Japan
Colto1	*Colletotrichum tofieldiae*	spaethianum	861	*Arabidopsis thaliana*	dicot	Spain
**Colce1**	***Colletotrichum cereale***	**graminicola**	**CBS 129662**	***Poa annua***	**monocot**	**USA**
**Coler1**	***Colletotrichum eremochloae***	**graminicola**	**CBS 129661**	***Eremochloa ophiuroides***	**monocot**	**USA**
**Colsu1**	***Colletotrichum sublineola***	**graminicola**	**CBS 131301**	***Sorghum bicolor***	**monocot**	**Burkina Fasso**
**Colfa1**	***Colletotrichum falcatum***	**graminicola**	**MAFF 306170**	***Saccharum officinarum***	**monocot**	**Japan**
Colgr1	*Colletotrichum graminicola*	graminicola	M1.001	*Zea mays*	monocot	USA
**Colna1**	***Colletotrichum navitas***	**graminicola**	**CBS 125086**	***Panicum virgatum***	**monocot**	**USA**
**Colca1**	***Colletotrichum caudatum***	**graminicola**	**CBS 131602**	***Sorghastrum nutans***	**monocot**	**USA**
**Colso1**	***Colletotrichum somersetensis***	**graminicola**	**CBS 131599**	***Sorghastrum nutans***	**monocot**	**USA**
**Colzo1**	***Colletotrichum zoysiae***	**graminicola**	**MAFF 235873**	***Zoysia tenuifolia***	**monocot**	**Japan**
Color1	*Colletotrichum orchidophilum*	none	IMI 309357	*Phalaenopsis sp.*	monocot	United Kingdom
Colsa1	*Colletotrichum salicis*	acutatum	CBS 607.94	*Salix sp.*	dicot	Netherlands
**Colph1**	***Colletotrichum phormii***	**acutatum**	**CBS 102054**	***Phormium sp.***	**monocot**	**New Zealand**
**Colgo1**	***Colletotrichum godetiae***	**acutatum**	**CBS 193.32**	***Olea europaea***	**dicot**	**Greece**
Colfi1	*Colletotrichum fioriniae*	acutatum	IMI 504882	*Fragaria x ananassa*	dicot	New Zealand
**Colac2**	***Colletotrichum acutatum s.s.***	**acutatum**	**CBS 112980**	***Pinus radiata***	**dicot**	**South Africa**
**Colab1**	***Colletotrichum abscissum***	**acutatum**	**IMI 504890**	***Citrus x sinensis***	**dicot**	**USA**
**Collu1**	***Colletotrichum lupini***	**acutatum**	**CBS 109225**	***Lupinus albus***	**dicot**	**Ukraine**
**Colta1**	***Colletotrichum tamarilloi***	**acutatum**	**CBS 129955**	***Solanum betaceum***	**dicot**	**Colombia**
**Colco1**	***Colletotrichum costaricense***	**acutatum**	**IMI 309622**	***Coffea sp.***	**dicot**	**Costa Rica**
**Colcu1**	***Colletotrichum cuscutae***	**acutatum**	**IMI 304802**	***Cuscuta sp.***	**dicot**	**Dominica**
**Colpa1**	***Colletotrichum paranaense***	**acutatum**	**IMI 384185**	***Caryocar brasiliense***	**dicot**	**Brazil**
**Colme1**	***Colletotrichum melonis***	**acutatum**	**CBS 134730**	***Malus domestica***	**dicot**	**Brazil**
Colny1	*Colletotrichum nymphaeae*	acutatum	IMI 504889	*Fragaria x ananassa*	dicot	Denmark
Colsi1	*Colletotrichum simmondsii*	acutatum	CBS 122122	*Carica papaya*	dicot	Australia

Species highlighted in bold were sequenced in this work.

Genome sequencing, assembly, and annotation

Selected strains were sequenced using Pacific Biosciences RSII sequencer using Version C4 according to the manufacturer's instructions. The filtered subread data was assembled using Falcon version 0.2.2 (https://github.com/PacificBiosciences/FALCON), improved with finisherSC version 2.0 , and polished with Quiver version smrtanalysis_2.3.0.140936.p5 (https://github.com/PacificBiosciences/GenomicConsensus). Further details are provided in the supplementary file 15.

For the other strains quantified libraries were prepared for sequencing on the Illumina HiSeq sequencing platform utilizing a TruSeq paired-end cluster kit, v4. Sequencing of the flowcell was performed on the Illumina HiSeq2500 sequencer. Raw reads filtered for artifact and process contamination were assembled with Velvet [58] or SPAdes v3.8.2 [59]. BUSCO v5.5.0 [60] (Benchmarking Universal Single-Copy Orthologs) was used to search the selected genomes for 758 fungal orthologous genes (*fungi_odb10.2019-11-20* data set) to assess the completeness of the genome sequences.

The genome sequences were annotated using the JGI annotation pipeline [61] or MAKER2 v2.31.8 annotation pipeline [62] as previously described [15].

Phylogeny and divergence date estimation

A selection of 126 genomes covering the Pezizomycotina plus the genome of *Saccharomyces cerevisiae* as an outgroup were selected from the MycoCosm database (Supplementary 1) and analyzed. The proteomes were clustered with OrthoFinder v0.4 [63] and single copy gene families were aligned with MAFFT 7 [64] and then concatenated. A substitution model and its parameter values were selected using ProtTest 3.4 [65]. A phylogenetic tree was reconstructed using Bayesian MCMC analysis from the concatenated alignment under the WAG + I evolutionary model and the gamma distribution calculated using four rate categories and homogeneous rates across the tree. The calibrated tree was inferred by applying the RelTime method [66,67] to the supplied phylogenetic tree whose branch lengths were calculated using the Ordinary Least Squares method using MEGA X v10.1.7 [68].

The timetree was computed using 5 calibration point [69–75]. Further details are provided in the supplementary file 15.

The Tao method was used to set minimum and maximum time boundaries on nodes for which calibration densities were provided [76]. The evolutionary distances were computed using the Poisson correction method [77] and are in the units of the number of amino acid substitutions per site. Evolutionary analyses were conducted in MEGA X [68].

Annotation of specific gene categories

Proteins that are transported out of the cell and into the extracellular space were identified with SignalP-4.1 [78]. Protein domains were annotated using Pfam [79] and InterPro [80] and mapped to Gene Ontology (GO) terms [81]. CAZymes were annotated using CAZy pipeline [82].

Peptidases were annotated with the MEROPS database, a hierarchical, structure based classification for peptidases, organized into families and clans (https://www.ebi.ac.uk/merops/) [83].

BLASTp [84] and RunIprScan (http://michaelrthon.com/runiprscan/) results were used to manually identify genes encoding enzymes that are signatures of backbone secondary metabolite (SM) genes in the Ascomycota [85]: nonribosomal peptide synthetases (NRPS; IPR010071, IPR006163, IPR001242), polyketide synthases (PKS; IPR013968), DMATS-family aromatic prenyltransferases (IPR017795, Pfam PF11991), and terpene synthases/cyclases (IPR008949).

Transcription factors were identified using BLASTp against NCBI non-redundant protein sequences (nr) database and the Aspergillus Genome Database (AspGD) [86]. P value of 1e-10 was used as cutoff in both cases. NCBI conserved Domains Database(CCD) and EMBL Simple Modular Architecture Research Tool (SMART) (https://smart.embl.de) [87] were used to manually assign putative function(s) to uncharacterized transcription factors.

Cys_6Zn_2 and Cys_2His_2 regulators were also analysed by phylogenetic analyses (NJ) using orthologs of all kingdoms of known regulators involved in plant biomass degradation [2].

Comparative genomics

Ortholog identification and protein cluster analyses

The Markov Cluster algorithm implemented in mcl v14-137 [88] was used for the identification of protein clusters while (Co-)orthologous groups were identified by Proteinortho v5.16b [89].

Identification of expansions and contractions of gene families associated with PS.

Functional categories associated with mono- or dicot pathogenic species were identified using two different statistical analyses.

Disjoint sets calculated as:

Set 1 = monocot pathogens

Set 2 = dicot pathogens

if (Min Set1 > Max Set2) than term is overrepresented in Set1

if (Min Set2 > Max Set1) than term is overrepresented in Set2

Terms enriched based on Fisher's exact test were calculated for each in each genome in the following subset: secretomes, all core proteins, secreted core proteins, all shared proteins, secreted shared proteins, all species-specific proteins, and secreted species-specific proteins. Profiles were compared to identify terms enriched only in monocot or dicot pathogens.

Transcriptomic analyses

A transfer experiment was performed for transcriptomics. 250 mL of complete medium [90] containing 2% D-glucose in 1 L Erlenmeyer flasks was inoculated with 2.5×10^8 fresh spores, harvested from a MEA plate, and incubated in a rotatory shaker at 25°C for 20 h at 140 rpm. The mycelium was harvested by filtration, washed with liquid MM [90] (without carbon source) and 2.5 g mycelium (wet weight) was transferred to 125 mL Erlenmeyer flasks containing 25 mL MM with 1% of maize powder (MS) or sugar beet pulp (DS), and incubated in a rotatory shaker at 25°C and 140 rpm. After pre-culturing and after 96 h of incubation in MS or DS, the mycelium was harvested by vacuum filtration,

dried between tissue paper, directly frozen in liquid nitrogen and stored at −80°C [56]. All experiments were performed in triplicate. Further details are provided in the supplementary file 15.

Identification and analysis of differential gene expression

For transcriptomes, stranded cDNA libraries were generated using the Illumina Truseq Stranded mRNA Library Prep kit. Sequencing was performed using Illumina HiSeq2500 following a 2x100 indexed run recipe. RNA-Seq raw reads were assembled into consensus sequences using either Rnnotator v3.3.2 [91] or Trinity ver. 2.1.1 [92] and used as biological evidence for the gene prediction. Raw reads were filtered and trimmed for quality and contamination. Filtered RNA-Seq reads from each library were aligned to the corresponding reference genome using HISAT version 0.1.4-beta [93]. FeatureCounts [94] was used to generate the raw gene counts using genome annotations. Only primary hits assigned to the reverse strand were included in the raw gene counts (-s 2 -p --primary options). DESeq2 version 1.10.0 [95] was subsequently used to determine which genes were differentially expressed between pairs of conditions. The parameters used to call a gene differentially expressed between conditions were log2FoldChange > 2 and p-value < 0.05. Further details are provided in the supplementary file 15.

Comparative transcriptomics

A custom script *orthoexpress.py* (https://github.com/RiccardoBaroncelli/Orthoexpress) was developed based on Proteinortho v5.16b [89] output to identify groups of genes showing specific expression patterns.

Recent duplications were manually checked. In case of different behavior of paralogs both forms of the (co-)orthologous groups were analyzed independently.

Seven logical conditions (Table 4) were established to identify genes differentially expressed in specific organisms/conditions.

Table 4. Conditions established for the identification of specific differentially expressed genes.

host	dicot pathogenic			monocot pathogenic			monocot pathogenic			dicot pathogenic			
Species	*C. higginsianum*			*C. graminicola*			*C. phormii*			*C. nympaheae*			
Condition	glucose vs DS	glucose vs MS	DS vs MS	glucose vs DS	glucose vs MS	DS vs MS	glucose vs DS	glucose vs MS	DS vs MS	glucose vs DS	glucose vs MS	DS vs MS	Genes overexpressed in presence of:
0	↑	↑		↑	↑		↑	↑		↑	↑		glucose
1	↓	↓		↓	↓		↓	↓		↓	↓		PS
1a	↓	↓	↑	↓	↓		↓	↓		↓	↓	↑	PS and overexpressed in DS in dicot pathogens
1b	↓	↓		↓	↓	↓	↓	↓	↓	↓	↓		PS and overexpressed in MS in monocot pathogens
2		↓	↓		↓	↓		↓	↓		↓	↓	MS
3	↓		↑	↓		↑	↓		↑	↓		↑	DS
4	↓		↑							↓		↑	DS only in dicot pathogens
5					↓	↓		↓	↓				MS only in monocot pathogens
6				↓	↓		↓	↓					PS only in monocot pathogens
7	↓	↓								↓	↓		PS only in dicot pathogens

Arrows pointing up and highlighted in red indicate overexpressed genes while arrows pointing down (highlighted in blue) indicated downregulated genes. PS: plant substrate; MS: monocot; DS: dicot substrate.

Data availability

The genome sequencing data, assembly and annotations are available at DDBJ/EMBL/GenBank (nucleotide accession numbers, BioProject and BioSamples are reported in Suppl2_Data_access.xlsx) and are available at the JGI fungal genome portal MycoCosm [61].

Competing interests

The authors declare no competing interests.

Acknowledgments

This research was supported by funds from the Ministerio de Ciencia Innovación y Universidades of Spain (AGL2015-66362-R) and grants RTI2018-093611-B-I00 and PID2021-125349NB-100 from the Ministerio de Ciencia e Innovación (MCIN) of Spain AEI/10.13039/501100011033 and the European Regional Development Fund (ERDF). The work (proposals: 10.46936/10.25585/60000617 and

10.46936/10.25585/60000725) conducted by the U.S. Department of Energy Joint Genome Institute (https://ror.org/04xm1d337), a DOE Office of Science User Facility, is supported by the Office of Science of the U.S. Department of Energy operated under Contract No. DE-AC02-05CH11231. EB was supported by a grant of the Dutch Technology Foundation STW, Applied Science division of NWO, and the Technology Program of the Ministry of Economic Affairs 016.130.609 to RPdV. The Academy of Finland grant number 308284 to MRM is acknowledged. This study was also carried out within the Agritech National Research Center and received funding from the European Union Next-GenerationEU (PIANO NAZIONALE DI RIPRESA E RESILIENZA (PNRR) – MISSIONE 4 COMPONENTE 2, INVESTIMENTO 1.4 – D.D. 1032 17/06/2022, CN00000022). R.B. was partially supported by the postdoctoral program of USAL (Programme II).

We would like to thank the staff at the Plataforma Andaluza de Bioinformática of the University of Málaga, Spain, for providing computer resources and technical support.

The authors would also like to thank Francis Martin and Rytas Vilgalys for the permission to use the genome of *Glomerella cingulata* 23 (= *Colletotrichum noveboracense* 23); Jon Magnuson for the permission to use the genome of *Sclerophora sanguinea* CBS 100924; Olafur Andresson for the permission to use the genome of *Lobaria pulmonaria* Scotland reference genome; Dave Greenshields for the permission to use the genome of *Penicillium fellutanum* ATCC 48694.

Author contributions

RB, TB, JAC, IVG, RPdV, SAS and MRT planned and designed the research; RB, JFCD, TB, RPdV, SAS and MRT developed and designed the methodology; RB, JFCD, TB, MP, EB, SH, WA, KL, JP, AL, MK, DB, ED and BH performed the experiments and analysed the data; RB, JFCD, TB, RPdV, SAS and MRT wrote the original draft; RB, JFCD, TB, SH, MRM, JAC, RPdV, SAS and MRT review and edit the manuscript; RB, GLF, BH, JAC, RPdV, SAS & MRT contributed to the funding acquisition.

Additional files

Supplementary File S1: Time-calibrated phylogenomic tree of 123 fungal genomes belonging to the Pezizomycotina subdivision; *Saccharomyces cerevisiae* genome was used as outgroup. Bars around each node represent 95% confidence intervals. The timetree was computed using 5 calibration points highlighted with red dots (1, 2 and 3 are fossils and 4 and 5 are estimated constraints); see details in the materials and methods section. Major taxonomic classes and respective crown divergent times are reported in green while the crown of *Colletotrichum* is highlighted in orange. Mya = million years ago.

Supplementary Table S2: Genomes used in this study and relative information. * Tree position refers to the order of the genomes in the phylogenetic tree shown in figure 1.

Supplementary Table S3: Gene Ontology (GO) enrichment analysis. For each genome the number of encoded proteins associated with a specific GO term is reported. Statistical comparison of the gene number differences in each GO terms between monocot and dicot infecting species were compared with the Wilcoxon rank-sum test and for disjoint sets (for further details see "Identification of expansions and contractions of gene families associated with PS" in the material and methods). *"Both" indicates those species capable of infecting dicot and monocot plants.

Supplementary Table S4: InterPro (IPR) enrichment analysis. For each genome the number of encoded proteins associated with a specific IPR term is reported. Statistical comparison of the gene number differences in each IPR terms between monocot and dicot infecting species were compared with the Wilcoxon rank-sum test and for disjoint sets (for further details see "Identification of expansions and contractions of gene families associated with PS" in the material and methods). *"Both" indicates those species capable of infecting dicot and monocot plants.

Supplementary Table S5: Pfam protein families enrichment analysis. For each genome the number of encoded proteins associated with a specific Pfam term is reported. Statistical comparison of the gene number differences in each Pfam terms between monocot and dicot plant infecting species were compared with the Wilcoxon rank-sum test and for disjoint sets (for further details see "Identification of expansions and contractions of gene families associated with PS" in the material and methods). *"Both" indicates those species capable of infecting dicot and monocot plants.

Supplementary Table S6: Carbohydrate-Active enZYmes (CAZy) encoding gene enrichment analysis. For each genome the number of encoded CAZy is reported. Statistical comparison of the gene number

differences in each CAZy families between monocot and dicot infecting species were compared with the Wilcoxon rank-sum test and for disjoint sets (for further details see “Identification of expansions and contractions of gene families associated with PS” in the material and methods). *“Both” indicates those species capable of infecting dicot and monocot plants.

Supplementary Table S7: Comparison of the genome content of 30 *Colletotrichum* species with respect to putative genes involved in plant biomass degradation.

Overall comparison of the species with respect to relevant CAZy families. Statistical comparison of the gene number differences in each CAZy family between monocot and dicot infecting species were compared with the Wilcoxon rank-sum testand for disjoint sets (for further details see “Identification of expansions and contractions of gene families associated with PS” in the material and methods)

MCO = multicopper oxidase, CDH = cellobiose dehydrogenase, GMC = glucose-methanol-choline oxidoreductase, LPMO = lytic polysaccharide monooxygenases, AXE = acetyl xylan esterase, FAE = feruloyl esterase, PME = pectin methyl esterase, RGAE = rhamnogalacturonan acetyl esterase, GE = glucuronoyl esterase, HAE = hemicellulose acetyl esterase, BGL = β-glucosidase, MND = β-mannosidase, LAC = β-galactosidase, GUS = β-glucuronidase, BXL = β-xylosidase, EGL = endoglucanase, MAN = endomannanase, CBH = cellobiohydrolase, XLN = endoxylanase, XEG = xyloglucanase, AMY = α-amylase, AGD = α-glucosidase, GLA = glucoamylase, AGL = α-galactosidase, PGA = endopolygalacturonase, PGX = exopolygalacturonase, RHG = endorhamnogalacturonase, RGX = exorhamnogalacturonase, XGH = xylogalacturonase, AFC = α-fucosidase, XBH = xylobiohydrolase, AXL = α-xylosidase, INV = invertase, INU = endoinulinase, INX = exoinulinase, ABF = α-arabinofuranosidase, ABN = endoarabinanase, GAL = endogalactanase, AXH = arabinoxylan arabinofuranohydrolase,

AGU = α-glucuronidase, RHA = α-rhamnosidase, UGH = unsaturated galacturonan hydrolase, ABX = exoarabinanase, URGH = unsaturated rhamnogalacturonan hydrolase, AMG = amylo-α-1,6-glucosidase, PLY = pectate lyase, PEL = pectin lyase, RGL = rhamnogalacturonan lyase. *“Both” indicates those species capable of infecting dicot and monocot plants.

Supplementary Table S8: Comparison of the gene content of 30 *Colletotrichum* species with respect to putative peptidases and their inhibitors. *“Both” indicates those species capable of infecting dicot and monocot plants.

Supplementary Table S9: Comparison of the gene content of 30 *Colletotrichum* species with respect to putative transporters. *"Both" indicates those species capable of infecting dicot and monocot plants.

Supplementary Table S10: Comparison of the gene content of 30 *Colletotrichum* species with respect to putative transcription factors. Statistical comparison of the gene number differences in each transcription factors families terms between monocot and dicot plant infecting species were compared with the Wilcoxon rank-sum test. *"Both" indicates those species capable of infecting dicot and monocot plants.

Supplementary Figures S11: Phylogenetic tree of selected gene families based on InterPro (IPR) domain distribution: PL-6 family - IPR039513; Transcription initiation factor IID, subunit 13 - IPR003195; Aconitase, mitochondrial-like - IPR006248; PoSI-like peptidase domain - IPR034187. Red taxa indicate dicot pathogenic species, blue indicate monocot pathogenic species and purple taxa indicate *Colletotrichum* species that can infect both plant hosts. Pink boxes indicate gene lineages specific of the dicot pathogens. Number next to the nodes represent support values expressed as % while thicker branches indicate a support value of 100%.

Supplementary Figure S12: Correlation matrix of 9 RNA-seq libraries. Pairwise Pearson correlation coefficients (PCC) were calculated for comparison among transcriptomes of various combinations of *Colletotrichum* spp. and substrates. Samples were hierarchically clustered with the Euclidean distance method. The color scale indicates the degree of correlation.

Supplementary Figure S13: Volcano plots showing for each pairwise comparison analyzed the genes considered differentially expressed (green dots) based on log2FoldChange > 2 and p-value < 0.05

Supplementary Table S14: List of orthogroups and main biological functions related to the genes identified based on specific expression patterns in each condition established. Information such as: foldchange (positive values indicating overexpressed genes are highlighted in red while negative values indicating down regulated genes are highlighted in blue), conserved domains, gene families and locus tags are also reported.

Supplementary File S15: Extended version of material ad methods used.

REFERENCES

1. Vries RP de, Visser J. *Aspergillus* enzymes involved in degradation of plant cell wall polysaccharides. *Microbiol Mol Biol Rev*. 2001; doi: 10.1128/MMBR.65.4.497-522.2001.

2. Benocci T, Aguilar-Pontes MV, Zhou M, Seiboth B, de Vries RP. Regulators of plant biomass degradation in ascomycetous fungi. *Biotechnol Biofuels*. 2017; doi: 10.1186/s13068-017-0841-x.

3. Molina A, Miedes E, Bacete L, Rodríguez T, Mélida H, Denancé N, et al. *Arabidopsis* cell wall composition determines disease resistance specificity and fitness. *Proc Natl Acad Sci*. National Academy of Sciences; 2021; doi: 10.1073/pnas.2010243118.

4. Sarkar P, Bosneaga E, Auer M. Plant cell walls throughout evolution: towards a molecular understanding of their design principles. *J Exp Bot*. 2009; doi: 10.1093/jxb/erp245.

5. Juge N. Plant protein inhibitors of cell wall degrading enzymes. *Trends Plant Sci*. 2006; doi: 10.1016/j.tplants.2006.05.006.

6. O'Connell RJ, Thon MR, Hacquard S, Amyotte SG, Kleemann J, Torres MF, et al. Lifestyle transitions in plant pathogenic *Colletotrichum* fungi deciphered by genome and transcriptome analyses. *Nat Genet*. 2012; doi: 10.1038/ng.2372.

7. Cuomo CA, Güldener U, Xu J-R, Trail F, Turgeon BG, Pietro AD, et al. The *Fusarium graminearum* genome reveals a link between localized polymorphism and pathogen specialization. *Science*. 2007; doi: 10.1126/science.1143708.

8. King BC, Waxman KD, Nenni NV, Walker LP, Bergstrom GC, Gibson DM, et al. Arsenal of plant cell wall degrading enzymes reflects host preference among plant pathogenic fungi. *Biotechnol Biofuels*. 42011;

9. Baroncelli R, Talhinhas P, Pensec F, Sukno SA, Le Floch G, Thon MR. The *Colletotrichum acutatum* species complex as a model system to study evolution and host specialization in plant pathogens. *Front Microbiol*. 2017; doi: 10.3389/fmicb.2017.02001.

10. Damm U, Cannon PF, Woudenberg JHC, Crous PW. The *Colletotrichum acutatum* species complex. *Stud Mycol*. 2012; doi: 10.3114/sim0010.

11. Baroncelli R, Sukno SA, Sarrocco S, Cafà G, Le Floch G, Thon MR. Whole-genome sequence of the orchid anthracnose pathogen *Colletotrichum orchidophilum*. *Mol Plant Microbe Interact*. 2018; doi: 10.1094/MPMI-03-18-0055-A.

12. Talhinhas P, Baroncelli R. *Colletotrichum* species and complexes: geographic distribution, host range and conservation status. *Fungal Divers*. 2021; doi: 10.1007/s13225-021-00491-9.

13. Haridas S, Albert R, Binder M, Bloem J, LaButti K, Salamov A, et al. 101 *Dothideomycetes* genomes: a test case for predicting lifestyles and emergence of pathogens. *Stud Mycol*. 2020; doi: 10.1016/j.simyco.2020.01.003.

14. Dean RA, Talbot NJ, Ebbole DJ, Farman ML, Mitchell TK, Orbach MJ, et al. The genome sequence of the rice blast fungus *Magnaporthe grisea*. *Nature*. 434:980–62005;

15. Baroncelli R, Amby DB, Zapparata A, Sarrocco S, Vannacci G, Le Floch G, et al. Gene family expansions and contractions are associated with host range in plant pathogens of the genus *Colletotrichum*. *BMC Genomics*. 2016; doi: 10.1186/s12864-016-2917-6.

16. Gan P, Narusaka M, Kumakura N, Tsushima A, Takano Y, Narusaka Y, et al. Genus-wide comparative genome analyses of *Colletotrichum* species reveal specific gene family losses and gains during adaptation to specific infection lifestyles. *Genome Biol Evol*. 2016; doi: 10.1093/gbe/evw089.

17. Gan P, Ikeda K, Irieda H, Narusaka M, O'Connell RJ, Narusaka Y, et al. Comparative genomic and transcriptomic analyses reveal the hemibiotrophic stage shift of *Colletotrichum* fungi. *New Phytol*. 2013; doi: 10.1111/nph.12085.

18. Bouchenak-Khelladi Y, Verboom GA, Savolainen V, Hodkinson TR. Biogeography of the grasses (Poaceae): a phylogenetic approach to reveal evolutionary history in geographical space and geological time. *Bot J Linn Soc*. Oxford Academic; 2010; doi: 10.1111/j.1095-8339.2010.01041.x.

19. Lin SY, Okuda S, Ikeda K, Okuno T, Takano Y. *LAC2* encoding a secreted laccase is involved in appressorial melanization and conidial pigmentation in *Colletotrichum orbiculare*. *Mol Plant-Microbe Interactions®*. Scientific Societies; 2012; doi: 10.1094/MPMI-05-12-0131-R.

20. Fungal Growth. https://www.fung-growth.org/ Accessed 2022 Jan 22.

21. Garrigues S, Kun RS, Peng M, Bauer D, Keymanesh K, Lipzen A, et al. Unraveling the regulation of sugar beet pulp utilization in the industrially relevant fungus *Aspergillus niger*. *iScience*. 2022; doi: 10.1016/j.isci.2022.104065.

22. Couture G, Vo T-TT, Castillo JJ, Mills DA, German JB, Maverakis E, et al. Glycomic Mapping of the Maize Plant Points to Greater Utilization of the Entire Plant. *ACS Food Sci Technol*. American Chemical Society; 2021; doi: 10.1021/acsfoodscitech.1c00318.

23. Finkenstadt VL. A Review on the Complete Utilization of the Sugarbeet. *Sugar Tech*. 2014; doi: 10.1007/s12355-013-0285-y.

24. Câmara-Salim I, Conde P, Feijoo G, Moreira MT. The use of maize stover and sugar beet pulp as feedstocks in industrial fermentation plants – An economic and environmental perspective. *Clean Environ Syst*. 2021; doi: 10.1016/j.cesys.2020.100005.

25. Hood EE, Teoh K (Thomas), Devaiah SP, Vicuna Requesens D. Biomassbiomass Crops for Biofuels and Bio-based Products. In: Christou P, Savin R, Costa-Pierce BA, Misztal I, Whitelaw CBA, editors. *Sustain Food Prod*. New York, NY: Springer;

26. Chroumpi T, Peng M, Markillie LM, Mitchell HD, Nicora CD, Hutchinson CM, et al. Re-routing of Sugar Catabolism Provides a Better Insight Into Fungal Flexibility in Using Plant Biomass-Derived Monomers as Substrates. *Front Bioeng Biotechnol*. 92021;

27. Patyshakuliyeva A, Falkoski DL, Wiebenga A, Timmermans K, de Vries RP. Macroalgae Derived Fungi Have High Abilities to Degrade Algal Polymers. *Microorganisms*. Multidisciplinary Digital Publishing Institute; 2020; doi: 10.3390/microorganisms8010052.

28. Benoit I, Zhou M, Vivas Duarte A, Downes DJ, Todd RB, Kloezen W, et al. Spatial differentiation of gene expression in Aspergillus niger colony grown for sugar beet pulp utilization. *Sci Rep*. Nature Publishing Group; 2015; doi: 10.1038/srep13592.

29. de Boer HJ, Eppinga MB, Wassen MJ, Dekker SC. A critical transition in leaf evolution facilitated the Cretaceous angiosperm revolution. *Nat Commun*. Nature Publishing Group; 2012; doi: 10.1038/ncomms2217.

30. Feild TS, Arens NC, Doyle JA, Dawson TE, Donoghue MJ. Dark and disturbed: a new image of early angiosperm ecology. *Paleobiology*. The Paleontological Society; 2004; doi: 10.1666/0094-8373(2004)030<0082:DADANI>2.0.CO;2.

31. Lidgard S, Crane PR. Quantitative analyses of the early angiosperm radiation. *Nature*. Nature Publishing Group; 1988; doi: 10.1038/331344a0.

32. Crane PR, Lidgard S. Angiosperm diversification and paleolatitudinal gradients in cretaceous floristic diversity. *Science*. American Association for the Advancement of Science; 246:675–81989;

33. Bond WJ. The tortoise and the hare: ecology of angiosperm dominance and gymnosperm persistence. *Biol J Linn Soc*. 1989; doi: 10.1111/j.1095-8312.1989.tb00492.x.

34. Janis CM. Tertiary mammal evolution in the context of changing climates, vegetation, and tectonic events. *Annu Rev Ecol Syst*. 1993; doi: 10.1146/annurev.es.24.110193.002343.

35. Charron C, Hubert J, Minatchy J, Wilson V, Chrysot F, Gerville S, et al. Characterization of *Colletotrichum orchidophilum*, the agent of black spot disease of vanilla. *J Phytopathol*. John Wiley & Sons, Ltd; 2018; doi: 10.1111/jph.12714.

36. Ramírez SR, Gravendeel B, Singer RB, Marshall CR, Pierce NE. Dating the origin of the Orchidaceae from a fossil orchid with its pollinator. *Nature*. Nature Publishing Group; 2007; doi: 10.1038/nature06039.

37. McLAY TGB, Bayly MJ. A new family placement for Australian blue squill, *Chamaescilla*: Xanthorrhoeaceae (Hemerocallidoideae), not Asparagaceae. *Phytotaxa*. 2016; doi: 10.11646/phytotaxa.275.2.2.

38. Kjærbølling I, Vesth T, Frisvad JC, Nybo JL, Theobald S, Kildgaard S, et al. A comparative genomics study of 23 *Aspergillus* species from section Flavi. *Nat Commun*. 2020; doi: 10.1038/s41467-019-14051-y.

39. Vesth TC, Nybo JL, Theobald S, Frisvad JC, Larsen TO, Nielsen KF, et al. Investigation of inter- and intraspecies variation through genome sequencing of *Aspergillus* section Nigri. *Nat Genet*. Nature Publishing Group; 2018; doi: 10.1038/s41588-018-0246-1.

40. Shtein I, Bar-On B, Popper ZA. Plant and algal structure: from cell walls to biomechanical function. *Physiol Plant*. 2018; doi: 10.1111/ppl.12727.

41. Bonivento D, Pontiggia D, Matteo AD, Fernandez-Recio J, Salvi G, Tsernoglou D, et al. Crystal structure of the endopolygalacturonase from the phytopathogenic fungus *Colletotrichum lupini* and its interaction with polygalacturonase-inhibiting proteins. *Proteins Struct Funct Bioinforma*. John Wiley & Sons, Ltd; 2008; doi: 10.1002/prot.21610.

42. Gregori R, Mari M, Bertolini P, Barajas JAS, Tian JB, Labavitch JM. Reduction of *Colletotrichum acutatum* infection by a polygalacturonase inhibitor protein extracted from apple. *Postharvest Biol Technol*. 2008; doi: 10.1016/j.postharvbio.2007.10.006.

43. Yin D (Tyler), Urresti S, Lafond M, Johnston EM, Derikvand F, Ciano L, et al. Structure–function characterization reveals new catalytic diversity in the galactose oxidase and glyoxal oxidase family. *Nat Commun*. Nature Publishing Group; 2015; doi: 10.1038/ncomms10197.

44. Ribeaucourt D, Saker S, Navarro D, Bissaro B, Drula E, Correia LO, et al. Identification of copper-containing oxidoreductases in the secretomes of three *Colletotrichum* species with a focus on copper radical oxidases for the biocatalytic production of fatty aldehydes. *Appl Environ Microbiol*. 2021; doi: 10.1128/AEM.01526-21.

45. Andberg M, Mollerup F, Parikka K, Koutaniemi S, Boer H, Juvonen M, et al. A novel *Colletotrichum graminicola* raffinose oxidase in the AA5 family. *Appl Environ Microbiol*. American Society for Microbiology; 2017; doi: 10.1128/AEM.01383-17.

46. Mathieu Y, Offen WA, Forget SM, Ciano L, Viborg AH, Blagova E, et al. Discovery of a fungal copper radical oxidase with high catalytic efficiency toward 5-hydroxymethylfurfural and benzyl alcohols for bioprocessing. *ACS Catal*. American Chemical Society; 2020; doi: 10.1021/acscatal.9b04727.

47. Mäkelä MR, DiFalco M, McDonnell E, Nguyen TTM, Wiebenga A, Hildén K, et al. Genomic and exoproteomic diversity in plant biomass degradation approaches among Aspergilli. *Stud Mycol*. 2018; doi: 10.1016/j.simyco.2018.09.001.

48. de Vries RP, Riley R, Wiebenga A, Aguilar-Osorio G, Amillis S, Uchima CA, et al. Comparative genomics reveals high biological diversity and specific adaptations in the industrially and medically important fungal genus *Aspergillus*. *Genome Biol*. 2017; doi: 10.1186/s13059-017-1151-0.

49. Hugouvieux V, Centis S, Lafitte C, Esquerre-Tugaye M. Induction by (alpha)-L-arabinose and (alpha)-L-rhamnose of endopolygalacturonase gene expression in *Colletotrichum lindemuthianum*. *Appl Environ Microbiol*. 63:2287–921997;

50. Yakoby N, Beno-Moualem D, Keen NT, Dinoor A, Pines O, Prusky D. *Colletotrichum gloeosporioides* pelB is an important virulence factor in avocado fruit-fungus interaction. *Mol Plant-Microbe Interactions®*. Scientific Societies; 2001; doi: 10.1094/MPMI.2001.14.8.988.

51. Herbert C, O'Connell R, Gaulin E, Salesses V, Esquerré-Tugayé M-T, Dumas B. Production of a cell wall-associated endopolygalacturonase by *Colletotrichum lindemuthianum* and pectin degradation during bean infection. *Fungal Genet Biol*. 2004; doi: 10.1016/j.fgb.2003.09.008.

52. Anasontzis GE, Lebrun M-H, Haon M, Champion C, Kohler A, Lenfant N, et al. Broad-specificity GH131 β-glucanases are a hallmark of fungi and oomycetes that colonize plants. *Environ Microbiol*. 2019; doi: 10.1111/1462-2920.14596.

53. Liang X, Shang S, Dong Q, Wang B, Zhang R, Gleason ML, et al. Transcriptomic analysis reveals candidate genes regulating development and host interactions of *Colletotrichum fructicola*. *BMC Genomics*. 2018; doi: 10.1186/s12864-018-4934-0.

54. Kim WK, Mauthe W, Hausner G, Klassen GR. Isolation of high molecular weight DNA and double-stranded RNAs from fungi. *Can J Bot*. 1990; doi: 10.1139/b90-249.

55. Baek J-M, Kenerley CM. The arg2 Gene of *Trichoderma virens*: cloning and development of a homologous transformation system. *Fungal Genet Biol*. 1998; doi: 10.1006/fgbi.1997.1025.

56. Klaubauf S, Zhou M, Lebrun M-H, de Vries RP, Battaglia E. A novel L-arabinose-responsive regulator discovered in the rice-blast fungus *Pyricularia oryzae* (*Magnaporthe oryzae*). *FEBS Lett*. 2016; doi: 10.1002/1873-3468.12070.

57. Lam K-K, LaButti K, Khalak A, Tse D. FinisherSC: a repeat-aware tool for upgrading *de novo* assembly using long reads. *Bioinformatics*. 2015; doi: 10.1093/bioinformatics/btv280.

58. Zerbino DR, Birney E. Velvet: Algorithms for de novo short read assembly using de Bruijn graphs. *Genome Res*. 2008; doi: 10.1101/gr.074492.107.

59. Bankevich A, Nurk S, Antipov D, Gurevich AA, Dvorkin M, Kulikov AS, et al. SPAdes: a new genome assembly algorithm and its applications to single-cell sequencing. *J Comput Biol*. 2012; doi: 10.1089/cmb.2012.0021.

60. Waterhouse RM, Seppey M, Simão FA, Manni M, Ioannidis P, Klioutchnikov G, et al. BUSCO applications from quality assessments to gene prediction and phylogenomics. *Mol Biol Evol*. 2018; doi: 10.1093/molbev/msx319.

61. Grigoriev IV, Nikitin R, Haridas S, Kuo A, Ohm R, Otillar R, et al. MycoCosm portal: gearing up for 1000 fungal genomes. *Nucleic Acids Res*. 2014; doi: 10.1093/nar/gkt1183.

62. Holt C, Yandell M. MAKER2: an annotation pipeline and genome-database management tool for second-generation genome projects. *BMC Bioinformatics*. 2011; doi: 10.1186/1471-2105-12-491.

63. Emms DM, Kelly S. OrthoFinder: solving fundamental biases in whole genome comparisons dramatically improves orthogroup inference accuracy. *Genome Biol*. 2015; doi: 10.1186/s13059-015-0721-2.

64. Katoh K, Standley DM. MAFFT multiple sequence alignment software version 7: improvements in performance and usability. *Mol Biol Evol*. 2013; doi: 10.1093/molbev/mst010.

65. Abascal F, Zardoya R, Posada D. ProtTest: selection of best-fit models of protein evolution. *Bioinformatics*. 2005; doi: 10.1093/bioinformatics/bti263.

66. Tamura K, Battistuzzi FU, Billing-Ross P, Murillo O, Filipski A, Kumar S. Estimating divergence times in large molecular phylogenies. *Proc Natl Acad Sci U S A*. 2012; doi: 10.1073/pnas.1213199109.

67. Tamura K, Tao Q, Kumar S. Theoretical foundation of the RelTime method for estimating divergence times from variable Eevolutionary rates. *Mol Biol Evol*. Oxford Academic; 2018; doi: 10.1093/molbev/msy044.

68. Kumar S, Stecher G, Li M, Knyaz C, Tamura K. MEGA X: molecular evolutionary genetics analysis across computing platforms. *Mol Biol Evol*. Oxford Academic; 2018; doi: 10.1093/molbev/msy096.

69. Taylor TN, Hass H, Kerp H. The oldest fossil ascomycetes. *Nature*. 1999; doi: 10.1038/21349.

70. Taylor TN, Hass H, Kerp H, Krings M, Hanlin RT. Perithecial ascomycetes from the 400 million year old Rhynie chert: an example of ancestral polymorphism. *Mycologia*. 97:269–852005;

71. Dörfelt H, Schmidt AR. A fossil *Aspergillus* from Baltic amber. *Mycol Res*. 109:956–602005;

72. Sung G-H, Poinar GO, Spatafora JW. The oldest fossil evidence of animal parasitism by fungi supports a Cretaceous diversification of fungal–arthropod symbioses. *Mol Phylogenet Evol*. 2008; doi: 10.1016/j.ympev.2008.08.028.

73. Lücking R, Huhndorf S, Pfister DH, Plata ER, Lumbsch HT. Fungi evolved right on track. *Mycologia*. Taylor & Francis; 2009; doi: 10.3852/09-016.

74. Schmidt AR, Beimforde C, Seyfullah LJ, Wege S-E, Dörfelt H, Girard V, et al. Amber fossils of sooty moulds. *Rev Palaeobot Palynol*. 2014; doi: 10.1016/j.revpalbo.2013.07.002.

75. Beimforde C, Feldberg K, Nylinder S, Rikkinen J, Tuovila H, Dörfelt H, et al. Estimating the phanerozoic history of the Ascomycota lineages: combining fossil and molecular data. *Mol Phylogenet Evol*. 2014; doi: 10.1016/j.ympev.2014.04.024.

76. Tao Q, Tamura K, Mello B, Kumar S. Reliable confidence intervals for RelTime estimates of evolutionary divergence times. *Mol Biol Evol*. Oxford Academic; 2020; doi: 10.1093/molbev/msz236.

77. Zuckerkandl E, Pauling L. Evolutionary divergence and convergence in proteins. In: Bryson V, Vogel HJ, editors. *Evol Genes Proteins*. Academic Press;

78. Petersen TN, Brunak S, von Heijne G, Nielsen H. SignalP 4.0: discriminating signal peptides from transmembrane regions. *Nat Methods*. 2011; doi: 10.1038/nmeth.1701.

79. Sonnhammer EL, Eddy SR, Durbin R. Pfam: a comprehensive database of protein domain families based on seed alignments. *Proteins*. 1997; doi: 10.1002/(sici)1097-0134(199707)28:3<405::aid-prot10>3.0.co;2-l.

80. Apweiler R, Attwood TK, Bairoch A, Bateman A, Birney E, Biswas M, et al. The InterPro database, an integrated documentation resource for protein families, domains and functional sites. *Nucleic Acids Res*. 2001; doi: 10.1093/nar/29.1.37.

81. Ashburner M, Ball CA, Blake JA, Botstein D, Butler H, Cherry JM, et al. Gene Ontology: tool for the unification of biology. *Nat Genet*. Nature Publishing Group; 2000; doi: 10.1038/75556.

82. Lombard V, Golaconda Ramulu H, Drula E, Coutinho PM, Henrissat B. The carbohydrate-active enzymes database (CAZy) in 2013. *Nucleic Acids Res*. Oxford Academic; 2014; doi: 10.1093/nar/gkt1178.

83. Rawlings ND, Barrett AJ, Bateman A. MEROPS: the database of proteolytic enzymes, their substrates and inhibitors. *Nucleic Acids Res*. 2012; doi: 10.1093/nar/gkr987.

84. Altschul SF, Gish W, Miller W, Myers EW, Lipman DJ. Basic local alignment search tool. *J Mol Biol*. 1990; doi: 10.1016/S0022-2836(05)80360-2.

85. Schardl CL, Young CA, Hesse U, Amyotte SG, Andreeva K, Calie PJ, et al. Plant-symbiotic fungi as chemical engineers: multi-genome analysis of the Clavicipitaceae reveals dynamics of alkaloid loci. Heitman J, editor. *PLoS Genet*. 2013; doi: 10.1371/journal.pgen.1003323.

86. Cerqueira GC, Arnaud MB, Inglis DO, Skrzypek MS, Binkley G, Simison M, et al. The *Aspergillus* Genome Database: multispecies curation and incorporation of RNA-Seq data to improve structural gene annotations. *Nucleic Acids Res*. Oxford Academic; 2014; doi: 10.1093/nar/gkt1029.

87. Letunic I, Bork P. 20 years of the SMART protein domain annotation resource. *Nucleic Acids Res*. 2018; doi: 10.1093/nar/gkx922.

88. Enright AJ, Van Dongen S, Ouzounis CA. An efficient algorithm for large-scale detection of protein families. *Nucleic Acids Res*. 2002; doi: 10.1093/nar/30.7.1575.

89. Lechner M, Findeiß S, Steiner L, Marz M, Stadler PF, Prohaska SJ. Proteinortho: Detection of (Co-)orthologs in large-scale analysis. *BMC Bioinformatics*. 2011; doi: 10.1186/1471-2105-12-124.

90. Vries RP de, Burgers K, Vondervoort PJI van de, Frisvad JC, Samson RA, Visser J. A new black *Aspergillus* species, *A. vadensis*, is a promising host for homologous and heterologous protein production. *Appl Environ Microbiol*. American Society for Microbiology; 2004; doi: 10.1128/AEM.70.7.3954-3959.2004.

91. Martin J, Bruno VM, Fang Z, Meng X, Blow M, Zhang T, et al. Rnnotator: an automated *de novo* transcriptome assembly pipeline from stranded RNA-Seq reads. *BMC Genomics*. 2010; doi: 10.1186/1471-2164-11-663.

92. Grabherr MG, Haas BJ, Yassour M, Levin JZ, Thompson DA, Amit I, et al. Full-length transcriptome assembly from RNA-Seq data without a reference genome. *Nat Biotechnol*. Nature Publishing Group; 2011; doi: 10.1038/nbt.1883.

93. Kim D, Langmead B, Salzberg SL. HISAT: a fast spliced aligner with low memory requirements. *Nat Methods*. 2015; doi: 10.1038/nmeth.3317.

94. Liao Y, Smyth GK, Shi W. featureCounts: an efficient general purpose program for assigning sequence reads to genomic features. *Bioinformatics*. 2014; doi: 10.1093/bioinformatics/btt656.

95. Love MI, Huber W, Anders S. Moderated estimation of fold change and dispersion for RNA-seq data with DESeq2. *Genome Biol*. 2014; doi: 10.1186/s13059-014-0550-8.

Click here to access/download;Table;Table1.xlsx

Table 1. Description of transcription profiles, number of genes identified and main biological functions in each condition.

Condition	Conditions of overexpression	# genes	Main biological function / description
0	in the presence of glucose	10	primary metabolism; transporters
1	in the presence of PS	13	Cazy GH27 / GH5 / GH43; transporters; 2 transcription factors
1a	in the presence of PS and overexpressed in DS in eudicot pathogens	1	Cazy GH43
1b	in the presence of PS and overexpressed in MS in monocot pathogens	2	Sugar transport; alkaline phosphatases
2	in the presence of MS	0	NA
3	in the presence of DS	0	NA
4	in the presence of DS only in dicot pathogens	2	Cazy GH142; transmembrane protein
5	in the presence of MS only in monocot pathogens	10	Cazy GH11(CBM1); transmembrane proteins; 2 transcription factor
6	in the presence of PS only in monocot pathogens	22	Cazy GH43 /GH62(CBM1); transporters, oxidoreductase activity; 3 transcription factors
7	in the presence of PS only in dicot pathogens	52	Unknown functions, Zinc finger – nucleic acid binding; 6 transcription factors

PS: plant substrate; MS: monocot substrate; DS: dicot substrate

Click here to access/download;Table;Table2.xlsx

Table 2. Transcription factors and other genes involved in modulating gene expression identifi

Condition	Conditions of overexpression	Orthogroup	Domain
1	in presence of PS	OG_1905	Cys6Zn2 TF
		OG_7409	Cys6Zn2 TF
5	in presence of MS only in monocot pathogens	OG_8644	Methyltransferase
		OG_1140	Cys2His2 TF
6	in presence of PS only in monocot pathogens	OG_6982	Methyltransferase
		OG_401	Cys6Zn2 TF
		OG_1148	Cys6Zn2 TF
7	in presence of PS only in dicot pathogens	OG_2149	Cys6Zn2 TF
		OG_2547	SFN2 helicase
		OG_3693	Cys6Zn2 TF
		OG_2666	Cys6Zn2 TF
		OG_2742	GATA-like TF
		OG_5209	E3 Ubiquitin ligase
		OG_7815	bZIP TF
		OG_8935	GATA TF
		OG_94	E3 Ubiquitin ligase

PS: plant substrate; MS: monocot substrate; DS: dicot substrate

ed in the transcriptome dataset.

Predicted/putative function
Unknown/vegetative asexual development
Activator of stress 1 (ASG1)/hyphal growth
Secondary metabolism
Unknown
Unknown/putative growth control
Activator of purine utilization
Secondary metabolism
Conidiophore development, hyphal growth
Chromatin remodeling/DNA repair
Unknown
Cutinase transcription factor 1 (CTF1)
Development and disease
Proteasome-mediated ubiquitin-dependent protein catabolic process
Oxidative stress/pathogenicity
Sensing
Ubiquitin ligase/histone regulation

Click here to access/download;Table;Table3.xlsx

Table 3. *Colletotrichum* genomes used analized in this study and relative informations

JGI code	Organisms	complex	Strain	Host
Colorb1	*Colletotrichum orbiculare*	orbiculare	MAFF 240422	*Cucumis sativus*
Gloci1	*Colletotrichum noveboracense*	gloeosorioides	23	unknown
Colch1	*Colletotrichum chlorophyti*	none	NTL11	*Solanum lycopersicum*
Colhig2	*Colletotrichum higginsianum*	destructivum	IMI 349063	*Brassica rapa*
Colin1	*Colletotrichum incanum*	spaethianum	MAFF 238712	*Raphanus sativus*
Colto1	*Colletotrichum tofieldiae*	spaethianum	861	*Arabidopsis thaliana*
**Colce1**	***Colletotrichum cereale***	**graminicola**	**CBS 129662**	***Poa annua***
**Coler1**	***Colletotrichum eremochloae***	**graminicola**	**CBS 129661**	***Eremochloa ophiuroides***
**Colsu1**	***Colletotrichum sublineola***	**graminicola**	**CBS 131301**	***Sorghum bicolor***
**Colfa1**	***Colletotrichum falcatum***	**graminicola**	**MAFF 306170**	***Saccharum officinarum***
Colgr1	*Colletotrichum graminicola*	graminicola	M1.001	*Zea mays*
**Colna1**	***Colletotrichum navitas***	**graminicola**	**CBS 125086**	***Panicum virgatum***
**Colca1**	***Colletotrichum caudatum***	**graminicola**	**CBS 131602**	***Sorghastrum nutans***
**Colso1**	***Colletotrichum somersetensis***	**graminicola**	**CBS 131599**	***Sorghastrum nutans***
**Colzo1**	***Colletotrichum zoysiae***	**graminicola**	**MAFF 235873**	***Zoysia tenuifolia***
Color1	*Colletotrichum orchidophilum*	none	IMI 309357	*Phalaenopsis sp.*
Colsa1	*Colletotrichum salicis*	acutatum	CBS 607.94	*Salix sp.*
**Colph1**	***Colletotrichum phormii***	**acutatum**	**CBS 102054**	***Phormium sp.***
**Colgo1**	***Colletotrichum godetiae***	**acutatum**	**CBS 193.32**	***Olea europaea***
Colfi1	*Colletotrichum fioriniae*	acutatum	IMI 504882	*Fragaria x ananassa*
**Colac2**	***Colletotrichum acutatum s.s.***	**acutatum**	**CBS 112980**	***Pinus radiata***
**Colab1**	***Colletotrichum abscissum***	**acutatum**	**IMI 504890**	***Citrus x sinensis***
**Collu1**	***Colletotrichum lupini***	**acutatum**	**CBS 109225**	***Lupinus albus***
**Colta1**	***Colletotrichum tamarilloi***	**acutatum**	**CBS 129955**	***Solanum betaceum***
**Colco1**	***Colletotrichum costaricense***	**acutatum**	**IMI 309622**	***Coffea sp.***
**Colcu1**	***Colletotrichum cuscutae***	**acutatum**	**IMI 304802**	***Cuscuta sp.***
**Colpa1**	***Colletotrichum paranaense***	**acutatum**	**IMI 384185**	***Caryocar brasiliense***
**Colme1**	***Colletotrichum melonis***	**acutatum**	**CBS 134730**	***Malus domestica***
Colny1	*Colletotrichum nymphaeae*	acutatum	IMI 504889	*Fragaria x ananassa*
Colsi1	*Colletotrichum simmondsii*	acutatum	CBS 122122	*Carica papaya*

In bold genomes produced in this work

Host Clade	Origin
dicot	Japan
dicot	unknown
dicot	Japan
dicot	Trinidad & Tobago
dicot	Japan
dicot	Spain
**monocot**	**USA**
**monocot**	**USA**
**monocot**	**Burkina Fasso**
**monocot**	**Japan**
monocot	USA
**monocot**	**USA**
**monocot**	**USA**
**monocot**	**USA**
**monocot**	**Japan**
monocot	United Kingdom
dicot	Netherlands
**monocot**	**New Zealand**
**dicot**	**Greece**
dicot	New Zealand
**dicot**	**South Africa**
**dicot**	**USA**
**dicot**	**Ukraine**
**dicot**	**Colombia**
**dicot**	**Costa Rica**
**dicot**	**Dominica**
**dicot**	**Brazil**
**dicot**	**Brazil**
dicot	Denmark
dicot	Australia

Click here to access/download;Table;Table4.xlsx

Table 4. Conditions established for the identification of specific differentially expressed genes

host	dicot pathogenic			monocot pathogenic			monocot pathogenic			dicot pathogenic			Genes overexpressed in presence of:
Species	***C. higginsianum***			***C. graminicola***			***C. phormii***			***C. nympaheae***			
Condition	glucose vs DS	glucose vs MS	DS vs MS	glucose vs DS	glucose vs MS	DS vs MS	glucose vs DS	glucose vs MS	DS vs MS	glucose vs DS	glucose vs MS	DS vs MS	
0	↑	↑		↑	↑		↑	↑		↑	↑		glucose
1	↓	↓		↓	↓		↓	↓		↓	↓		PS
1a	↓	↓	↑	↓	↓		↓	↓		↓	↓	↑	PS and overexpressed in DS in dicot pathogens
1b	↓	↓		↓	↓	↓	↓	↓	↓	↓	↓		PS and overexpressed in MS in monocot pathogens
2		↓	↓		↓	↓		↓	↓		↓	↓	MS
3	↓		↑	↓		↑	↓		↑	↓		↑	DS
4	↓		↑							↓		↑	DS only in dicot pathogens
5					↓	↓		↓	↓				MS only in monocot pathogens
6				↓	↓		↓	↓					PS only in monocot pathogens
7	↓	↓								↓	↓		PS only in dicot pathogens

Arrows pointing up and highlighted in blue indicate overexpressed genes, while arrows pointing down and highlighted in red indicate downregulated genes
PS: plant substrate; MS: monocot substrate; DS: dicot substrate

C. higginsianum	glucose vs eudicot	↑	↓	↓
	glucose vs monocot	↑	↓	↓
	eudicot vs monocot			↑
C. graminicola	glucose vs eudicot	↑	↓	↓
	glucose vs monocot	↑	↓	↓
	eudicot vs monocot			
C. phormii	glucose vs eudicot	↑	↓	↓
	glucose vs monocot	↑	↓	↓
	eudicot vs monocot			
C. nympaheae	glucose vs eudicot	↑	↓	↓
	glucose vs monocot	↑	↓	↓
	eudicot vs monocot			↑

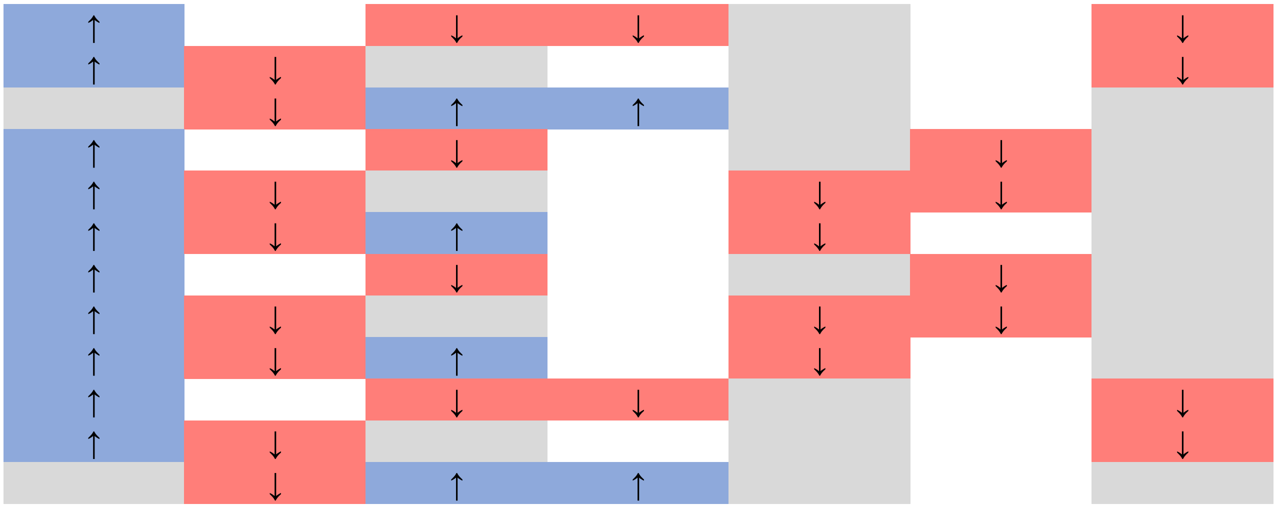

Click here to access/download;Figure;Figure1.png

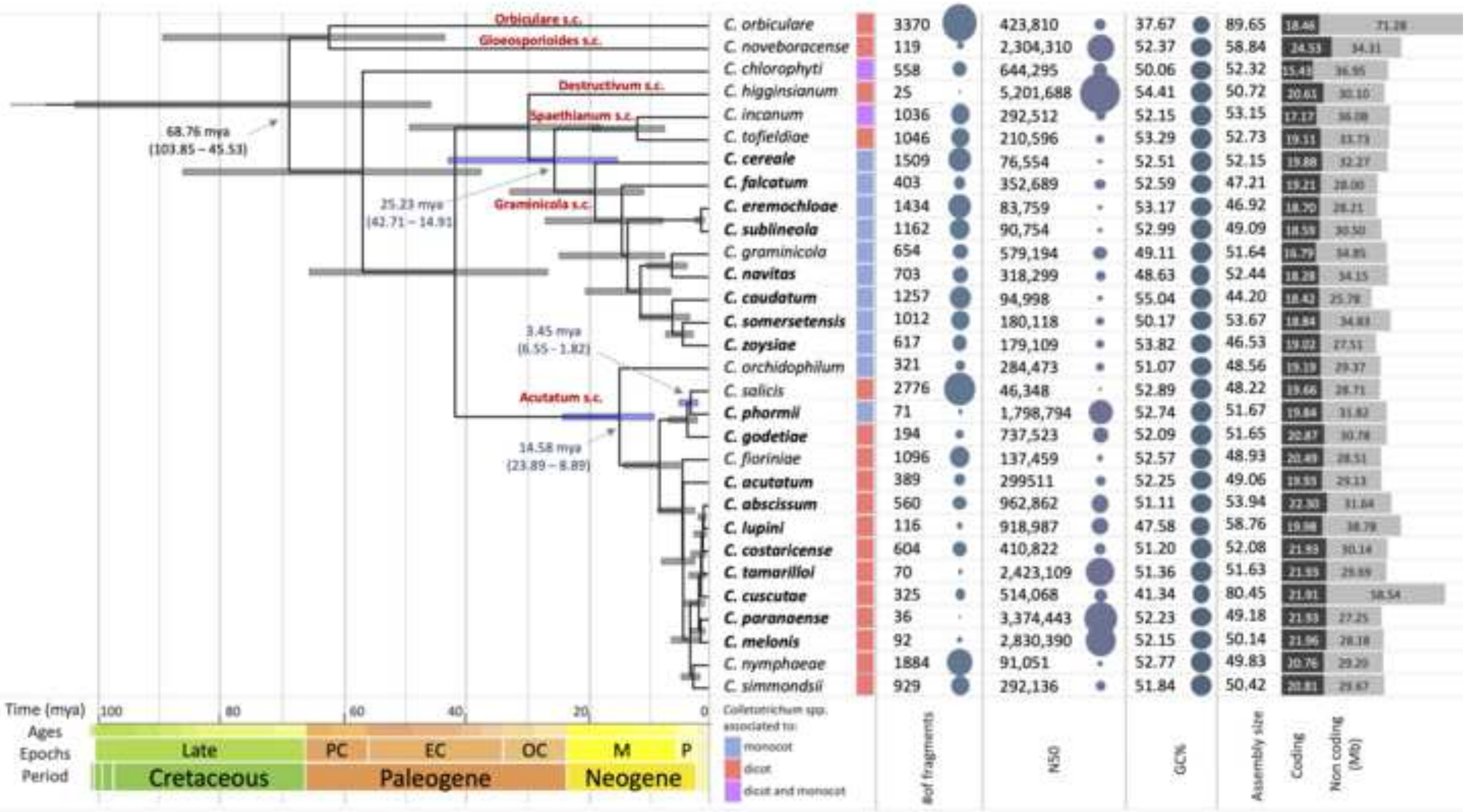

Click here to access/download;Figure;Figure2.png

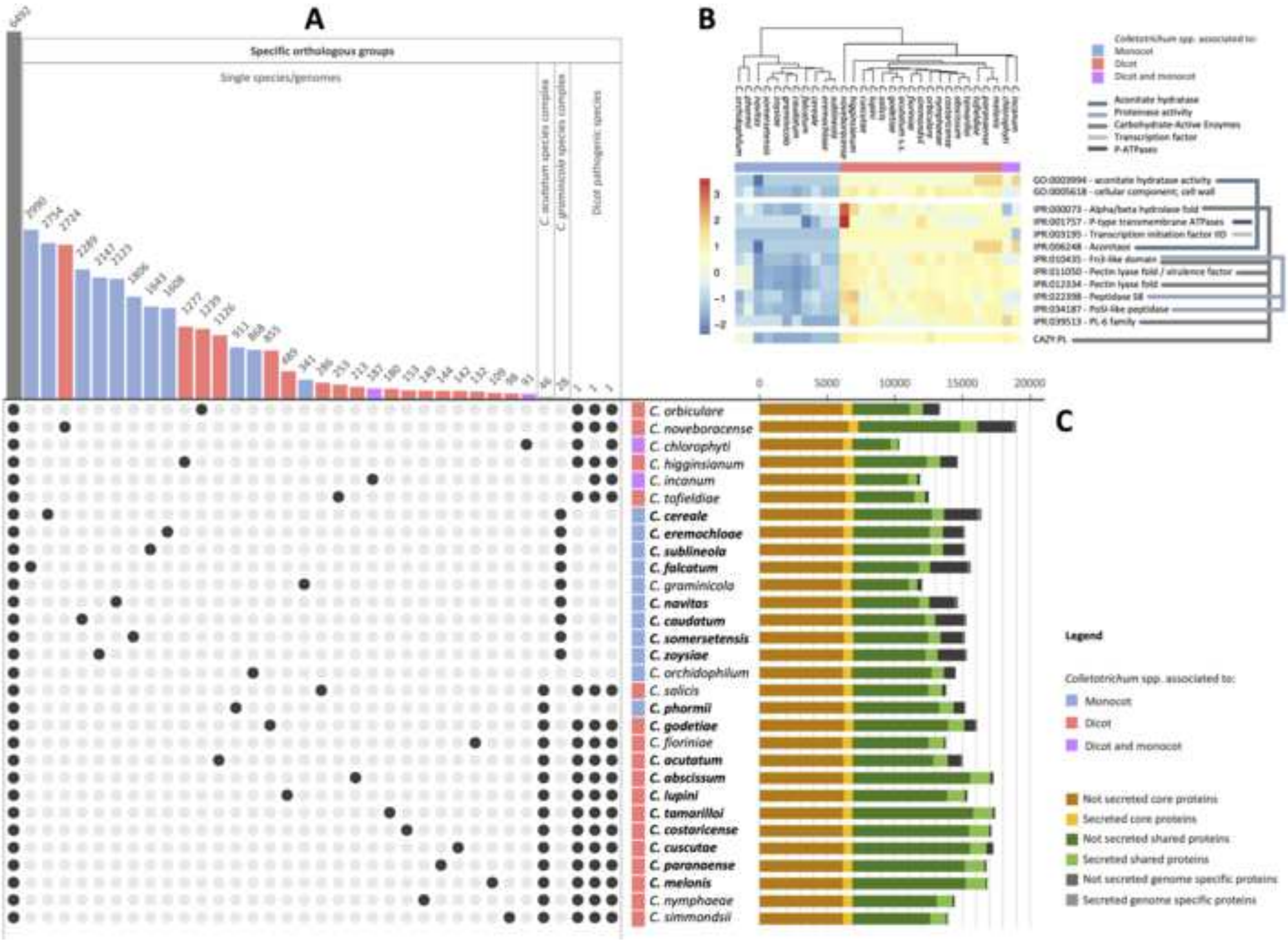

Click here to access/download;Figure;Figure3.png

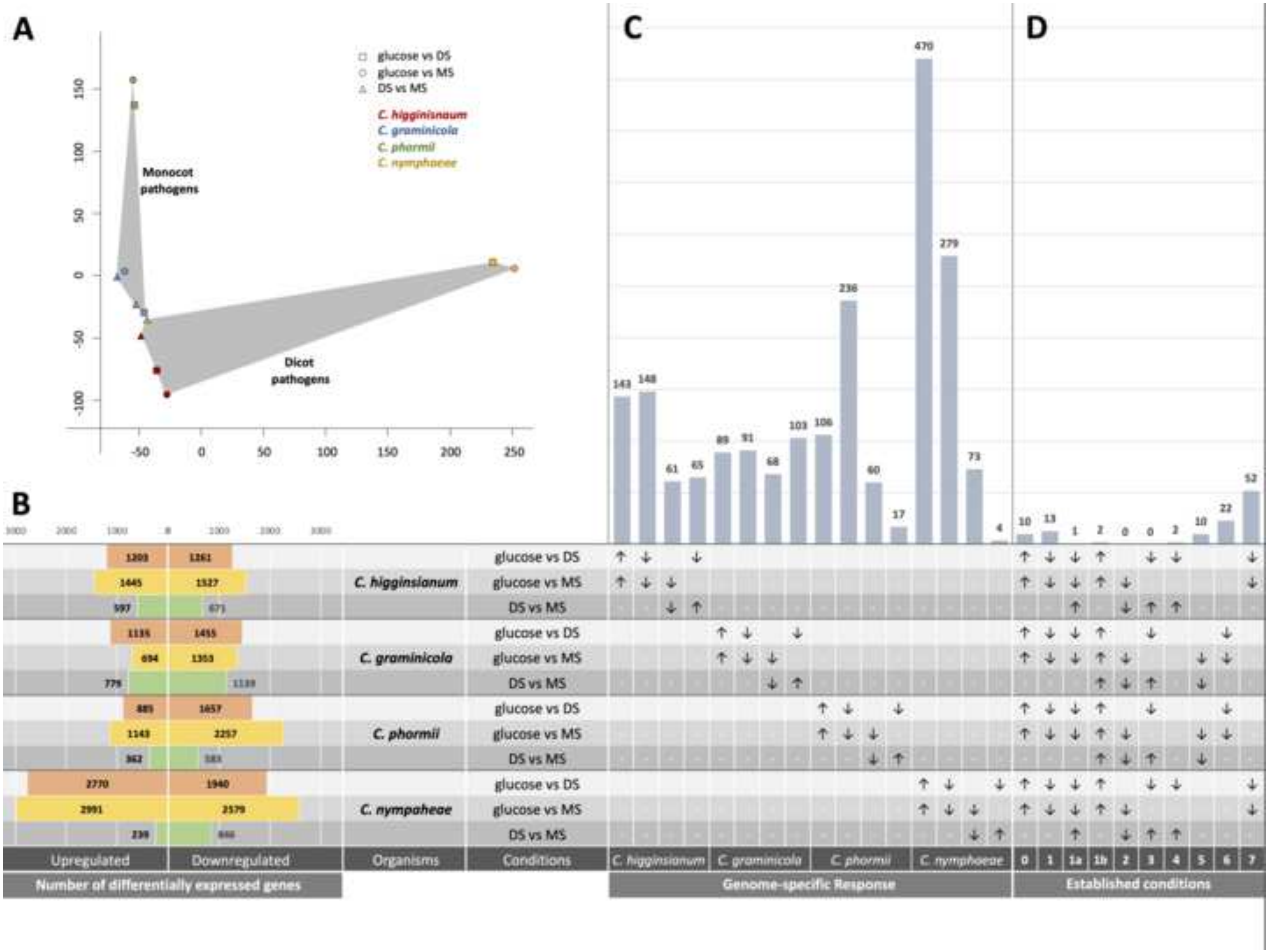

Click here to access/download;Figure;Figure4.png

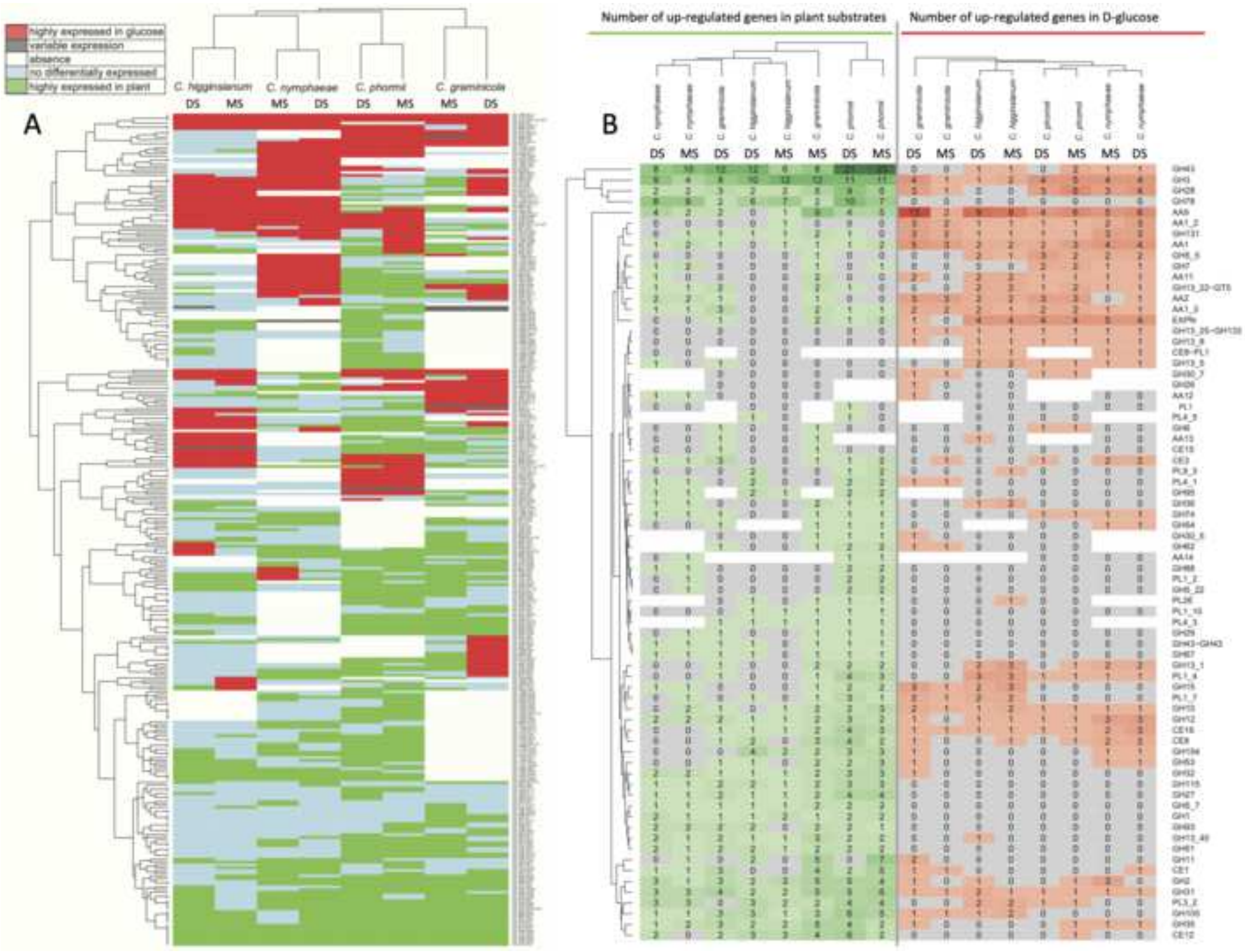

Click here to access/download
Supplementary Material
Suppl1_Calibrated_tree.pdf

Click here to access/download
Supplementary Material
Suppl2_GenomeData.xlsx

Click here to access/download
Supplementary Material
Suppl3_GO.xlsx

Click here to access/download
Supplementary Material
Suppl4_IPR.xlsx

Click here to access/download
Supplementary Material
Suppl5_Pfam.xlsx

Click here to access/download
Supplementary Material
Suppl6_CAZYmes_ALL.xlsx

Click here to access/download
Supplementary Material
Suppl7_CAZYmes_PBD.xlsx

Click here to access/download
Supplementary Material
Suppl8_MEROPS.xlsx

Click here to access/download
Supplementary Material
Suppl9_transporters.xlsx

Click here to access/download
Supplementary Material
Suppl10_TF.xlsx

Click here to access/download
Supplementary Material
Suppl11_Evo_genefamilies.pdf

Click here to access/download
Supplementary Material
Suppl12_replicates_heatmaps.pdf

Click here to access/download
Supplementary Material
Suppl13_Vulcano_plots.pdf

Click here to access/download
Supplementary Material
Suppl14_Orthoexpress_all.xlsx

Click here to access/download
Supplementary Material
Suppl15_material&methods-TB.pdf
