# Supplementary material for: Genome evolution and transcriptome plasticity is associated with adaptation to monocot and dicot plants in Colletotrichum fungi
Source: Gigascience. 2024 Jun 28;13:giae036. doi: 10.1093/gigascience/giae036 (PMC11212070; doi:10.1093/gigascience/giae036)
Supplement: giae036_Supplemental_Figures_and_Tables [file giae036_supplemental_figures_and_tables.zip › Supplementary Figure S1 - Calibrated tree.pdf]

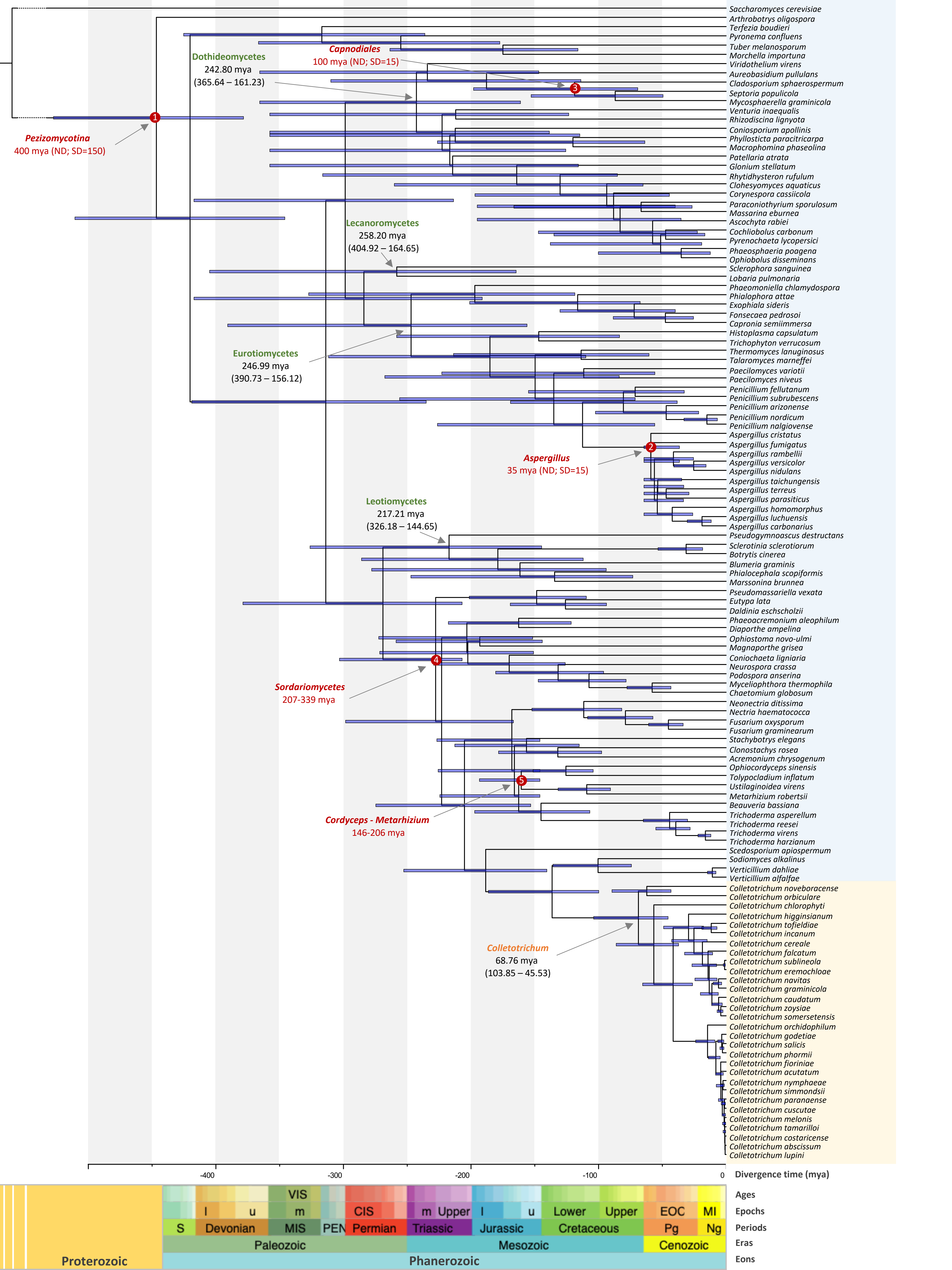
Saccharomyces cerevisiae
Arthrobotrys oligospora
Terfezia boudieri
Pyronema confluens
Tuber melanosporum
Morchella importuna
Viridothelium virens
Aureobasidium pullulans
Cladosporium sphaerospermum
Septoria populicola
Mycosphaerella graminicola
Venturia inaequalis
Rhizodiscina lignyota
Coniosporium apollinis
Phyllosticta paracitricarpa
Macrophomina phaseolina
Patellaria atrata
Glonium stellatum
Rhytidhysteron rufulum
Clohesyomyces aquaticus
Corynespora cassiicola
Paraconiothyrium sporulosum
Massarina eburnea
Ascochyta rabiei
Cochliobolus carbonum
Pyrenochaeta lycopersici
Phaeosphaeria poagena
Ophiobolus disseminans
Sclerophora sanguinea
Lobaria pulmonaria
Phaeomoniella chlamydospora
Phialophora attae
Exophiala sideris
Fonsecaea pedrosoi
Capronia semiimmersa
Histoplasma capsulatum
Trichophyton verrucosum
Thermomyces lanuginosus
Talaromyces marneffei
Paecilomyces variotii
Paecilomyces niveus
Penicillium fellutanum
Penicillium subrubescens
Penicillium arizonense
Penicillium nordicum
Penicillium nalgiovense
Aspergillus cristatus
Aspergillus fumigatus
Aspergillus rambellii
Aspergillus versicolor
Aspergillus nidulans
Aspergillus taichungensis
Aspergillus terreus
Aspergillus parasiticus
Aspergillus homomorphus
Aspergillus luchuensis
Aspergillus carbonarius
Pseudogymnoascus destructans
Sclerotinia sclerotiorum
Botrytis cinerea
Blumeria graminis
Phialocephala scopiformis
Marssonina brunnea
Pseudomassariella vexata
Eutypa lata
Daldinia eschscholzii
Phaeoacremonium aleophilum
Diaporthe ampelina
Ophiostoma novo-ulmi
Magnaporthe grisea
Coniochaeta ligniaria
Neurospora crassa
Podospora anserina
Myceliophthora thermophila
Chaetomium globosum
Neonectria ditissima
Nectria haematococca
Fusarium oxysporum
Fusarium graminearum
Stachybotrys elegans
Clonostachys rosea
Acremonium chrysogenum
Ophiocordyceps sinensis
Tolypocladium inflatum
Ustilaginoidea virens
Metarhizium robertsii
Beauveria bassiana
Trichoderma asperellum
Trichoderma reesei
Trichoderma virens
Trichoderma harzianum
Scedosporium apiospermum
Sodiomyces alkalinus
Verticillium dahliae
Verticillium alfalfae
Colletotrichum noveboracense
Colletotrichum orbiculare
Colletotrichum chlorophyti
Colletotrichum higginsianum
Colletotrichum tofieldiae
Colletotrichum incanum
Colletotrichum cereale
Colletotrichum falcatum
Colletotrichum sublineola
Colletotrichum eremochloae
Colletotrichum navitas
Colletotrichum graminicola
Colletotrichum caudatum
Colletotrichum zoysiae
Colletotrichum somersetensis
Colletotrichum orchidophilum
Colletotrichum godetiae
Colletotrichum salicis
Colletotrichum phormii
Colletotrichum fioriniae
Colletotrichum acutatum
Colletotrichum nymphaeae
Colletotrichum simmondsii
Colletotrichum paranaense
Colletotrichum cuscutae
Colletotrichum melonis
Colletotrichum tamarilloi
Colletotrichum costaricense
Colletotrichum abscissum
Colletotrichum lupini
Capnodiales
100 mya (ND; SD=15)
Dothideomycetes
242.80 mya
(365.64 – 161.23)
Pezizomycotina
400 mya (ND; SD=150)
Lecanoromycetes
258.20 mya
(404.92 – 164.65)
Eurotiomycetes
246.99 mya
(390.73 – 156.12)
Aspergillus
35 mya (ND; SD=15)
Leotiomycetes
217.21 mya
(326.18 – 144.65)
Sordariomycetes
207-339 mya
Cordyceps - Metarhizium
146-206 mya
Colletotrichum
68.76 mya
(103.85 – 45.53)
1
2
3
4
5
-400
-300
-200
-100
0
Divergence time (mya)
VIS
l
u
m
CIS
m
Upper
l
u
Lower
Upper
EOC
MI
S
Devonian
MIS
PEN
Permian
Triassic
Jurassic
Cretaceous
Pg
Ng
Paleozoic
Mesozoic
Cenozoic
Proterozoic
Phanerozoic
Ages
Epochs
Periods
Eras
Eons
