# Supplementary material for: Genome evolution and transcriptome plasticity is associated with adaptation to monocot and dicot plants in Colletotrichum fungi
Source: Gigascience. 2024 Jun 28;13:giae036. doi: 10.1093/gigascience/giae036 (PMC11212070; doi:10.1093/gigascience/giae036)

A

	C. eremochloae specific genes vs *C. sublineola* genome	*C. sublineola* specific genes vs *C. eremochloae* genome	*C. phormii* specific genes vs *C. salicis* genome	*C. salicis* specific genes vs *C. phormii* genome
**Number of genome specific genes**	1619	1661	869	288
**Genes that lack of similarity in the closest related genome**	62 (3.8%)	60 (3.6%)	38 (4.4%)	69 (2.4%)

B **Summary of BLASTN searches of genome specific genes versus the genome sequences of the closest related species**

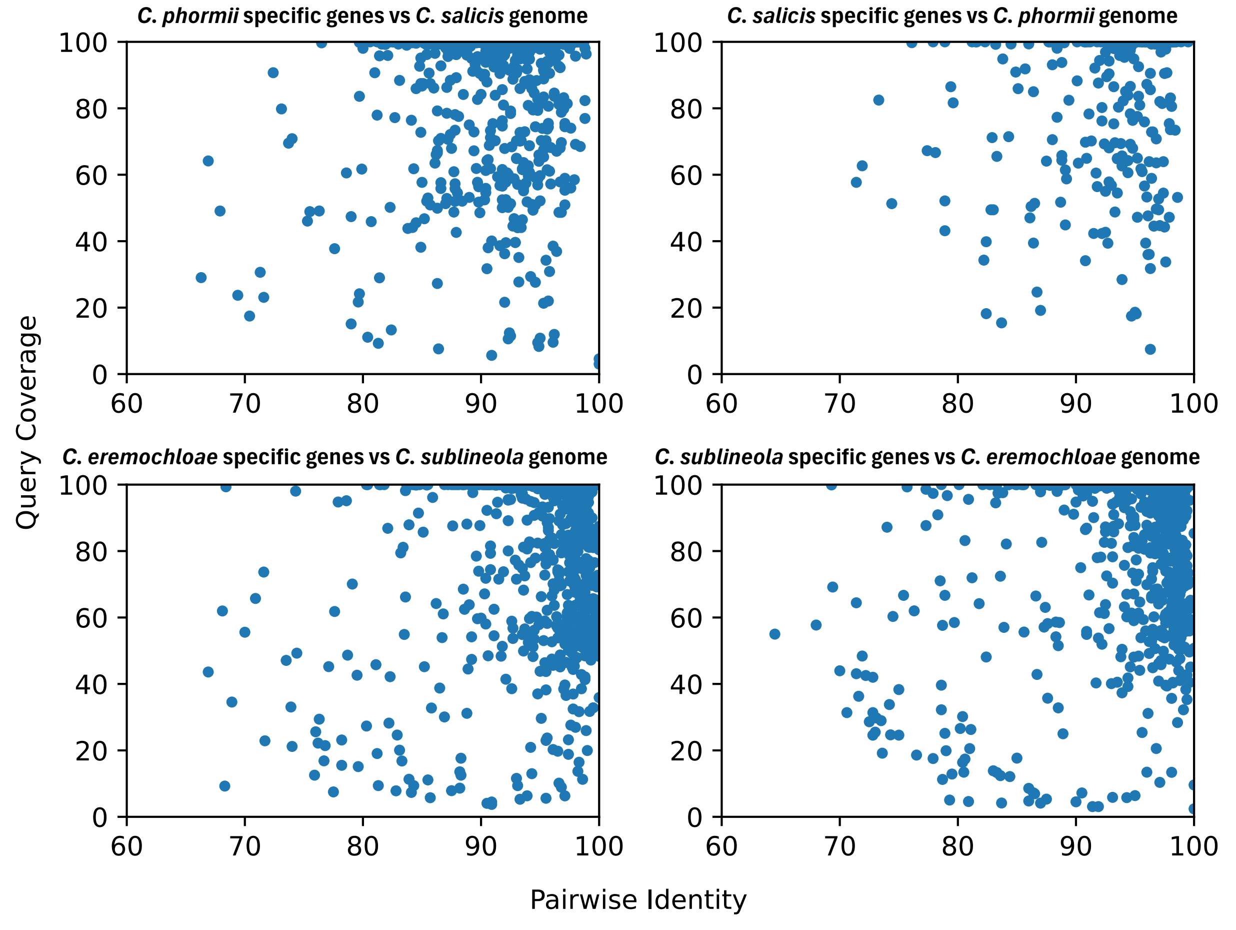

Supplement: giae036_Supplemental_Figures_and_Tables [file giae036_supplemental_figures_and_tables.zip › Supplementary Figure S2 - Species-specific genes.pdf]
