# Supplementary material for: Genome evolution and transcriptome plasticity is associated with adaptation to monocot and dicot plants in Colletotrichum fungi
Source: Gigascience. 2024 Jun 28;13:giae036. doi: 10.1093/gigascience/giae036 (PMC11212070; doi:10.1093/gigascience/giae036)

Transcription initiation factor IID, subunit 13 (IPR003195)

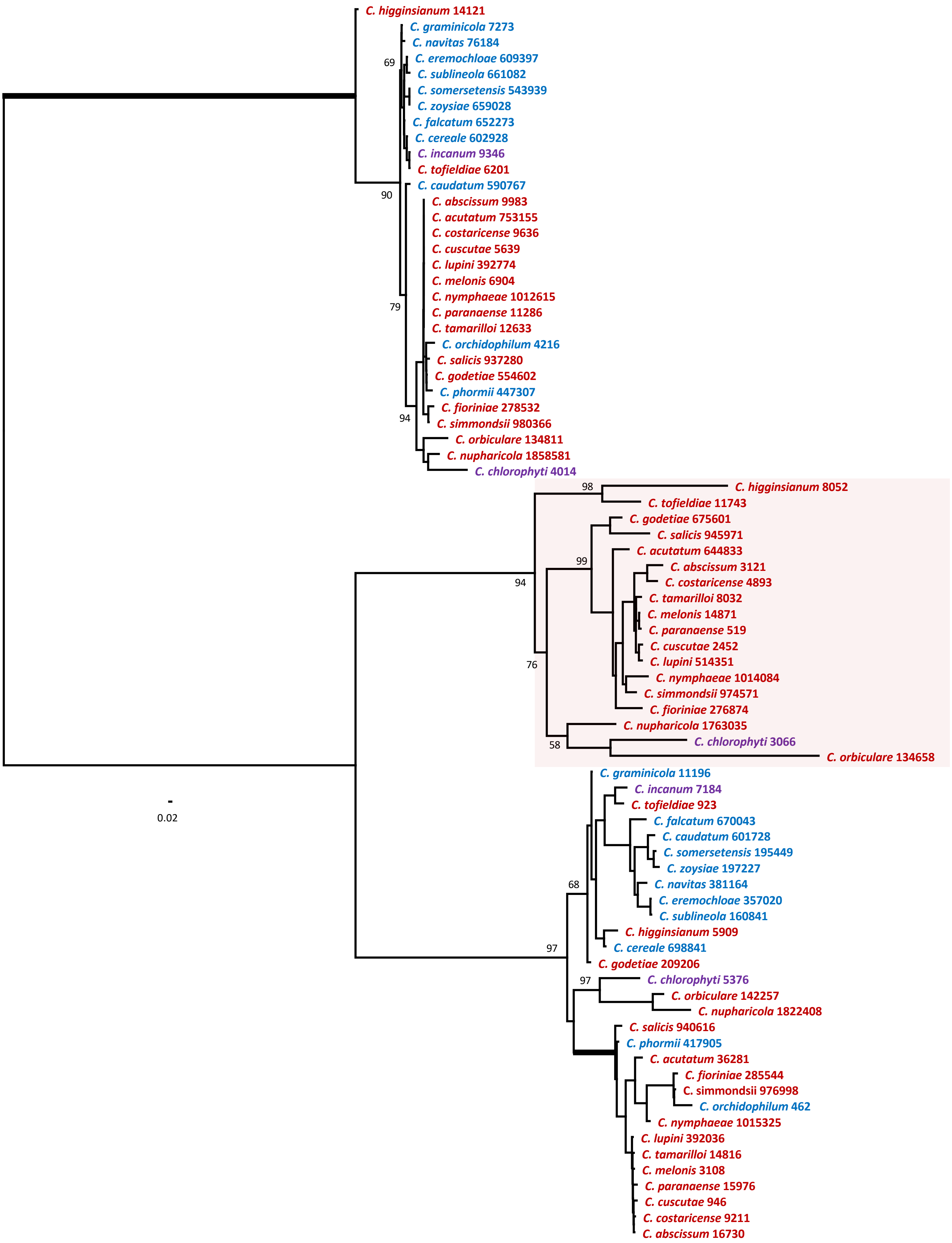

Aconitase, mitochondrial-like (IPR006248)

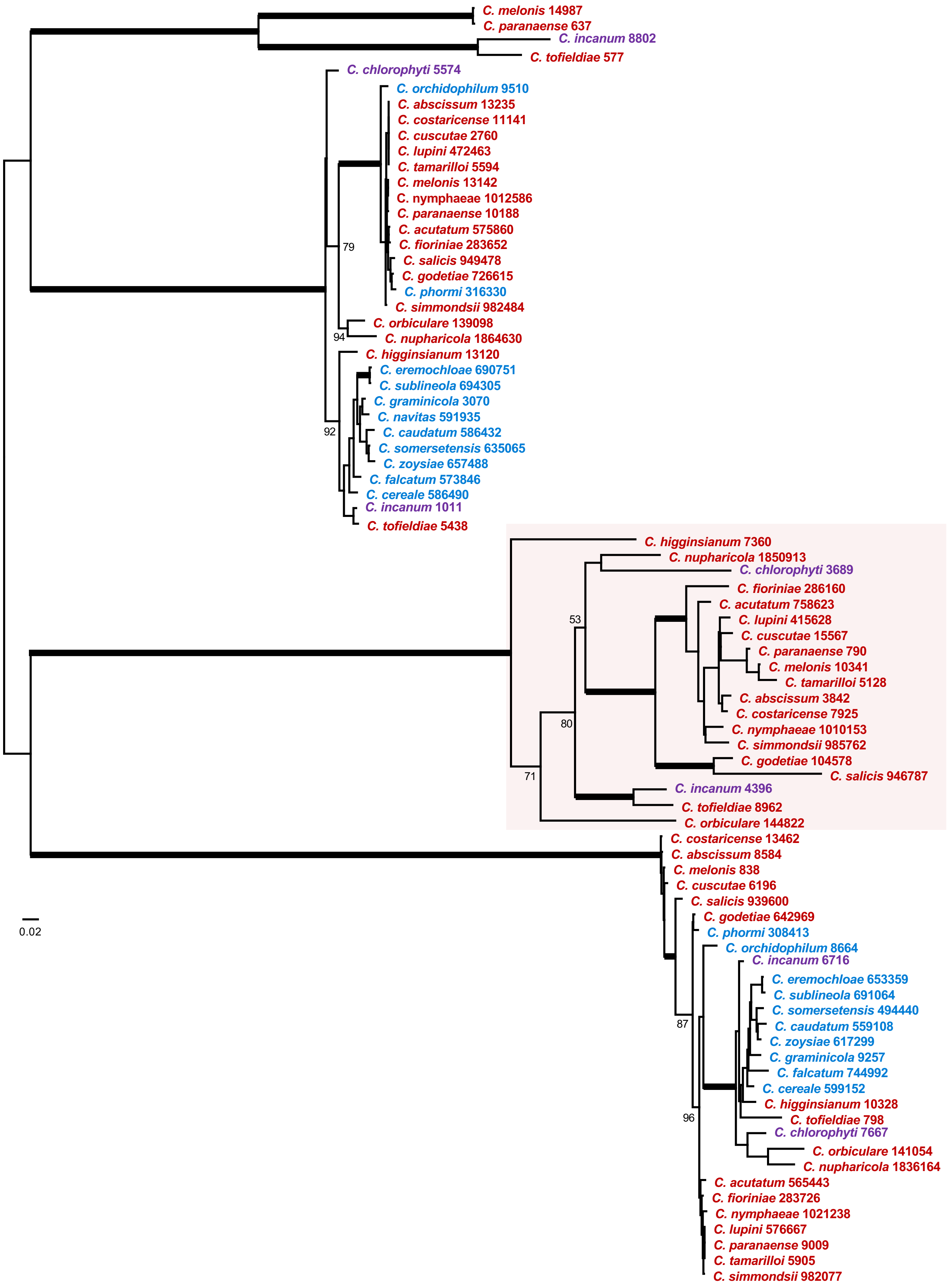

PoSl-like peptidase domain (IPR034187)

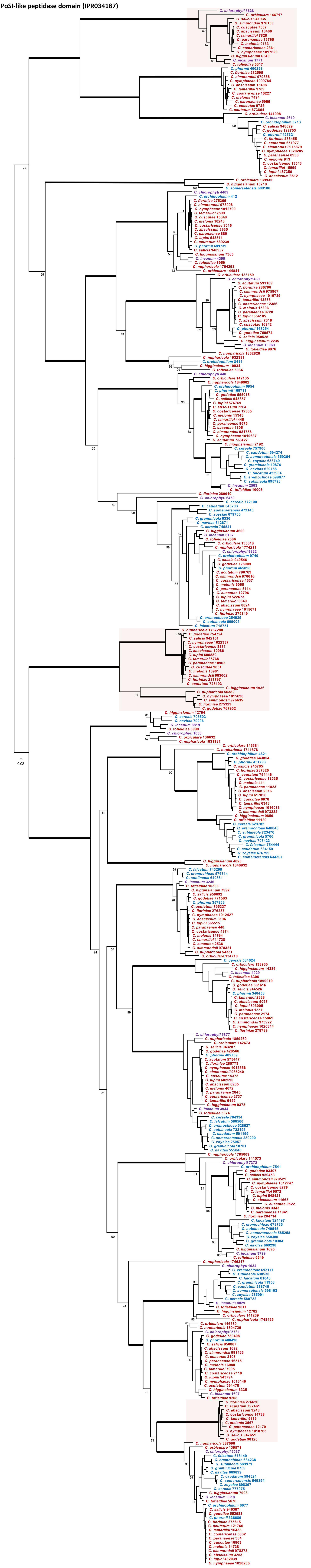

PL-6 family (IPR039513)

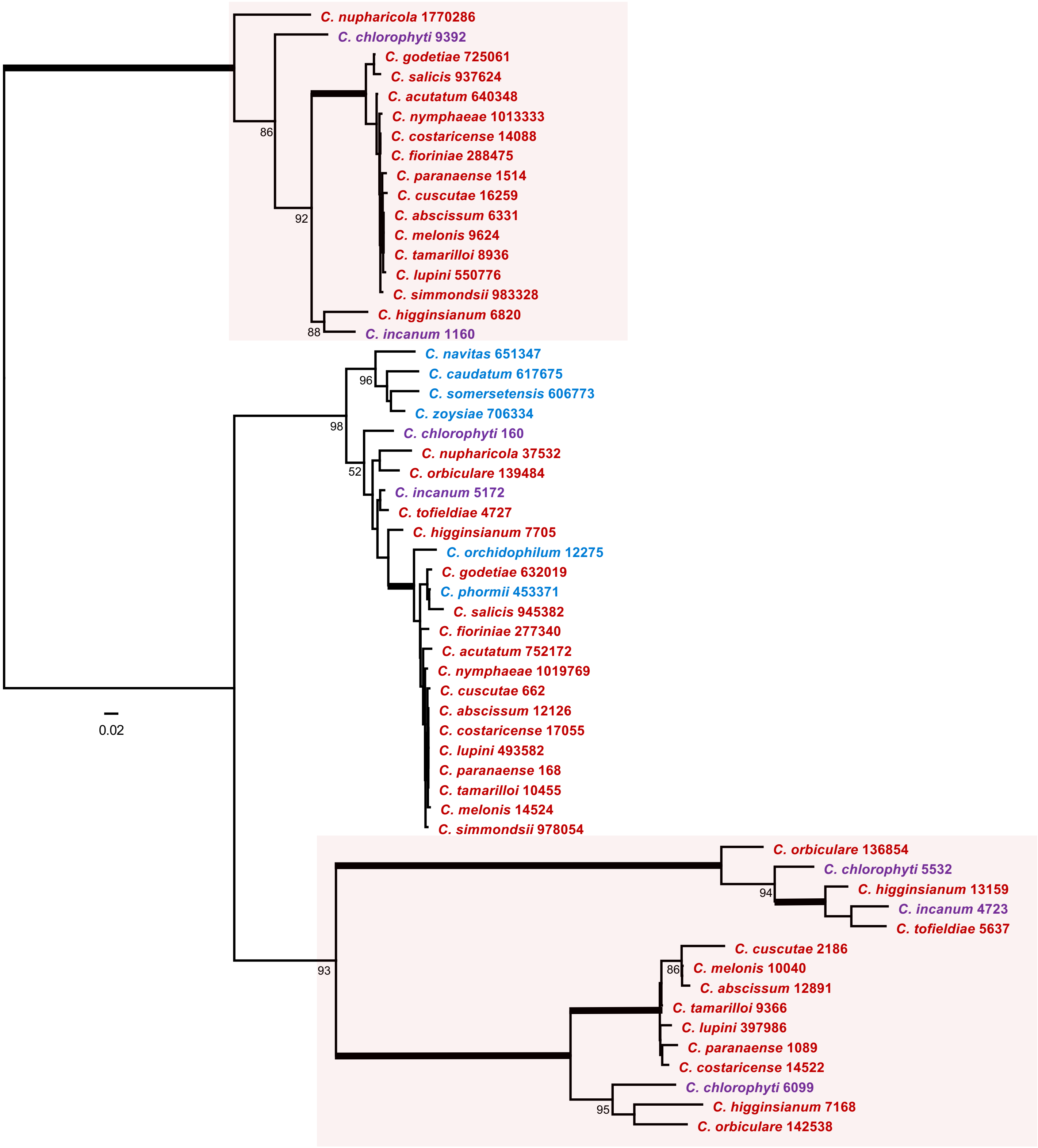

Supplement: giae036_Supplemental_Figures_and_Tables [file giae036_supplemental_figures_and_tables.zip › Supplementary Figure S3 - Gene families evo.pdf]
