# Supplementary material for: Genome evolution and transcriptome plasticity is associated with adaptation to monocot and dicot plants in Colletotrichum fungi
Source: Gigascience. 2024 Jun 28;13:giae036. doi: 10.1093/gigascience/giae036 (PMC11212070; doi:10.1093/gigascience/giae036)
Supplement: giae036_Supplemental_Figures_and_Tables [file giae036_supplemental_figures_and_tables.zip › Supplementary File S1 -Material and methods.pdf]

Supplementary File S15: Extended version of material ad methods used.

Nucleic acid purification.

DNA purification for PacBio sequencing.

Total genomic DNA was extracted using a SDS-CTAB method (Kim *et al.*, 1990) with some modifications. 200 mg of mycelium were placed into a 2 mL sterile extraction tube prefilled with 0.35 g of acid washed silica glass beads (0.5 mm) (Benchmark Scientific Inc., NJ). 50 mg of PVP40 (Sigma-Aldrich, Saint Louis, USA) and 400 µL of ice-cold lysis buffer (150 mM NaCl, 50 mM EDTA, 10 mM Tris-HCl pH 7.4, 30 µg mL^{-1} Proteinase K) were added to the extraction tubes prior homogenization.

The mycelium was homogenized using the bead-beating method through a BeadBug™ Microtube Homogenizer (Benchmark Scientific Inc., NJ). Three cycles of 30 s and 4000 rpm each, were followed by 30 s interval during which the samples were placed on ice.

Sodium dodecyl sulfate (SDS; Sigma-Aldrich, Saint Louis, USA) was added to a final concentration of 2% (w/v) and the mixture was incubated in a water bath at 65°C for 40 min. The lysed mixture was subsequently centrifuged for 10 min at 4°C and 2500 × *g*. The supernatant was transferred to a new tube and the volume was measured to adjust the NaCl concentration to 1.4 M and 1/10 volume of a 10% cetyltrimethylammonium-bromide (CTAB) buffer (10% CTAB, 500 mM Tris-HCl, 100 mM EDTA, pH 8.0) was added. After thorough mixing, the solution was incubated at 65°C for 10 min and cooled at 15°C for 2 min.

An equal volume of a solution of chloroform-isoamyl alcohol (24:1 v/v) was added to the mixture that was then centrifuged for 10 min at 4°C and 6700 × *g*.

The supernatant was transferred to a new tube and the DNA was precipitated with two volumes of 95% cold ethanol. Samples were stored at –20°C for a minimum of 1 hour and subsequently centrifuged 3 min at 4°C and 12,000 × *g*. The resulting pellet was rinsed once with 70% cold ethanol, vacuum-dried and dissolved in nuclease-free water (Promega, Madison, WI, USA). DNA solutions were stored at –20°C until use.

DNA purification for Illumina sequencing

Genomic DNA was extracted based on a modified CTAB procedure(Baek & Kenerley, 1998, p. 2). The mycelium (250 mg) was ground under liquid nitrogen using a sterilized chilled mortar and pestle. The resultant powder was mixed with 15 ml of a preheated solution (60°C) containing 10% CTAB, 2 M Tris-Cl (pH 8.0), 0,5 M EDTA, 1.4 M NaCl and 0.5% 2-mercaptoethanol. After incubation for 30 min at 60°C, samples were washed twice with a 15 ml volume of chloroform:isoamyl alcohol

24:1 (v/v). The aqueous phase was transferred to a clean tube, and the nucleic acids were precipitated with 0.6 volume of cold 2-propanol. After 2-hour incubation at room temperature, the samples were centrifuged for 2 min at 460 × *g*. The pellet was washed twice with 66% (v/v) EtOH and 34 % of 0.1 M NaCl. Tubes were centrifuged at 1500 × *g* for 10 min, washing buffer (supernatant) was removed and pellets were air dried in a fume hood (approximately 1 h). The pellets were resuspended in one ml of AB, left for few minutes, centrifuged for 5 min and supernatant (DNA) saved and pellet discarded.

RNA purification for transcriptomic analyses

A transfer experiment was performed for transcriptomics. 250 mL of complete medium (CM)(Vries *et al.*, 2004) containing 2% D-glucose in 1 L Erlenmeyer flasks was inoculated with 2.5 × 10^8 fresh spores, harvested from a MEA plate, and incubated in a rotatory shaker at 25°C for 20 h at 140 rpm. The mycelium was harvested by filtration, washed with liquid MM(Vries *et al.*, 2004) (without carbon source) and 2.5 g mycelium (wet weight) was transferred to 125 mL Erlenmeyer flasks containing 25 mL MM with 1% of maize powder (MS) or sugar beet pulp (DS), and incubated in a rotatory shaker at 25°C and 140 rpm. After pre-culturing and after 96 h of incubation in MS or DS, the mycelium was harvested by vacuum filtration, dried between tissue paper, directly frozen in liquid nitrogen and stored at −80°C(Klaubauf *et al.*, 2016). All experiments were performed in triplicate.

Total RNA was extracted from frozen mycelium ground in a Tissue Lyser (QIAGEN) using TRIzol reagent (Invitrogen) according to the manufacturer's instructions. RNA integrity and quantity were analysed on a 1% agarose electrophoresis gel and with the RNA6000 Nano Assay, using the Agilent 2100 Bioanalyzer (Agilent Technologies) (Klaubauf *et al.*, 2016).

Selection of the substrates was also based on their wide use by the scientific community and the known composition: the dry basis of sugar beet pulp is composed of (percentage by weight): 22–24 % of cellulose, 30 % of hemicelluloses and 15–25% of pectin, along with small amounts of fat (1.4%), protein (10.3%), ash (3.7%) and lignin (5.9%) (Tomaszewska *et al.*, 2018); the dry basis of maize powder is composed of (percentage by weight): 31-41 % of cellulose, 12-31 % of hemicelluloses, 2-10% of pectin and 11-31% of lignin, along with relatively small amounts of protein (7-12%) and ash (2-11%) (Woźniak *et al.*, 2021; Mensah *et al.*, 2021).

Genome sequencing and assembly

Five strains were sequenced using PacBio reads, namely, *C. godetiae* CBS 193.32, *C. acutatum* CBS 112980, *C. phormii* CBS 102054, *C. lupini* CBS 109225, and *C. navitas* CBS 125086. Genomic DNA

was sheared into fragments larger than 10 kb using a Covaris g-TUBE. The sheared DNA was treated with DNA damage repair mix followed by end repair and ligation of blunt adapters using SMRTbell Template Prep Kit 1.0 (Pacific Biosciences). The library was purified with AMPure PB beads. PacBio Sequencing primer was then annealed to the SMRTbell template library and Version P6 sequencing polymerase was bound to them. The prepared SMRTbell template libraries were then sequenced on a Pacific Biosciences RSII sequencer using Version C4 chemistry and 1x240 sequencing movie run times. The filtered subread data was assembled using Falcon version 0.2.2 (https://github.com/PacificBiosciences/FALCON), improved with finisherSC version 2.0 , and polished with Quiver version smrtanalysis_2.3.0.140936.p5 (https://github.com/PacificBiosciences/GenomicConsensus).

For the other seven strains *(C. cereale, C. eremochloae, C. sublineola, C. falcatum, C. caudatum, C. somersetensis, C. zoysiae),* genomic DNA was sheared to 300 bp using the Covaris LE220-Plus and size selected with SPRI using TotalPure NGS beads (Omega Bio-tek). The fragments were treated with end-repair, A-tailing, and ligation of Illumina compatible adapters (IDT, Inc) using the KAPA-HyperPrep kit (KAPA Biosystems). The prepared libraries were quantified using KAPA Biosystems' next-generation sequencing library qPCR kit and run on a Roche LightCycler 480 real-time PCR instrument. The quantified libraries were then prepared for sequencing on the Illumina HiSeq sequencing platform utilizing a TruSeq paired-end cluster kit, v4. Sequencing of the flowcell was performed on the Illumina HiSeq2500 sequencer using HiSeq TruSeq SBS sequencing kits, v4, following a 2x150 indexed run recipe. Raw reads filtered for artifact and process contamination were assembled with Velvet (Zerbino & Birney, 2008). The resulting assembly was used to create a long mate-pair library with insert 3000 +/- 300 bp which was then assembled with the original Illumina library with AllPathsLG release version R49403 (Gnerre *et al.*, 2011).

For transcriptomes, stranded cDNA libraries were generated using the Illumina Truseq Stranded mRNA Library Prep kit. mRNA was purified from 1 μg of total RNA using magnetic beads containing poly-T oligos. mRNA was fragmented and reversed transcribed using random hexamers and SSII (Invitrogen) followed by second strand synthesis. The fragmented cDNA was treated with end-pair, A-tailing, adapter ligation, and 8-10 cycles of PCR. The prepared libraries were quantified using KAPA Biosystems' next-generation sequencing library qPCR kit and run on a Roche LightCycler 480 real-time PCR instrument. The quantified libraries were then prepared for sequencing on the Illumina HiSeq sequencing platform utilizing a TruSeq paired-end cluster kit, v4. Sequencing of the flowcell was performed on the Illumina HiSeq2500 sequencer using HiSeq TruSeq SBS sequencing kits, v4, following a 2x150 indexed run recipe. RNA-Seq raw reads were

assembled into consensus sequences using either Rnnotator v3.3.2 (Martin *et al.*, 2010a) *(C. eremochloae, C. sublineola, C. falcatum, C. somersetensis, C. zoysiae*) or Trinity ver. 2.1.1 (Grabherr *et al.*, 2011) *(C.cereale, C. navitas, C. caudatum, C. godetiae, C. phormii, C. acutatum s.s.* and *C.lupini*). *C. abscissum*, *C. cuscutae*, *C. tamarilloi*, *C paranaense*, *C. costaricense*, *C. melonis* were sequenced using Illumina HiSeq 2500 Rapid-PE 250bp sequencing technology by the McGill University and Genome, Quebec Innovation Centre (Canada). Paired reads were assembled using SPAdes v3.8.2 (Bankevich *et al.*, 2012). Scaffolds were filtered for errors and contaminations based on low coverage value. High coverage fragments were manually checked by blastn against the nr database and scaffolds belonging to the mitochondrial genome and to the ribosomal cluster were masked.

Analysis of genome completeness

BUSCO v3 (Waterhouse *et al.*, 2018) (Benchmarking Universal Single-Copy Orthologs) was used to search the selected genomes for 3725 Sordariomycete orthologous genes (*sordariomyceta_odb9* data set) to assess the completeness of the genome sequences.

Gene annotation

The genome sequences of *C. cereale*, *C. eremochloae*, *C. sublineola*, *C. falcatum*, *C. navitas*, *C. caudatum*, *C. somersetensis*, *C. zoysiae*, *C. godetiae*, *C. phormii*, *C. acutatum s.s.* and *C. lupini* were annotated using the JGI annotation pipeline (Grigoriev *et al.*, 2014).

The MAKER2 v2.31.8 annotation pipeline (Holt & Yandell, 2011) was used to annotate the genome of *C. abscissum*, *C. cuscutae*, *C. tamarilloi*, *C paranaense*, *C. costaricense*, *C. melonis* as previously described (Baroncelli *et al.*, 2016a).

Phylogeny and divergence date estimation

The proteomes were clustered with OrthoFinder v0.4 (Emms & Kelly, 2015) and the clusters were analyzed with Mirlo (https://github.com/mthon/mirlo) to identify the 500 most phylogenetically informative single copy gene families. The families were aligned with MAFFT 7 (Katoh & Standley, 2013) and then concatenated. A substitution model and its parameter values were selected using ProtTest 3.4 (Abascal *et al.*, 2005). A phylogenetic tree was reconstructed using Bayesian MCMC analysis from the concatenated alignment of the 500 genes identified with Mirlo under the WAG + I evolutionary model and the gamma distribution calculated using four rate categories and homogeneous rates across the tree. The posterior probability threshold was 50%.

A selection of 126 genomes covering the Pezizomycotina plus the genome of *Saccharomyces cerevisiae* as an outgroup were selected from the MycoCosm database (Supplementary 1) and analyzed. The calibrated tree was inferred by applying the RelTime method (Tamura *et al.*, 2012, 2018) to the supplied phylogenetic tree whose branch lengths were calculated using the Ordinary Least Squares method using MEGA X v10.1.7 (Kumar *et al.*, 2018).

The timetree was computed using 5 calibration point (3 fossil records and 2 estimated constraints):

1: Paleopyrenomycites on the crown group of Pezizomycotina, thus assuming the common ancestor of all filamentous, sporocarp-producing Ascomycota (Pezizomycotina) to be 400 mya (Taylor *et al.*, 1999, 2005; Lücking *et al.*, 2009) (normal distribution; standard deviation = 150).

2: *Aspergillus collembolorum* representing the common ancestor of the genus *Aspergillus* was constrained to an age of 35 mya (Dörfelt & Schmidt, 2005) (normal distribution; standard deviation = 15)

3: The fossil Metacapnodiaceae (Schmidt *et al.*, 2014) representing the common ancestor of the order Capnodiales was constrained to an age of 100 mya (normal distribution; standard deviation = 50)

4: Sordariomycetes crown to an age of 207-339 mya (Beimforde *et al.*, 2014) (equal distribution)

5: *Cordyceps* - *Metarhizium* divergence to an age of 146-206 mya (Sung *et al.*, 2008) (equal distribution)

The Tao method was used to set minimum and maximum time boundaries on nodes for which calibration densities were provided (Tao *et al.*, 2020). The evolutionary distances were computed using the Poisson correction method (Zuckerkandl & Pauling, 1965) and are in the units of the number of amino acid substitutions per site. This analysis involved 127 amino acid sequences and total of 124023 positions in the final dataset. Evolutionary analyses were conducted in MEGA X (Kumar *et al.*, 2018).

Table A. Genomes used to build the phylogenomic tree presented in "Supplementary File S1" with relative information and references. Genomes sequenced in this work are in bold.

Tree position	Genus	isolates	Database	Reference
0	*Saccharomyces cerevisiae*	S288C	JGI	(Goffeau *et al.*, 1996)
1	*Arthrobotrys oligospora*	ATCC 24927	JGI	(Yang *et al.*, 2011)
2	*Terfezia boudieri*	ATCC MYA-4762	JGI	(Murat *et al.*, 2018)
3	*Pyronema confluens*	CBS 100304	JGI	(Traeger *et al.*, 2013)

4	*Tuber melanosporum*	Mel28 v1.2	JGI	(Martin *et al.*, 2010b)
5	*Morchella importuna*	CCBAS932	JGI	(Murat *et al.*, 2018)
6	*Viridothelium virens*		JGI	(Haridas *et al.*, 2020)
7	*Aureobasidium pullulans*	CBS 110374	JGI	(Gostinčar *et al.*, 2014)
8	*Cladosporium sphaerospermum*	UM 843	JGI	(Ng *et al.*, 2012)
9	*Septoria populicola*		JGI	(Ohm *et al.*, 2012)
10	*Mycosphaerella graminicola*		JGI	(Goodwin *et al.*, 2011)
11	*Venturia inaequalis*		JGI	(Deng *et al.*, 2017)
12	*Rhizodiscina lignyota*	CBS 133067	JGI	(Haridas *et al.*, 2020)
13	*Coniosporium apollinis*	CBS 100218	JGI	(Teixeira *et al.*, 2017)
14	*Phyllosticta paracitricarpa*	CBS 141357	JGI	(Guarnaccia *et al.*, 2019)
15	*Macrophomina phaseolina*	MS6	JGI	(Islam *et al.*, 2012)
16	*Patellaria atrata*		JGI	(Haridas *et al.*, 2020)
17	*Glonium stellatum*	CBS 207.34	JGI	(Peter *et al.*, 2016)
18	*Rhytidhysteron rufulum*		JGI	(Ohm *et al.*, 2012)
19	*Clohesyomyces aquaticus*		JGI	(Mondo *et al.*, 2017)
20	*Corynespora cassiicola*	CCP	JGI	(Lopez *et al.*, 2018)
21	*Paraconiothyrium sporulosum*	AP3s5-JAC2a	JGI	(Zeiner *et al.*, 2016)
22	*Massarina eburnea*	CBS 473.64	JGI	(Haridas *et al.*, 2020)
23	*Ascochyta rabiei*	ArDII	JGI	(Verma *et al.*, 2016)
24	*Cochliobolus carbonum*	26-R-13	JGI	(Condon *et al.*, 2013)
25	*Pyrenochaeta lycopersici*	MPI-SDFR-AT-0127	JGI	(Mesny *et al.*, 2021)
26	*Phaeosphaeria poagena*	MPI-PUGE-AT-0046c	JGI	(Mesny *et al.*, 2021)
27	*Ophiobolus disseminans*	CBS 113818	JGI	(Haridas *et al.*, 2020)
28	*Sclerophora sanguinea*	CBS 100924	JGI	-
29	*Lobaria pulmonaria*	Scotland reference	JGI	-
30	*Phaeomoniella chlamydospora*	UCRPC4	JGI	(Morales-Cruz *et al.*, 2015)
31	*Phialophora attae*	CBS 131958	JGI	(Moreno *et al.*, 2015)
32	*Exophiala sideris*	CBS 121828	JGI	(Teixeira *et al.*, 2017)
33	*Fonsecaea pedrosoi*	CBS 271.37	JGI	(Teixeira *et al.*, 2017)
34	*Capronia semiimmersa*	CBS 27337	JGI	(Teixeira *et al.*, 2017)
35	*Histoplasma capsulatum*	NAm1	JGI	(Sharpton *et al.*, 2009)
36	*Trichophyton verrucosum*	HKI 517	JGI	(Burmester *et al.*, 2011)
37	*Thermomyces lanuginosus*	SSBP	JGI	(Mchunu *et al.*, 2013)
38	*Talaromyces marneffei*	ATCC 18224	JGI	(Nierman *et al.*, 2015)
39	*Paecilomyces variotii*	CBS 101075	JGI	(Urquhart *et al.*, 2018)
40	*Paecilomyces niveus*	CO7	JGI	(Biango-Daniels *et al.*, 2018)
41	*Penicillium fellutanum*	ATCC 48694	JGI	-

42	*Penicillium subrubescens*	CBS 132785	JGI	(Peng *et al.*, 2017)
43	*Penicillium arizonense*	CBS 141311	JGI	(Grijseels *et al.*, 2016)
44	*Penicillium nordicum*	DAOMC 185683	JGI	(Wingfield *et al.*, 2015)
45	*Penicillium nalgiovense*	FM193	JGI	(Nielsen *et al.*, 2017)
46	*Aspergillus cristatus*	GZAAS20.1005	JGI	(Ge *et al.*, 2016)
47	*Aspergillus fumigatus*	A1163	JGI	(Fedorova *et al.*, 2008)
48	*Aspergillus rambellii*	SRRC1468	JGI	(Moore *et al.*, 2016)
49	*Aspergillus versicolor*		JGI	(de Vries *et al.*, 2017)
50	*Aspergillus nidulans*		JGI	(Arnaud *et al.*, 2012)
51	*Aspergillus taichungensis*	IBT 19404	JGI	(Kjærbølling *et al.*, 2018)
52	*Aspergillus terreus*	NIH 2624	JGI	(Arnaud *et al.*, 2012)
53	*Aspergillus parasiticus*	CBS 117618	JGI	(Kjærbølling *et al.*, 2018)
54	*Aspergillus homomorphus*	CBS 101889	JGI	(Vesth *et al.*, 2018)
55	*Aspergillus luchuensis*	CBS 106.47	JGI	(de Vries *et al.*, 2017)
56	*Aspergillus carbonarius*	ITEM 5010	JGI	(de Vries *et al.*, 2017)
57	*Pseudogymnoascus destructans*	20631-21	JGI	(Drees *et al.*, 2016)
58	*Sclerotinia sclerotiorum*		JGI	(Drees *et al.*, 2016)
59	*Botrytis cinerea*		JGI	(Staats & van Kan, 2012)
60	*Blumeria graminis*	96224	JGI	(Müller *et al.*, 2019)
61	*Phialocephala scopiformis*	5WS22E1	JGI	(Walker *et al.*, 2016)
62	*Marssonina brunnea*	MB_m1	JGI	(Zhu *et al.*, 2012)
63	*Pseudomassariella vexata*	CBS 129021	JGI	(Mondo *et al.*, 2017)
64	*Eutypa lata*	UCREL1	JGI	(Blanco-Ulate *et al.*, 2013a)
65	*Daldinia eschscholzii*	EC12	JGI	(Wu *et al.*, 2017)
66	*Phaeoacremonium aleophilum*	UCRPA7	JGI	(Blanco-Ulate *et al.*, 2013b)
67	*Diaporthe ampelina*	UCDDA912	JGI	(Morales-Cruz *et al.*, 2015)
68	*Ophiostoma novo-ulmi*	H327	JGI	(Forgetta *et al.*, 2013)
69	*Magnaporthe grisea*		JGI	(Dean *et al.*, 2005)
70	*Coniochaeta ligniaria*	NRRL 30616	JGI	(Jiménez *et al.*, 2017)
71	*Neurospora crassa*	OR74A	JGI	(Galagan *et al.*, 2003)
72	*Podospora anserina*	S mat+	JGI	(Espagne *et al.*, 2008)
73	*Myceliophthora thermophila*		JGI	(Berka *et al.*, 2011)
74	*Chaetomium globosum*	MPI-SDFR-AT-0079	JGI	(Mesny *et al.*, 2021)
75	*Neonectria ditissima*	R09/05	JGI	(Gómez-Cortecero *et al.*, 2015)
76	*Nectria haematococca*		JGI	(Coleman *et al.*, 2009)
77	*Fusarium oxysporum*	4287	JGI	(Ma *et al.*, 2010)
78	*Fusarium graminearum*		JGI	(Cuomo *et al.*, 2007)
79	*Stachybotrys elegans*	MPI-CAGE-CH-0235	JGI	(Mesny *et al.*, 2021)

80	*Clonostachys rosea*	IK726	JGI	(Karlsson *et al.*, 2015)
81	*Acremonium chrysogenum*	ATCC 11550	JGI	(Terfehr *et al.*, 2014)
82	*Ophiocordyceps sinensis*	IOZ07	NCBI	(Shu *et al.*, 2020)
83	*Tolypocladium inflatum*	NRRL 8044	JGI	(Bushley *et al.*, 2013)
84	*Ustilaginoidea virens*		JGI	(Kumagai *et al.*, 2016)
85	*Metarhizium robertsii*	ARSEF 23	NCBI	(Hu *et al.*, 2014)
86	*Beauveria bassiana*	ARSEF 2860	JGI	(Xiao *et al.*, 2012)
87	*Trichoderma asperellum*	CBS 433.97	JGI	(Druzhinina *et al.*, 2018)
88	*Trichoderma reesei*	QM6a	JGI	(Li *et al.*, 2017)
89	*Trichoderma virens*	Gv29-8	JGI	(Kubicek *et al.*, 2011)
90	*Trichoderma harzianum*	TR274	JGI	(Kubicek *et al.*, 2019)
91	*Scedosporium apiospermum*	IHEM 14462	JGI	(Vandeputte *et al.*, 2014)
92	*Sodiomyces alkalinus*		JGI	(Grum-Grzhimaylo *et al.*, 2018)
93	*Verticillium dahliae*	VdLs.17	JGI	(Klosterman *et al.*, 2011)
94	*Verticillium alfalfae*	VaMs.102	JGI	(Klosterman *et al.*, 2011)
95	*Colletotrichum noveboracense*	23	JGI	-
96	*Colletotrichum orbiculare*	MAFF 240422	JGI	(Gan *et al.*, 2013)
97	*Colletotrichum chlorophyti*	NTL11	JGI	(Gan *et al.*, 2017)
98	*Colletotrichum higginsianum*	IMI 349063	JGI	(Zampounis *et al.*, 2016)
99	*Colletotrichum tofieldiae*	861	JGI	(Hacquard *et al.*, 2016)
100	*Colletotrichum incanum*	MAFF 238712	JGI	(Gan *et al.*, 2016)
**101**	***Colletotrichum cereale***	**CBS 129662**	**JGI**	**This work**
**102**	***Colletotrichum falcatum***	**MAFF 306170**	**JGI**	**This work**
**103**	***Colletotrichum sublineola***	**CBS 131301**	**JGI**	**This work**
**104**	***Colletotrichum eremochloae***	**CBS 129661**	**JGI**	**This work**
**105**	***Colletotrichum navitas***	**CBS 125086**	**JGI**	**This work**
106	*Colletotrichum graminicola*	M1.001	JGI	(O'Connell *et al.*, 2012)
**107**	***Colletotrichum caudatum***	**CBS 131602**	**JGI**	**This work**
**108**	***Colletotrichum zoysiae***	**MAFF 235873**	**JGI**	**This work**
**109**	***Colletotrichum somersetensis***	**CBS 131599**	**JGI**	**This work**
110	*Colletotrichum orchidophilum*	IMI 309357	JGI	(Baroncelli *et al.*, 2018)
**111**	***Colletotrichum godetiae***	**CBS 193.32**	**JGI**	**This work**
112	*Colletotrichum salicis*	CBS 607.94	JGI	(Baroncelli *et al.*, 2016b)
**113**	***Colletotrichum phormii***	**CBS 102054**	**JGI**	**This work**
114	*Colletotrichum fioriniae*	IMI 504882	JGI	(Baroncelli *et al.*, 2016b)
**115**	***Colletotrichum acutatum***	**CBS 112980**	**JGI**	**This work**
116	*Colletotrichum nymphaeae*	IMI 504889	JGI	(Baroncelli *et al.*, 2016b)
117	*Colletotrichum simmondsii*	CBS 122122	JGI	(Baroncelli *et al.*, 2016b)

118	*Colletotrichum paranaense*	IMI 384185	JGI	This work
119	*Colletotrichum cuscutae*	IMI 304802	JGI	This work
120	*Colletotrichum melonis*	CBS 134730	JGI	This work
121	*Colletotrichum tamarilloi*	CBS 129955	JGI	This work
122	*Colletotrichum costaricense*	IMI 309622	JGI	This work
123	*Colletotrichum abscissum*	IMI 504890	JGI	This work
124	*Colletotrichum lupini*	CBS 109225	JGI	This work

Annotation of specific gene categories

Proteins that are transported out of the cell and into the extracellular space were identified with SignalP-4.1 (Petersen *et al.*, 2011). Protein domains were annotated using Pfam (Sonnhammer *et al.*, 1997) and InterPro(Apweiler *et al.*, 2001) and mapped to Gene Ontology (GO) terms (Ashburner *et al.*, 2000). CAZymes were annotated using CAZy pipeline (Lombard *et al.*, 2014). Peptidases were annotated with the MEROPS database, a hierarchical, structure based classification for peptidases, organized into families and clans (https://www.ebi.ac.uk/merops/) (Rawlings *et al.*, 2012).

BLASTp (Altschul *et al.*, 1990) and RunIprScan (http://michaelrthon.com/runiprscan/) results were used to manually identify genes encoding enzymes that are signatures of backbone secondary metabolite (SM) genes in the Ascomycota (Schardl *et al.*, 2013): nonribosomal peptide synthetases (NRPS; IPR010071, IPR006163, IPR001242), polyketide synthases (PKS; IPR013968), DMATS-family aromatic prenyltransferases (IPR017795, Pfam PF11991), and terpene synthases/cyclases (IPR008949).

Transcription factors were identified using BLASTp against NCBI non-redundant protein sequences (nr) database and the Aspergillus Genome Database (AspGD) (Cerqueira *et al.*, 2014). P value of 1e-10 was used as cutoff in both cases. NCBI conserved Domains Database(CCD) and EMBL Simple Modular Architecture Research Tool (SMART) (https://smart.embl.de) (Letunic & Bork, 2018) were used to manually assign putative function(s) to uncharacterized transcription factors.

Cys_6Zn_2 and Cys_2His_2 regulators were also analysed by phylogenetic analyses (NJ) using orthologs of all kingdoms of known regulators involved in plant biomass degradation (Benocci *et al.*, 2017).

Comparative genomics

Ortholog identification and protein cluster analyses

The Markov Cluster algorithm (mcl v14-137 (Enright *et al.*, 2002)) was used for the identification of protein clusters while (Co-)orthologous groups were identified by Proteinortho v5.16b (Lechner *et al.*, 2011).

<u>The pan- and core-genome and lineage specific genes</u>

1 - The pan-genome: all genes present in one or more species.

2 - The core-genome: genes present in all included species, including paralogs. This set is expected to encode cellular functions needed for all species.

3 – Lineage specific genes: genes found in only one species or cluster in our analysis, with or without paralogs (Included in these, we would expect to find genes involved in environmental adaptation. This group can also include annotation errors)

<u>Identification of expansions and contractions of gene families associated with PS</u>

Functional categories associated with mono- or dicot pathogenic species were identified using two different statistical analyses.

Disjoint sets calculated as:

Set 1 = monocot pathogens

Set 2 = dicot pathogens

if (Min Set1 > Max Set2) than term is overrepresented in Set1

if (Min Set2 > Max Set1) than term is overrepresented in Set2

Terms enriched based on Fisher's exact test were calculated for each in each genome in the following subset: secretomes, all core proteins, secreted core proteins, all shared proteins, secreted shared proteins, all species-specific proteins, and secreted species-specific proteins. Profiles were compared to identify terms enriched only in monocot or dicot pathogens.

Transcription profiles of *Colletotrichum* spp. on monocot and dicot plant cell walls

The CAZy families related to plant biomass degradation were selected based on a previous study (Daly *et al.*, 2018). We compared gene expression of the selected CAZy genes between transcriptome of fungi growth on glucose and the other two carbon sources (substrates containing different plant cell walls). The expression difference was binned into the following five categories: highly expressed in glucose, variable expression, absence, not differentially expressed and lowly expressed in glucose for each ortholog gene(s) of each species in each specific comparison. The variable expression means more than one gene were included in the ortholog group of a specific species and the expression of these genes show different expression trend in comparison. Only all the orthologs of a species show the same highly or lowly expression in glucose were defined as

differentially expressed gene. The no differentially expression means the gene expression show no significant difference between glucose and other two carbon sources. The absence means the ortholog gene were not detected for specific species. These categories were coded with -2, -1, 0, 1, 2 respectively, and were visualized with heatmap using R package “gplots”, with the complete-linkage clustering method and Euclidean distance. For each CAZy family, we did similar analysis. Instead of summarizing the expression difference between glucose and other carbon sources to different categories, the numbers of genes of highly or lowly expressed in glucose condition were visualized in the heatmap. To distinguish the numbers of highly and lowly expressed gene observed in comparison of transcriptome of fungi growth in glucose and other two carbon sources, we transferred numbers of lowly expressed genes to the corresponding negative values in clustering calculation.

Identification and analysis of differential gene expression

Stranded RNASeq library(s) were created and quantified by qPCR as described earlier in the “Nucleic acid purification” section. Sequencing was performed using Illumina HiSeq2500 following a 2x100 indexed run recipe. Raw fastq file reads were filtered and trimmed for quality and contamination. Filtered RNA-Seq reads from each library were aligned to the corresponding reference genome using HISAT version 0.1.4-beta (Kim *et al.*, 2015). featureCounts (Liao *et al.*, 2014) was used to generate the raw gene counts using genome annotations. Only primary hits assigned to the reverse strand were included in the raw gene counts (-s 2 -p --primary options). DESeq2 version 1.10.0 (Love *et al.*, 2014, p. 2) was subsequently used to determine which genes were differentially expressed between pairs of conditions. The parameters used to call a gene differentially expressed between conditions were log2FoldChange > 2 and p-value < 0.05.

Comparative transcriptomics

A custom script *orthoexpress.pl* was developed based on Proteinortho v5.16b (Lechner *et al.*, 2011) output to identify groups of genes showing specific expression patterns.

Recent duplications were manually checked. In case of different behavior of paralogs both forms of the (co-)orthologous groups were analyzed independently.

Seven logical conditions (Table 4) were established to identify genes differentially expressed in specific organisms/conditions.

Description of the established conditions of transcription profiles are reported below:

- Condition 0: genes overexpressed in presence of glucose
- Condition 1: genes overexpressed in presence of plant biomass (PS)
- Condition 1a: genes overexpressed in presence of PS and overexpressed in dicot substrate (DS) in dicot pathogens
- Condition 1b: genes overexpressed in presence of PS and overexpressed in monocot substrate (MS) in monocot pathogens
- Condition 2: genes overexpressed in presence of MS.
- Condition 3: genes overexpressed in presence of DS.
- Condition 4: genes overexpressed in presence of DS only in dicot pathogens.
- Condition 5: genes overexpressed in presence of MS only in monocot pathogens.
- Condition 6: genes overexpressed in presence of PS only in monocot pathogens.
- Condition 7: genes overexpressed in presence of PS only in dicot pathogens.

REFERENCES

Abascal F, Zardoya R, Posada D. 2005. ProtTest: selection of best-fit models of protein evolution. *Bioinformatics* 21: 2104–2105.

Altschul SF, Gish W, Miller W, Myers EW, Lipman DJ. 1990. Basic local alignment search tool. *Journal of Molecular Biology* 215: 403–410.

Apweiler R, Attwood TK, Bairoch A, Bateman A, Birney E, Biswas M, Bucher P, Cerutti L, Corpet F, Croning MD, *et al.* 2001. The InterPro database, an integrated documentation resource for protein families, domains and functional sites. *Nucleic Acids Research* 29: 37–40.

Arnaud MB, Cerqueira GC, Inglis DO, Skrzypek MS, Binkley J, Chibucos MC, Crabtree J, Howarth C, Orvis J, Shah P, *et al.* 2012. The *Aspergillus* Genome Database (AspGD): recent developments in comprehensive multispecies curation, comparative genomics and community resources. *Nucleic Acids Research* 40: D653–D659.

Ashburner M, Ball CA, Blake JA, Botstein D, Butler H, Cherry JM, Davis AP, Dolinski K, Dwight SS, Eppig JT, *et al.* 2000. Gene Ontology: tool for the unification of biology. *Nature Genetics* 25: 25–29.

Baek J-M, Kenerley CM. 1998. The arg2 Gene of *Trichoderma virens*: cloning and development of a homologous transformation system. *Fungal Genetics and Biology* 23: 34–44.

Bankevich A, Nurk S, Antipov D, Gurevich AA, Dvorkin M, Kulikov AS, Lesin VM, Nikolenko SI, Pham S, Prjibelski AD, *et al.* 2012. SPAdes: a new genome assembly algorithm and its applications to single-cell sequencing. *Journal of Computational Biology* 19: 455–477.

Baroncelli R, Amby DB, Zapparata A, Sarrocco S, Vannacci G, Le Floch G, Harrison RJ, Holub E, Sukno SA, Sreenivasaprasad S, *et al.* 2016a. Gene family expansions and contractions are associated with host range in plant pathogens of the genus *Colletotrichum*. *BMC Genomics* 17: 555.

Baroncelli R, Amby DB, Zapparata A, Sarrocco S, Vannacci G, Le Floch G, Harrison RJ, Holub E, Sukno SA, Sreenivasaprasad S, *et al.* 2016b. Gene family expansions and contractions are associated with host range in plant pathogens of the genus Colletotrichum. *BMC Genomics* 17.

Baroncelli R, Sukno SA, Sarrocco S, Cafà G, Le Floch G, Thon MR. 2018. Whole-genome sequence of the orchid anthracnose pathogen *Colletotrichum orchidophilum*. *Molecular Plant-Microbe Interactions* 31: 979–981.

Beimforde C, Feldberg K, Nylinder S, Rikkinen J, Tuovila H, Dörfelt H, Gube M, Jackson DJ, Reitner J, Seyfullah LJ, *et al.* 2014. Estimating the phanerozoic history of the Ascomycota lineages: combining fossil and molecular data. *Molecular Phylogenetics and Evolution*.

Benocci T, Aguilar-Pontes MV, Zhou M, Seiboth B, de Vries RP. 2017. Regulators of plant biomass degradation in ascomycetous fungi. *Biotechnology for Biofuels* 10: 152.

Berka RM, Grigoriev IV, Otillar R, Salamov A, Grimwood J, Reid I, Ishmael N, John T, Darmond C, Moisan M-C, *et al.* 2011. Comparative genomic analysis of the thermophilic biomass-degrading fungi *Myceliophthora thermophila* and *Thielavia terrestris*. *Nature Biotechnology* 29: 922–927.

Biango-Daniels MN, Wang TW, Hodge KT. 2018. Draft Genome Sequence of the Patulin-Producing Fungus *Paecilomyces niveus* Strain CO7. *Genome Announcements* 6: e00556-18.

Blanco-Ulate B, Rolshausen PE, Cantu D. 2013a. Draft Genome Sequence of the Grapevine Dieback Fungus *Eutypa lata* UCR-EL1. *Genome Announcements* 1.

Blanco-Ulate B, Rolshausen P, Cantu D. 2013b. Draft Genome Sequence of the Ascomycete *Phaeoacremonium aleophilum* Strain UCR-PA7, a Causal Agent of the Esca Disease Complex in Grapevines. *Genome Announcements* 1: e00390-13.

Burmester A, Shelest E, Glöckner G, Heddergott C, Schindler S, Staib P, Heidel A, Felder M, Petzold A, Szafranski K, *et al.* 2011. Comparative and functional genomics provide insights into the pathogenicity of dermatophytic fungi. *Genome Biology* 12: R7.

Bushley KE, Raja R, Jaiswal P, Cumbie JS, Nonogaki M, Boyd AE, Owensby CA, Knaus BJ, Elser J, Miller D, *et al.* 2013. The Genome of *Tolypocladium inflatum*: Evolution, Organization, and Expression of the Cyclosporin Biosynthetic Gene Cluster. *PLOS Genetics* 9: e1003496.

Cerqueira GC, Arnaud MB, Inglis DO, Skrzypek MS, Binkley G, Simison M, Miyasato SR, Binkley J, Orvis J, Shah P, *et al.* 2014. The *Aspergillus* Genome Database: multispecies curation and incorporation of RNA-Seq data to improve structural gene annotations. *Nucleic Acids Research* 42: D705–D710.

Coleman JJ, Rounsley SD, Rodriguez-Carres M, Kuo A, Wasmann CC, Grimwood J, Schmutz J, Taga M, White GJ, Zhou S, *et al.* 2009. The Genome of *Nectria haematococca*: Contribution of Supernumerary Chromosomes to Gene Expansion. *PLOS Genetics* 5: e1000618.

Condon BJ, Leng Y, Wu D, Bushley KE, Ohm RA, Otillar R, Martin J, Schackwitz W, Grimwood J, MohdZainudin N. 2013. Comparative genome structure, secondary metabolite, and effector coding capacity across Cochliobolus pathogens. *PLoS Genet* 9.

Cuomo CA, Güldener U, Xu J-R, Trail F, Turgeon BG, Pietro AD, Walton JD, Ma L-J, Baker SE, Rep M, *et al.* 2007. The *Fusarium graminearum* genome reveals a link between localized polymorphism and pathogen specialization. *Science* 317: 1400–1402.

Daly P, López SC, Peng M, Lancefield CS, Purvine SO, Kim Y-M, Zink EM, Dohnalkova A, Singan VR, Lipzen A, *et al.* 2018. *Dichomitus squalens* partially tailors its molecular responses to the composition of solid wood. *Environmental Microbiology* 20: 4141–4156.

Dean RA, Talbot NJ, Ebbole DJ, Farman ML, Mitchell TK, Orbach MJ, Thon M, Kulkarni R, Xu J-R, Pan H, *et al.* 2005. The genome sequence of the rice blast fungus *Magnaporthe grisea*. *Nature* 434: 980–986.

Deng CH, Plummer KM, Jones DAB, Mesarich CH, Shiller J, Taranto AP, Robinson AJ, Kastner P, Hall NE, Templeton MD, *et al.* 2017. Comparative analysis of the predicted secretomes of Rosaceae scab pathogens *Venturia inaequalis* and *V. pirina* reveals expanded effector families and putative determinants of host range. *BMC Genomics* 18: 339.

Dörfelt H, Schmidt AR. 2005. A fossil *Aspergillus* from Baltic amber. *Mycological Research* 109: 956–960.

Drees KP, Palmer JM, Sebra R, Lorch JM, Chen C, Wu C-C, Bok JW, Keller NP, Blehert DS, Cuomo CA, *et al.* 2016. Use of Multiple Sequencing Technologies To Produce a High-Quality Genome of the Fungus *Pseudogymnoascus destructans*, the Causative Agent of Bat White-Nose Syndrome. *Genome Announcements* 4: e00445-16.

Druzhinina IS, Chenthamara K, Zhang J, Atanasova L, Yang D, Miao Y, Rahimi MJ, Grujic M, Cai F, Pourmehdi S, *et al.* 2018. Massive lateral transfer of genes encoding plant cell wall-degrading enzymes to the mycoparasitic fungus *Trichoderma* from its plant-associated hosts. *PLOS Genetics* 14: e1007322.

Emms DM, Kelly S. 2015. OrthoFinder: solving fundamental biases in whole genome comparisons dramatically improves orthogroup inference accuracy. *Genome Biology* 16.

Enright AJ, Van Dongen S, Ouzounis CA. 2002. An efficient algorithm for large-scale detection of protein families. *Nucleic Acids Research* 30: 1575–1584.

Espagne E, Lespinet O, Malagnac F, Da Silva C, Jaillon O, Porcel BM, Couloux A, Aury J-M, Ségurens B, Poulain J, *et al.* 2008. The genome sequence of the model ascomycete fungus *Podospora anserina*. *Genome Biology* 9: R77.

Fedorova ND, Khaldi N, Joardar VS, Maiti R, Amedeo P, Anderson MJ, Crabtree J, Silva JC, Badger JH, Albarraq A, *et al.* 2008. Genomic Islands in the Pathogenic Filamentous Fungus *Aspergillus fumigatus*. *PLOS Genetics* 4: e1000046.

Forgetta V, Leveque G, Dias J, Grove D, Lyons R, Genik S, Wright C, Singh S, Peterson N, Zianni M, *et al.* 2013. Sequencing of the Dutch Elm Disease Fungus Genome Using the Roche/454 GS-FLX Titanium System in a Comparison of Multiple Genomics Core Facilities. *Journal of Biomolecular Techniques : JBT* 24: 39–49.

Galagan JE, Calvo SE, Borkovich KA, Selker EU, Read ND, Jaffe D, FitzHugh W, Ma L-J, Smirnov S, Purcell S, *et al.* 2003. The genome sequence of the filamentous fungus *Neurospora crassa*. *Nature* 422: 859–868.

Gan P, Ikeda K, Irieda H, Narusaka M, O'Connell RJ, Narusaka Y, Takano Y, Kubo Y, Shirasu K. 2013. Comparative genomic and transcriptomic analyses reveal the hemibiotrophic stage shift of *Colletotrichum* fungi. *New Phytologist* 197: 1236–1249.

Gan P, Narusaka M, Kumakura N, Tsushima A, Takano Y, Narusaka Y, Shirasu K. 2016. Genus-wide comparative genome analyses of *Colletotrichum* species reveal specific gene family losses and gains during adaptation to specific infection lifestyles. *Genome Biology and Evolution* 8: 1467–1481.

Gan P, Narusaka M, Tsushima A, Narusaka Y, Takano Y, Shirasu K. 2017. Draft Genome Assembly of *Colletotrichum chlorophyti*, a Pathogen of Herbaceous Plants. *Genome Announcements* 5.

Ge Y, Wang Y, Liu Y, Tan Y, Ren X, Zhang X, Hyde KD, Liu Y, Liu Z. 2016. Comparative genomic and transcriptomic analyses of the Fuzhuan brick tea-fermentation fungus *Aspergillus cristatus*. *BMC genomics* 17: 428.

Gnerre S, MacCallum I, Przybylski D, Ribeiro FJ, Burton JN, Walker BJ, Sharpe T, Hall G, Shea TP, Sykes S, *et al.* 2011. High-quality draft assemblies of mammalian genomes from massively parallel sequence data. *Proceedings of the National Academy of Sciences* 108: 1513–1518.

Goffeau A, Barrell BG, Bussey H, Davis RW, Dujon B, Feldmann H, Galibert F, Hoheisel JD, Jacq C, Johnston M, *et al.* 1996. Life with 6000 genes. *Science (New York, N.Y.)* 274: 546, 563–567.

Gómez-Cortecero A, Harrison RJ, Armitage AD. 2015. Draft Genome Sequence of a European Isolate of the Apple Canker Pathogen *Neonectria ditissima*. *Genome Announcements* 3: e01243-15.

Goodwin SB, M'barek SB, Dhillon B, Wittenberg AHJ, Crane CF, Hane JK, Foster AJ, Van der Lee TAJ, Grimwood J, Aerts A, *et al.* 2011. Finished genome of the fungal wheat pathogen *Mycosphaerella graminicola* reveals dispensome structure, chromosome plasticity, and stealth pathogenesis. *PLoS genetics* 7: e1002070.

Gostinčar C, Ohm RA, Kogej T, Sonjak S, Turk M, Zajc J, Zalar P, Grube M, Sun H, Han J, *et al.* 2014. Genome sequencing of four *Aureobasidium pullulans* varieties: biotechnological potential, stress tolerance, and description of new species. *BMC Genomics* 15: 549.

Grabherr MG, Haas BJ, Yassour M, Levin JZ, Thompson DA, Amit I, Adiconis X, Fan L, Raychowdhury R, Zeng Q, *et al.* 2011. Full-length transcriptome assembly from RNA-Seq data without a reference genome. *Nature Biotechnology* 29: 644–652.

Grigoriev IV, Nikitin R, Haridas S, Kuo A, Ohm R, Otillar R, Riley R, Salamov A, Zhao X, Korzeniewski F, *et al.* 2014. MycoCosm portal: gearing up for 1000 fungal genomes. *Nucleic Acids Research* 42: D699-704.

Grijseels S, Nielsen JC, Randelovic M, Nielsen J, Nielsen KF, Workman M, Frisvad JC. 2016. *Penicillium arizonense*, a new, genome sequenced fungal species, reveals a high chemical diversity in secreted metabolites. *Scientific Reports* 6: 35112.

Grum-Grzhimaylo AA, Falkoski DL, van den Heuvel J, Valero-Jiménez CA, Min B, Choi I-G, Lipzen A, Daum CG, Aanen DK, Tsang A, *et al.* 2018. The obligate alkalophilic soda-lake fungus *Sodiomyces alkalinus* has shifted to a protein diet. *Molecular Ecology* 27: 4808–4819.

Guarnaccia V, Gehrmann T, Silva-Junior GJ, Fourie PH, Haridas S, Vu D, Spatafora J, Martin FM, Robert V, Grigoriev IV, *et al.* 2019. *Phyllosticta citricarpa* and sister species of global importance to *Citrus*. *Molecular Plant Pathology* 20: 1619–1635.

Hacquard S, Kracher B, Hiruma K, Münch PC, Garrido-Oter R, Thon MR, Weimann A, Damm U, Dallery J-F, Hainaut M, *et al.* 2016. Survival trade-offs in plant roots during colonization by closely related beneficial and pathogenic fungi. *Nature Communications* 7: 11362.

Haridas S, Albert R, Binder M, Bloem J, LaButti K, Salamov A, Andreopoulos B, Baker SE, Barry K, Bills G, *et al.* 2020. 101 *Dothideomycetes* genomes: a test case for predicting lifestyles and emergence of pathogens. *Studies in Mycology* 96: 141–153.

Holt C, Yandell M. 2011. MAKER2: an annotation pipeline and genome-database management tool for second-generation genome projects. *BMC Bioinformatics* 12: 491.

Hu X, Xiao G, Zheng P, Shang Y, Su Y, Zhang X, Liu X, Zhan S, St. Leger RJ, Wang C. 2014. Trajectory and genomic determinants of fungal-pathogen speciation and host adaptation. *Proceedings of the National Academy of Sciences* 111: 16796–16801.

Islam MS, Haque MS, Islam MM, Emdad EM, Halim A, Hossen QMM, Hossain MZ, Ahmed B, Rahim S, Rahman MS, *et al.* 2012. Tools to kill: Genome of one of the most destructive plant pathogenic fungi *Macrophomina phaseolina*. *BMC Genomics* 13: 493.

Jiménez DJ, Hector RE, Riley R, Lipzen A, Kuo RC, Amirebrahimi M, Barry KW, Grigoriev IV, van Elsas JD, Nichols NN. 2017. Draft Genome Sequence of *Coniochaeta ligniaria* NRRL 30616, a Lignocellulolytic Fungus for Bioabatement of Inhibitors in Plant Biomass Hydrolysates. *Genome Announcements* 5: e01476-16.

Karlsson M, Durling MB, Choi J, Kosawang C, Lackner G, Tzelepis GD, Nygren K, Dubey MK, Kamou N, Levasseur A, *et al.* 2015. Insights on the Evolution of Mycoparasitism from the Genome of *Clonostachys rosea*. *Genome Biology and Evolution*.

Katoh K, Standley DM. 2013. MAFFT multiple sequence alignment software version 7: improvements in performance and usability. *Molecular Biology and Evolution* 30: 772–780.

Kim D, Langmead B, Salzberg SL. 2015. HISAT: a fast spliced aligner with low memory requirements. *Nature Methods* 12: 357–360.

Kim WK, Mauthe W, Hausner G, Klassen GR. 1990. Isolation of high molecular weight DNA and double-stranded RNAs from fungi. *Canadian Journal of Botany* 68: 1898–1902.

Kjærbølling I, Vesth TC, Frisvad JC, Nybo JL, Theobald S, Kuo A, Bowyer P, Matsuda Y, Mondo S, Lyhne EK, *et al.* 2018. Linking secondary metabolites to gene clusters through genome sequencing of six diverse *Aspergillus* species. *Proceedings of the National Academy of Sciences* 115: E753–E761.

Klaubauf S, Zhou M, Lebrun M-H, de Vries RP, Battaglia E. 2016. A novel L-arabinose-responsive regulator discovered in the rice-blast fungus *Pyricularia oryzae* (*Magnaporthe oryzae*). *FEBS letters* 590: 550–558.

Klosterman SJ, Subbarao KV, Kang S, Veronese P, Gold SE, Thomma BPHJ, Chen Z, Henrissat B, Lee Y-H, Park J, *et al.* 2011. Comparative Genomics Yields Insights into Niche Adaptation of Plant Vascular Wilt Pathogens. *PLoS Pathog* 7: e1002137.

Kubicek CP, Herrera-Estrella A, Seidl-Seiboth V, Martinez DA, Druzhinina IS, Thon M, Zeilinger S, Casas-Flores S, Horwitz BA, Mukherjee PK, *et al.* 2011. Comparative genome sequence analysis underscores mycoparasitism as the ancestral life style of Trichoderma. *Genome biology* 12: R40.

Kubicek CP, Steindorff AS, Chenthamara K, Manganiello G, Henrissat B, Zhang J, Cai F, Kopchinskiy AG, Kubicek EM, Kuo A, *et al.* 2019. Evolution and comparative genomics of the most common *Trichoderma* species. *BMC Genomics* 20: 485.

Kumagai T, Ishii T, Terai G, Umemura M, Machida M, Asai K. 2016. Genome Sequence of *Ustilaginoidea virens* IPU010, a Rice Pathogenic Fungus Causing False Smut. *Genome Announcements* 4: e00306-16.

Kumar S, Stecher G, Li M, Knyaz C, Tamura K. 2018. MEGA X: molecular evolutionary genetics analysis across computing platforms. *Molecular Biology and Evolution* 35: 1547–1549.

Lam K-K, LaButti K, Khalak A, Tse D. 2015. FinisherSC: a repeat-aware tool for upgrading *de novo* assembly using long reads. *Bioinformatics* 31: 3207–3209.

Lechner M, Findeiß S, Steiner L, Marz M, Stadler PF, Prohaska SJ. 2011. Proteinortho: Detection of (Co-)orthologs in large-scale analysis. *BMC Bioinformatics* 12: 124.

Letunic I, Bork P. 2018. 20 years of the SMART protein domain annotation resource. *Nucleic Acids Research* 46: D493–D496.

Li W-C, Huang C-H, Chen C-L, Chuang Y-C, Tung S-Y, Wang T-F. 2017. *Trichoderma reesei* complete genome sequence, repeat-induced point mutation, and partitioning of CAZyme gene clusters. *Biotechnology for Biofuels* 10: 170.

Liao Y, Smyth GK, Shi W. 2014. featureCounts: an efficient general purpose program for assigning sequence reads to genomic features. *Bioinformatics* 30: 923–930.

Lombard V, Golaconda Ramulu H, Drula E, Coutinho PM, Henrissat B. 2014. The carbohydrate-active enzymes database (CAZy) in 2013. *Nucleic Acids Research* 42: D490–D495.

Lopez D, Ribeiro S, Label P, Fumanal B, Venisse J-S, Kohler A, de Oliveira RR, Labutti K, Lipzen A, Lail K, *et al.* 2018. Genome-Wide Analysis of *Corynespora cassiicola* Leaf Fall Disease Putative Effectors. *Frontiers in Microbiology* 9.

Love MI, Huber W, Anders S. 2014. Moderated estimation of fold change and dispersion for RNA-seq data with DESeq2. *Genome Biology* 15: 550.

Lücking R, Huhndorf S, Pfister DH, Plata ER, Lumbsch HT. 2009. Fungi evolved right on track. *Mycologia* 101: 810–822.

Ma L-J, van der Does HC, Borkovich KA, Coleman JJ, Daboussi M-J, Di Pietro A, Dufresne M, Freitag M, Grabherr M, Henrissat B, *et al.* 2010. Comparative genomics reveals mobile pathogenicity chromosomes in Fusarium. *Nature* 464: 367–373.

Martin J, Bruno VM, Fang Z, Meng X, Blow M, Zhang T, Sherlock G, Snyder M, Wang Z. 2010a. Rnnotator: an automated *de novo* transcriptome assembly pipeline from stranded RNA-Seq reads. *BMC genomics* 11: 663.

Martin F, Kohler A, Murat C, Balestrini R, Coutinho PM, Jaillon O, Montanini B, Morin E, Noel B, Percudani R, *et al.* 2010b. Périgord black truffle genome uncovers evolutionary origins and mechanisms of symbiosis. *Nature* 464: 1033–1038.

Mchunu NP, Permaul K, Abdul Rahman AY, Saito JA, Singh S, Alam M. 2013. Xylanase Superproducer: Genome Sequence of a Compost-Loving Thermophilic Fungus, *Thermomyces lanuginosus* Strain SSBP. *Genome Announcements* 1: e00388-13.

Mensah MB, Jumpah H, Boadi NO, Awudza JAM. 2021. Assessment of quantities and composition of corn stover in Ghana and their conversion into bioethanol. *Scientific African* 12: e00731.

Mesny F, Miyauchi S, Thiergart T, Pickel B, Atanasova L, Karlsson M, Hüttel B, Barry KW, Haridas S, Chen C, *et al.* 2021. Genetic determinants of endophytism in the *Arabidopsis* root mycobiome. *Nature Communications* 12: 7227.

Mondo SJ, Dannebaum RO, Kuo RC, Louie KB, Bewick AJ, LaButti K, Haridas S, Kuo A, Salamov A, Ahrendt SR, *et al.* 2017. Widespread adenine N6-methylation of active genes in fungi. *Nature Genetics* 49: 964–968.

Moore GG, Mack BM, Beltz SB. 2016. Draft Genome Sequences of Two Closely Related Aflatoxigenic *Aspergillus* Species Obtained from the Ivory Coast. *Genome Biology and Evolution* 8: 729–732.

Morales-Cruz A, Amrine KCH, Blanco-Ulate B, Lawrence DP, Travadon R, Rolshausen PE, Baumgartner K, Cantu D. 2015. Distinctive expansion of gene families associated with plant cell wall degradation, secondary metabolism, and nutrient uptake in the genomes of grapevine trunk pathogens. *BMC Genomics* 16: 469.

Moreno LF, Stielow JB, de Vries M, Weiss VA, Vicente VA, de Hoog S. 2015. Draft Genome Sequence of the Ant-Associated Fungus *Phialophora attae* (CBS 131958). *Genome Announcements* 3: e01099-15.

Müller MC, Praz CR, Sotiropoulos AG, Menardo F, Kunz L, Schudel S, Oberhänsli S, Poretti M, Wehrli A, Bourras S, *et al.* 2019. A chromosome-scale genome assembly reveals a highly dynamic effector repertoire of wheat powdery mildew. *New Phytologist* 221: 2176–2189.

Murat C, Payen T, Noel B, Kuo A, Morin E, Chen J, Kohler A, Krizsán K, Balestrini R, Da Silva C, *et al.* 2018. Pezizomycetes genomes reveal the molecular basis of ectomycorrhizal truffle lifestyle. *Nature Ecology & Evolution* 2: 1956–1965.

Ng KP, Yew SM, Chan CL, Soo-Hoo TS, Na SL, Hassan H, Ngeow YF, Hoh C-C, Lee K-W, Yee W-Y. 2012. Sequencing of *Cladosporium sphaerospermum*, a Dematiaceous fungus isolated from blood culture. *Eukaryotic Cell* 11: 705–706.

Nielsen JC, Grijseels S, Prigent S, Ji B, Dainat J, Nielsen KF, Frisvad JC, Workman M, Nielsen J. 2017. Global analysis of biosynthetic gene clusters reveals vast potential of secondary metabolite production in *Penicillium* species. *Nature Microbiology* 2: 1–9.

Nierman WC, Fedorova-Abrams ND, Andrianopoulos A. 2015. Genome Sequence of the AIDS-Associated *Pathogen Penicillium marneffei* (ATCC18224) and Its Near Taxonomic Relative *Talaromyces stipitatus* (ATCC10500). *Genome Announcements* 3: e01559-14.

O'Connell RJ, Thon MR, Hacquard S, Amyotte SG, Kleemann J, Torres MF, Damm U, Buiate EA, Epstein L, Alkan N, *et al.* 2012. Lifestyle transitions in plant pathogenic *Colletotrichum* fungi deciphered by genome and transcriptome analyses. *Nature Genetics* 44: 1060–1065.

Ohm RA, Feau N, Henrissat B, Schoch CL, Horwitz BA, Barry KW, Condon BJ, Copeland AC, Dhillon B, Glaser F, *et al.* 2012. Diverse Lifestyles and Strategies of Plant Pathogenesis Encoded in the Genomes of Eighteen *Dothideomycetes* Fungi (A Andrianopoulos, Ed.). *PLoS Pathogens* 8: e1003037.

Peng M, Dilokpimol A, Mäkelä MR, Hildén K, Bervoets S, Riley R, Grigoriev IV, Hainaut M, Henrissat B, de Vries RP, *et al.* 2017. The draft genome sequence of the ascomycete fungus *Penicillium subrubescens* reveals a highly enriched content of plant biomass related CAZymes compared to related fungi. *Journal of Biotechnology* 246: 1–3.

Peter M, Kohler A, Ohm RA, Kuo A, Krützmann J, Morin E, Arend M, Barry KW, Binder M, Choi C, *et al.* 2016. Ectomycorrhizal ecology is imprinted in the genome of the dominant symbiotic fungus *Cenococcum geophilum*. *Nature Communications* 7: 12662.

Petersen TN, Brunak S, von Heijne G, Nielsen H. 2011. SignalP 4.0: discriminating signal peptides from transmembrane regions. *Nature Methods* 8: 785–786.

Rawlings ND, Barrett AJ, Bateman A. 2012. MEROPS: the database of proteolytic enzymes, their substrates and inhibitors. *Nucleic Acids Research* 40: D343–D350.

Schardl CL, Young CA, Hesse U, Amyotte SG, Andreeva K, Calie PJ, Fleetwood DJ, Haws DC, Moore N, Oeser B, *et al.* 2013. Plant-symbiotic fungi as chemical engineers: multi-genome analysis of the Clavicipitaceae reveals dynamics of alkaloid loci (J Heitman, Ed.). *PLoS Genetics* 9: e1003323.

Schmidt AR, Beimforde C, Seyfullah LJ, Wege S-E, Dörfelt H, Girard V, Grabenhorst H, Gube M, Heinrichs J, Nel A, *et al.* 2014. Amber fossils of sooty moulds. *Review of Palaeobotany and Palynology* 200: 53–64.

Sharpton TJ, Stajich JE, Rounsley SD, Gardner MJ, Wortman JR, Jordar VS, Maiti R, Kodira CD, Neafsey DE, Zeng Q, *et al.* 2009. Comparative genomic analyses of the human fungal pathogens *Coccidioides* and their relatives. *Genome Research* 19: 1722–1731.

Shu R, Meng Q, Zhang H, Zhou G, Li M, Wu P, Zhao Y, Chen C, Qin Q. 2020. A New High-Quality Draft Genome Assembly of the Chinese Cordyceps *Ophiocordyceps sinensis*. *Genome Biology and Evolution* 12: 1074–1079.

Sonnhammer EL, Eddy SR, Durbin R. 1997. Pfam: a comprehensive database of protein domain families based on seed alignments. *Proteins* 28: 405–420.

Staats M, van Kan JAL. 2012. Genome update of *Botrytis cinerea* strains B05.10 and T4. *Eukaryotic Cell* 11: 1413–1414.

Sung G-H, Poinar GO, Spatafora JW. 2008. The oldest fossil evidence of animal parasitism by fungi supports a Cretaceous diversification of fungal–arthropod symbioses. *Molecular Phylogenetics and Evolution* 49: 495–502.

Tamura K, Battistuzzi FU, Billing-Ross P, Murillo O, Filipski A, Kumar S. 2012. Estimating divergence times in large molecular phylogenies. *Proceedings of the National Academy of Sciences of the United States of America* 109: 19333–19338.

Tamura K, Tao Q, Kumar S. 2018. Theoretical foundation of the RelTime method for estimating divergence times from variable Eevolutionary rates. *Molecular Biology and Evolution* 35: 1770–1782.

Tao Q, Tamura K, Mello B, Kumar S. 2020. Reliable confidence intervals for RelTime estimates of evolutionary divergence times. *Molecular Biology and Evolution* 37: 280–290.

Taylor TN, Hass H, Kerp H. 1999. The oldest fossil ascomycetes. *Nature* 399: 648.

Taylor TN, Hass H, Kerp H, Krings M, Hanlin RT. 2005. Perithecial ascomycetes from the 400 million year old Rhynie chert: an example of ancestral polymorphism. *Mycologia* 97: 269–285.

Teixeira MM, Moreno LF, Stielow BJ, Muszewska A, Hainaut M, Gonzaga L, Abouelleil A, Patané JSL, Priest M, Souza R, *et al.* 2017. Exploring the genomic diversity of black yeasts and relatives (*Chaetothyriales, Ascomycota*). *Studies in Mycology* 86: 1–28.

Terfehr D, Dahlmann TA, Specht T, Zadra I, Kürnsteiner H, Kück U. 2014. Genome Sequence and Annotation of *Acremonium chrysogenum*, Producer of the β-Lactam Antibiotic Cephalosporin C. *Genome Announcements* 2: e00948-14.

Tomaszewska J, Bieliński D, Binczarski M, Berlowska J, Dziugan P, Piotrowski J, Stanishevsky A, Witońska IA. 2018. Products of sugar beet processing as raw materials for chemicals and biodegradable polymers. *RSC Advances* 8: 3161–3177.

Traeger S, Altegoer F, Freitag M, Gabaldon T, Kempken F, Kumar A, Marcet-Houben M, Pöggeler S, Stajich JE, Nowrousian M. 2013. The Genome and Development-Dependent Transcriptomes of *Pyronema confluens*: A Window into Fungal Evolution. *PLOS Genetics* 9: e1003820.

Urquhart AS, Mondo SJ, Mäkelä MR, Hane JK, Wiebenga A, He G, Mihaltcheva S, Pangilinan J, Lipzen A, Barry K, *et al.* 2018. Genomic and Genetic Insights Into a Cosmopolitan Fungus, *Paecilomyces variotii* (Eurotiales). *Frontiers in Microbiology* 9.

Vandeputte P, Ghamrawi S, Rechenmann M, Iltis A, Giraud S, Fleury M, Thornton C, Delhaès L, Meyer W, Papon N, *et al.* 2014. Draft Genome Sequence of the Pathogenic Fungus *Scedosporium apiospermum*. *Genome Announcements* 2: e00988-14.

Verma S, Gazara RK, Nizam S, Parween S, Chattopadhyay D, Verma PK. 2016. Draft genome sequencing and secretome analysis of fungal phytopathogen *Ascochyta rabiei* provides insight into the necrotrophic effector repertoire. *Scientific Reports* 6: 24638.

Vesth TC, Nybo JL, Theobald S, Frisvad JC, Larsen TO, Nielsen KF, Hoof JB, Brandl J, Salamov A, Riley R, *et al.* 2018. Investigation of inter- and intraspecies variation through genome sequencing of *Aspergillus* section Nigri. *Nature Genetics* 50: 1688–1695.

Vries RP de, Burgers K, Vondervoort PJI van de, Frisvad JC, Samson RA, Visser J. 2004. A new black *Aspergillus* species, *A. vadensis*, is a promising host for homologous and heterologous protein production. *Applied and Environmental Microbiology* 70: 3954–3959.

de Vries RP, Riley R, Wiebenga A, Aguilar-Osorio G, Amillis S, Uchima CA, Anderluh G, Asadollahi M, Askin M, Barry K, *et al.* 2017. Comparative genomics reveals high biological diversity and specific adaptations in the industrially and medically important fungal genus *Aspergillus*. *Genome Biology* 18: 28.

Walker AK, Frasz SL, Seifert KA, Miller JD, Mondo SJ, LaButti K, Lipzen A, Dockter RB, Kennedy MC, Grigoriev IV, *et al.* 2016. Full Genome of *Phialocephala scopiformis* DAOMC 229536, a Fungal Endophyte of Spruce Producing the Potent Anti-Insectan Compound Rugulosin. *Genome Announcements* 4: e01768-15.

Waterhouse RM, Seppey M, Simão FA, Manni M, Ioannidis P, Klioutchnikov G, Kriventseva EV, Zdobnov EM. 2018. BUSCO applications from quality assessments to gene prediction and phylogenomics. *Molecular Biology and Evolution* 35: 543–548.

Wingfield BD, Barnes I, de Beer ZW, De Vos L, Duong TA, Kanzi AM, Naidoo K, Nguyen HDT, Santana QC, Sayari M, *et al.* 2015. Draft genome sequences of *Ceratocystis eucalypticola,*

Chrysoporthe cubensis, C. deuterocubensis, Davidsoniella virescens, Fusarium temperatum, Graphilbum fragrans, Penicillium nordicum, and *Thielaviopsis musarum*. *IMA Fungus* 6: 493–506.

Woźniak M, Ratajczak I, Wojcieszak D, Waśkiewicz A, Szentner K, Przybył J, Borysiak S, Goliński P. 2021. Chemical and Structural Characterization of Maize Stover Fractions in Aspect of Its Possible Applications. *Materials* 14: 1527.

Wu W, Davis RW, Tran-Gyamfi MB, Kuo A, LaButti K, Mihaltcheva S, Hundley H, Chovatia M, Lindquist E, Barry K, *et al.* 2017. Characterization of four endophytic fungi as potential consolidated bioprocessing hosts for conversion of lignocellulose into advanced biofuels. *Applied Microbiology and Biotechnology* 101: 2603–2618.

Xiao G, Ying S-H, Zheng P, Wang Z-L, Zhang S, Xie X-Q, Shang Y, St Leger RJ, Zhao G-P, Wang C, *et al.* 2012. Genomic perspectives on the evolution of fungal entomopathogenicity in *Beauveria bassiana*. *Scientific Reports* 2: 483.

Yang J, Wang L, Ji X, Feng Y, Li X, Zou C, Xu J, Ren Y, Mi Q, Wu J, *et al.* 2011. Genomic and proteomic analyses of the fungus *Arthrobotrys oligospora* provide insights into nematode-trap formation. *PLoS pathogens* 7: e1002179.

Zampounis A, Pigné S, Dallery J-F, Wittenberg AHJ, Zhou S, Schwartz DC, Thon MR, O'Connell RJ. 2016. Genome Sequence and Annotation of *Colletotrichum higginsianum*, a Causal Agent of Crucifer Anthracnose Disease. *Genome Announcements* 4.

Zeiner CA, Purvine SO, Zink EM, Paša-Tolić L, Chaput DL, Haridas S, Wu S, LaButti K, Grigoriev IV, Henrissat B, *et al.* 2016. Comparative Analysis of Secretome Profiles of Manganese(II)-Oxidizing Ascomycete Fungi. *PLOS ONE* 11: e0157844.

Zerbino DR, Birney E. 2008. Velvet: Algorithms for de novo short read assembly using de Bruijn graphs. *Genome Research* 18: 821–829.

Zhu S, Cao Y-Z, Jiang C, Tan B-Y, Wang Z, Feng S, Zhang L, Su X-H, Brejova B, Vinar T, *et al.* 2012. Sequencing the genome of *Marssonina brunnea* reveals fungus-poplar co-evolution. *BMC Genomics* 13: 382.

Zuckerkandl E, Pauling L. 1965. Evolutionary divergence and convergence in proteins. In: Bryson V, Vogel HJ, eds. Evolving Genes and Proteins. Academic Press, 97–166.
