# Supplementary figures and images for: Genome evolution and transcriptome plasticity is associated with adaptation to monocot and dicot plants in Colletotrichum fungi
Source: Gigascience. 2024 Jun 28;13:giae036. doi: 10.1093/gigascience/giae036 (PMC11212070; doi:10.1093/gigascience/giae036)

C. higginsianum

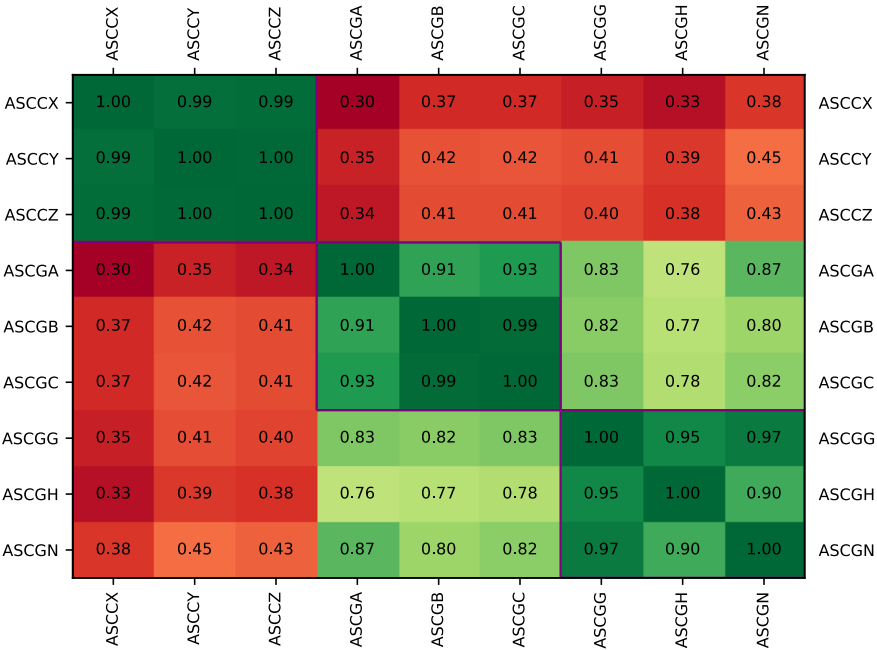

C. graminicola

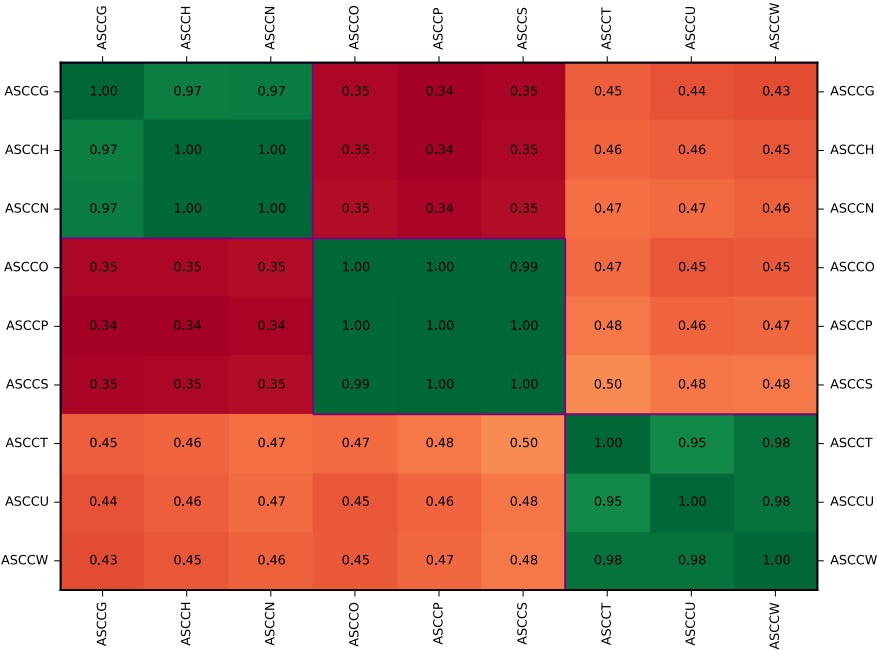

C. phormii

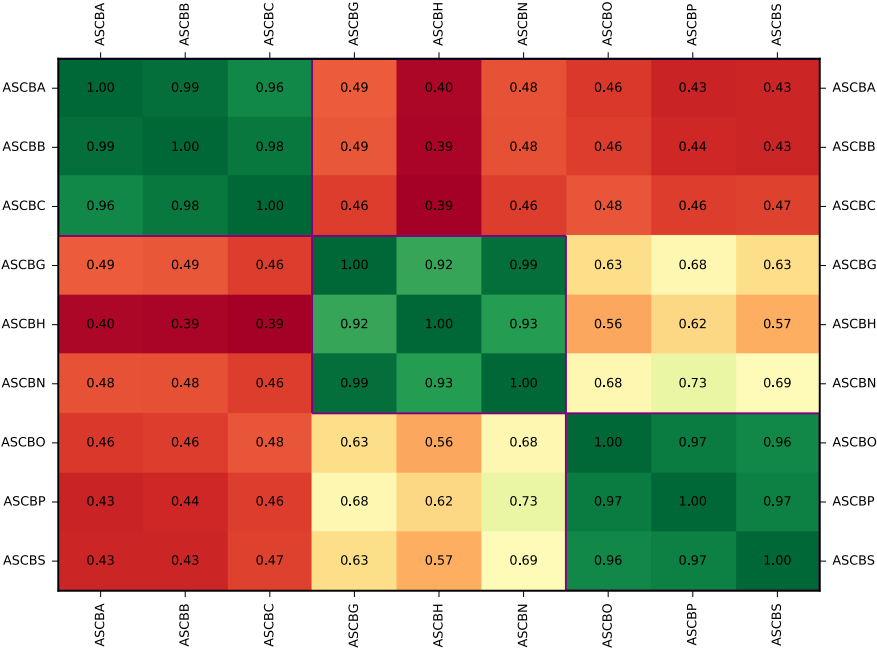

C. nymphaeae

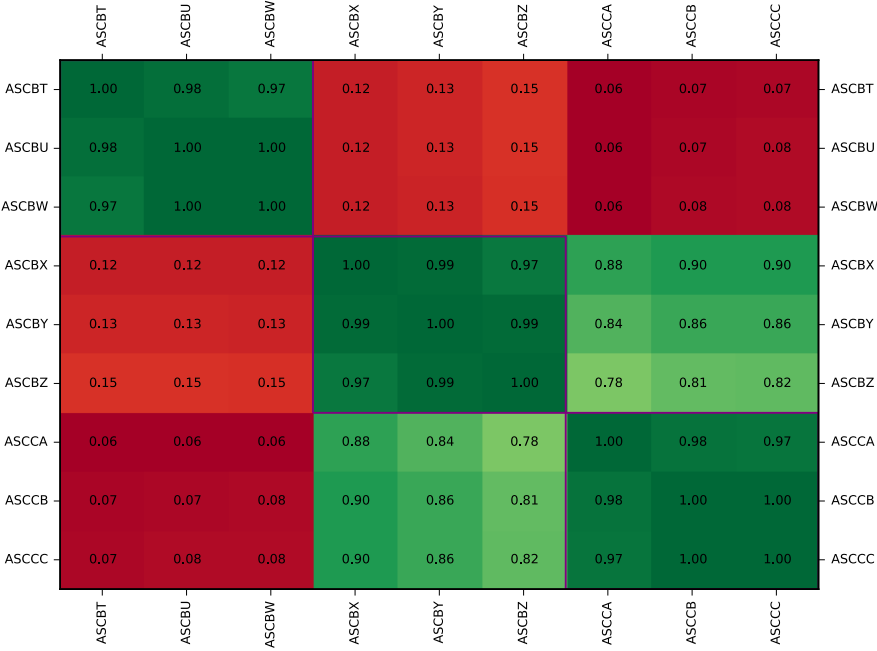

Supplement: giae036_Supplemental_Figures_and_Tables [file giae036_supplemental_figures_and_tables.zip › Supplementary Figure S4 - RNA replicates heatmaps.pdf]

glucose vs MS

glucose vs DS

MS vs DS

*C. higginsianum*

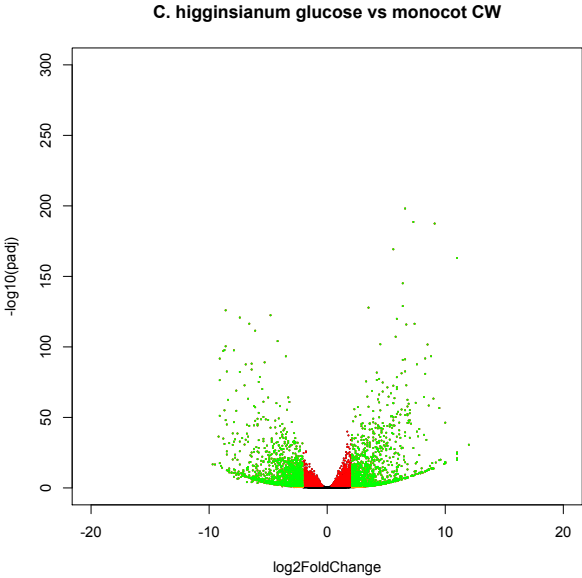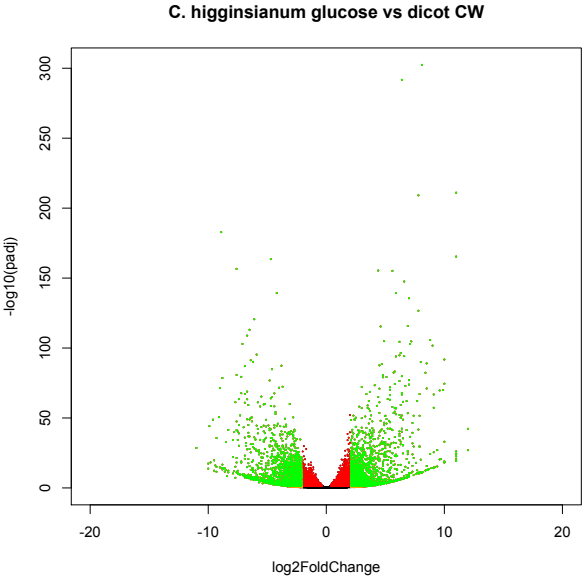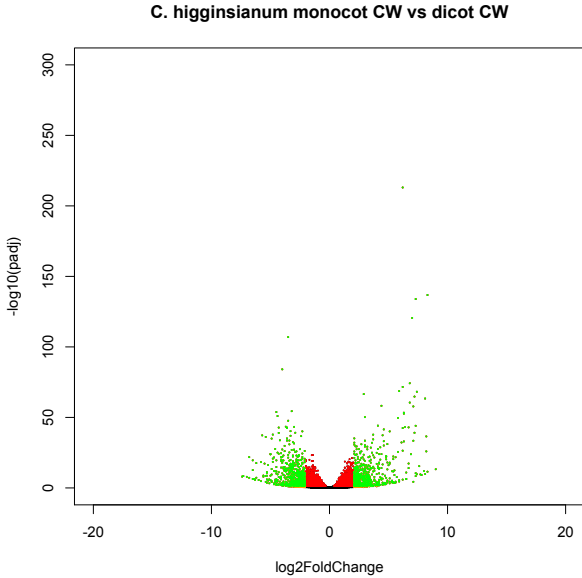

*C. graminicola*

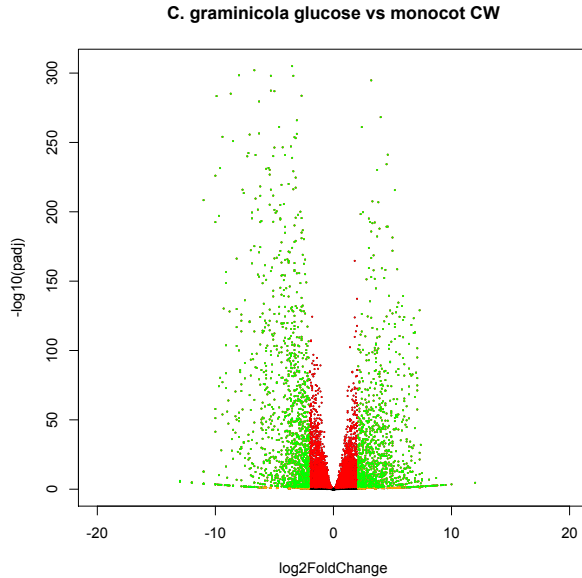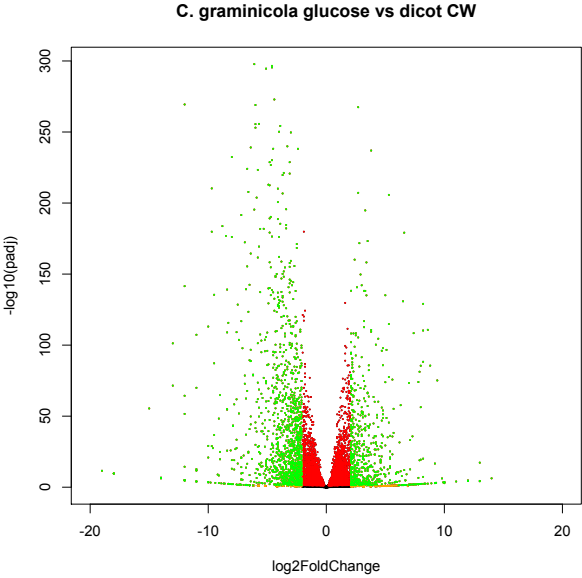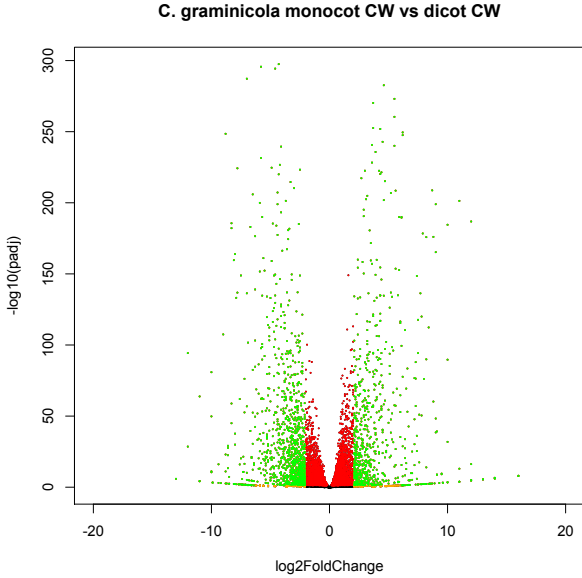

*C. phormii*

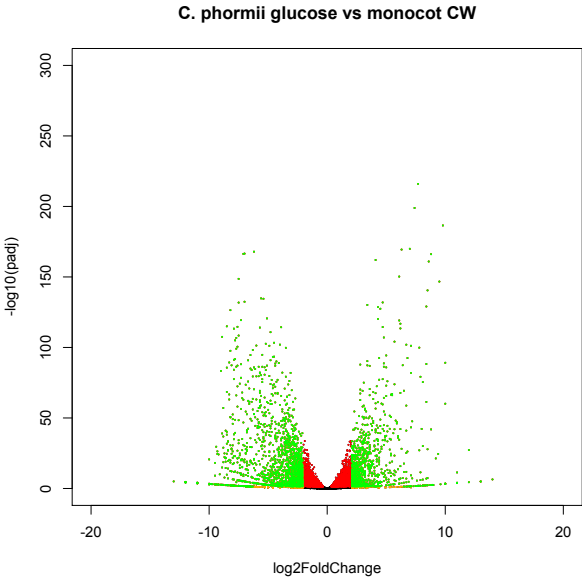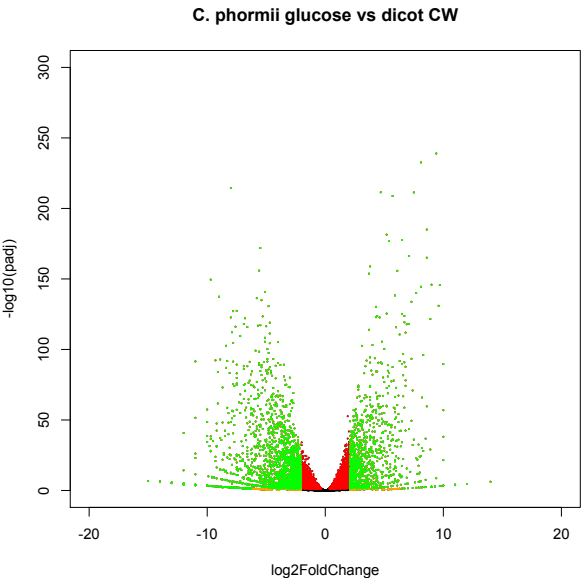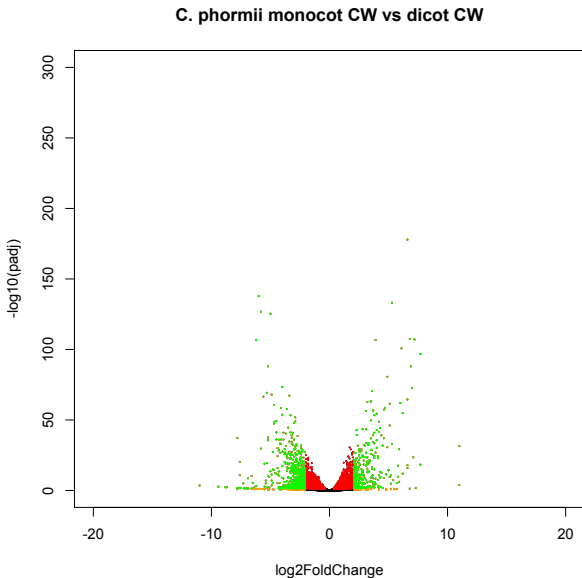

*C. nymphaeae*

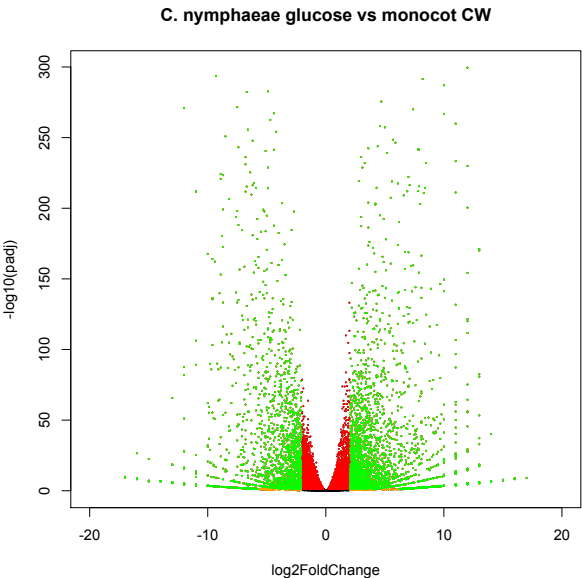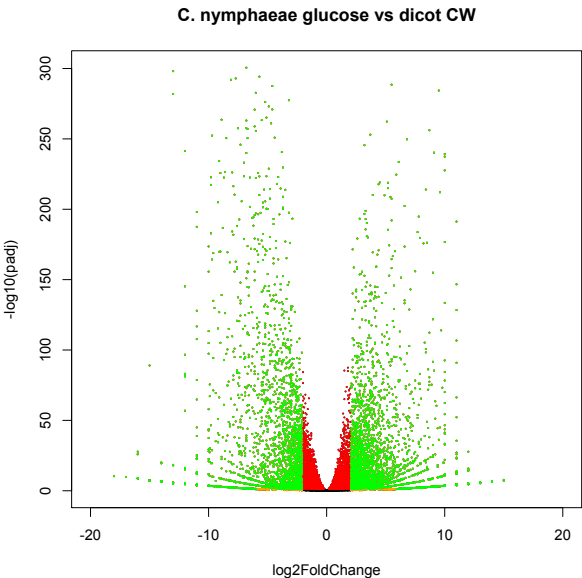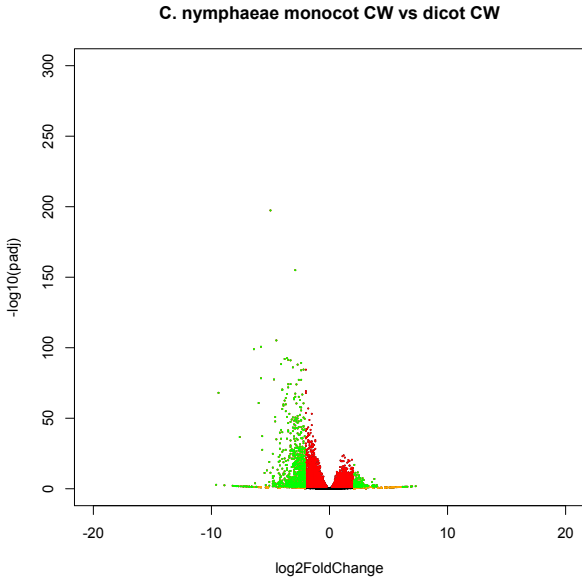

Supplement: giae036_Supplemental_Figures_and_Tables [file giae036_supplemental_figures_and_tables.zip › Supplementary Figure S5 - DEGs Vulcano plots.pdf]
