# Supplementary material for: Genome evolution and transcriptome plasticity is associated with adaptation to monocot and dicot plants in Colletotrichum fungi
Source: Gigascience. 2024 Jun 28;13:giae036. doi: 10.1093/gigascience/giae036 (PMC11212070; doi:10.1093/gigascience/giae036)
Supplement: giae036_Response_to_Reviewer_Comments_Original_Submission [file giae036_response_to_reviewer_comments_original_submission.pdf]

Author's Response To Reviewer Comments

GIGA-D-23-00216
Genome evolution and transcriptome plasticity is associated with adaptation to monocot and dicot plants in Colletotrichum fungi.
Riccardo Baroncelli; José F. Cobo-Díaz; Tiziano Benocci; Mao Peng; Evy Battaglia; Sajeet Haridas; William Andreopoulos; Kurt LaButti; Jasmyn Pangilinan; Anna Lipzen; Maxim Koriabine; Diane Bauer; Gaetan Le Floch; Miia R. Mäkelä; Elodie Drula; Bernard Henrissat; Igor V. Grigoriev; Jo Anne Crouch; Ronald P. de Vries; Serenella A. Sukno; Michael R. Thon
GigaScience

Dear prof. Baroncelli,

Your manuscript "Genome evolution and transcriptome plasticity associated with adaptation to monocot and dicot plants in Colletotrichum fungi." (GIGA-D-23-00216) has been assessed by our reviewers. Based on these reports, and my own assessment as Editor, I am pleased to inform you that it is potentially acceptable for publication in GigaScience, once you have carried out some essential revisions suggested by our reviewers.

Their reports, together with any other comments, are below. Please also take a moment to check our website at https://www.editorialmanager.com/giga/ for any additional comments that were saved as attachments.

In addition, please register any new software application in the bio.tools and SciCrunch.org databases to receive RRID (Research Resource Identification Initiative ID) and biotoolsID identifiers, and include these in your manuscript. Computational workflows should be registered in workflowhub.eu and the DOIs cited in the relevant places in the manuscript. These will facilitate tracking, reproducibility and re-use of your tool.

Once you have made the necessary corrections, please submit a revised manuscript online at:

https://www.editorialmanager.com/giga/

If you have forgotten your username or password please use the "Send Login Details" link to get your login information. For security reasons, your password will be reset.

Please include a point-by-point within the 'Response to Reviewers' box in the submission system. Please ensure you describe additional experiments that were carried out and include a detailed rebuttal of any criticisms or requested revisions that you disagreed with. Please also ensure that your revised manuscript conforms to the journal style, which can be found in the Instructions for Authors on the journal homepage. If the data and code has been modified in the revision process please be sure to update the public versions of this too.

The due date for submitting the revised version of your article is 30 Mar 2024.

We look forward to receiving your revised manuscript soon.

Best wishes,

Hongfang Zhang
GigaScience
www.gigasciencejournal.com

Reviewer reports:
Reviewer #1: In this study, Baroncelli and colleagues carry out a comprehensive analysis of genomic

evolution in Colletotrichum fungi, an important group of plant pathogens with diverse and economically significant hosts. Their comparative genomic and phylogenomics analyses are based on the genome sequences of 30 Colletotrichum species spanning the diversity of the genus, including pathogens of dicots, monocots, and both dicots and monocots. This includes 18 genome sequences that are newly reported in this study. They also perform comparative transcriptomic analyses of 4 Colletotrichum species (2 dicot pathogens and 2 monocot pathogens) on different carbon sources.

Overall, I thought the manuscript was very well written and technically sound. The results should be of interest to a broad audience, particularly to those interested in fungal evolutionary genomics and plant pathology. I only have a few minor comments.

Minor comments:
(1) Lines 50 - 51: "The plant cell wall (PCW) consists of many different polysaccharides that are attached not only to each other through a variety of linkages providing the main strength and structure for the PCW".
I found this confusing - is the sentence incomplete?
RESPONSE:
The sentence has been reshaped to clarify.

(2) Line 66: "Some Colletotrichum species show…"
I think there should be a couple of introductory sentences about Colletotrichum before this.
RESPONSE:
A paragraph to introduce Colletotrichum has been added to the text as suggested

(3) Figure 1: It would be informative to label which genomes were sequenced with PacBio versus just Illumina.
RESPONSE:
Information related to the sequencing technologies used (short or long reads) for each genome have been added in Figure 1

(4) Lines 254 - 255: "As no other enrichment was identified we performed a manual annotation of genes identified in Figure 3D".
I don't think it is clear here what manual annotation this is referring to.
RESPONSE:
We used an integrated approach combining the results of InterProScan and Blast on each selected protein to predict the biological function of the selected proteins. The sentence has been changed to clarify.

(5) One area where I felt the analysis was lacking was the lack of analyses on genome repeat content. The authors highlight the large variation in genome sizes within Colletotrichum species (~44 Mb vs ~90 Mb) and show in Figure 1 that this correlates with increased non-coding DNA. It would have been interesting to determine if this is driven by the proliferation of particular repeat families.
RESPONSE:
Thanks for the useful comment. The authors have performed more analyses to characterize the content of repetitive elements (RE) in the set of genomes analysed. Figure 1 has been implemented with the new results to show highlight the link between genome features and proliferation of RE. A summary of repetitive sequences can also be found in supplementary table S2.

(6) Another concern is the inconsistent use of genome annotation methods. 12 of the genomes reported in this study were annotated using the JGI annotation pipeline, whereas the other 6 were annotated using the MAKER pipeline. Several studies (e.g., Weisman et al., 2022 - Current Biology) show that inconsistent genome annotation methods can inflate the number of observed lineage specific genes. The authors may wish to comment on this or demonstrate that this isn't an issue in their study (e.g., by aligning lineage specific proteins against the other genome assemblies).
RESPONSE:
We do agree with the reviewer as different genome annotation methods can affect the number of genes, especially the lineage specific ones. In that context, when we started to collaborate with the JGI, we have set up the MAKER pipeline based on the JGI approach and using several genomes annotated by the JGI to

make sure the protocols were as similar as possible (e.g. using precompiled AUGUSTUS models based on EST sequences, and using either the EST or alt_EST (EST of closely related species) parameters for all species sequenced). We reannotated the genome sequence of C. eremechloae and C. phormii (also based on comments of reviewer 2), using our MAKER pipeline to show the consistency with the original annotation made by the JGI. For Colletotrichum phormii, MAKER predicted 15177 protein-coding genes while the JGI pipeline predicted 15209 (+32) protein-coding genes. For Colletotrichum eremechloae, MAKER predicted 15185 protein-coding genes while the JGI pipeline predicted 15169 (+16) protein-coding genes. Both ggf3 files are available in GIGA db.

Reviewer #2: This manuscript describes the adaptation of the Colletotrichum genus to monocotyledonous and dicotyledonous plants with regard to the content and expression of genes from 30 genomes, with a sub-sampling of 4 genomes for transcriptomic analyses.

Major remarks:

"Considering that the analyses carried out are affected by the sampling, as closely related species are likely to have more shared genes compared to species that are more distant from others," Yes, Indeed, it's clearly a possible bias due to the sampling, as you write. As you considered all genomes together to define specific genes, monocot specific species have few specific genes due to their phylogenetic proximity. Based on this, could you address these observations based on combination of figure 1 and 2:
1. The number of specific genes in C.eremochloae (1608) vs in C.sublineola (1643), while divergence time between both seems short and similar to the group of C.lupini, C.costaricense (monoct) … with approximatively 100 genes specific to each species. How could such closely related genomes have acquired so many specific genes in such a short time compared with other species during the same period of evolution?
RESPONSE:
We compared the lineage specific genes from C. eremochloae to C. sublineola and vice versa and also did the same with C. phormii and C. salicis. We found that most of the lineage specific genes have homologous sequences in the other genome but have deletions and/or nucleotide substitutions. We hypothesize that the lineage specific genes are actually due to gene losses in the other species. We have updated the text to reflect this.

2. Same remark for in C.phormii (911) vs C.salicis (286), when it's even more disturbing with the switch to dicot and a loss of many genes for C.salicis.
For both cases mentioned above, a detailed comparison between the two genomes could be useful to obtain some explanations of the events and genes involved.
Finally, one of the most interesting thing is the proximity of C. phormii and C.salicis in the same clade but with a recent host specialization. Despite the poor quality of the genome of C.salicis vs C.phormii, an comparative genomic approach with a tool like Synchro could provide clues as to gene losses and their location (all along the genome/ specific regions).
RESPONSE:
We tried to do an analysis of synteny but the fragmented nature of some of the genomes prevented any firm conclusions. We also looked at their distribution in the genome but found no evidence of clustering; they seem to be randomly distributed in the genomes. Please see our response to the previous comment for more information about the lineage specific genes.

Moreover, interpretation of the phylogenetic tree (Figure 1), could be lead to propose three clusters of genomes, based on evolution time and plant host: Monocot, Dicot "old" (C.orbiculare, C.noveboracense, …) and Dicot "young" (C.melonis, C.cuscutae, …). Did the authors attempt an analysis with a such view of the data? Maybe that will complete the view of C.acutatum complex (46 genes) vs C.graminciola complex (28 genes) form which C.orchidophylum and C.phormis are excluded.
RESPONSE:
This could be an interesting way to interpret the tree. The problem is that we only have three species in the ‘old dicot’ group and among these, C. orbiculare is a bit odd with respect to its genome size and C. chlorophyti is associated with both monocots and dicots. In our opinion, a larger sampling of these older clades would be needed to make a meaningful distinction between old and no dicot pathogens. Moreover,

we believe that the number of clusters identified may be affected by the genome sampling as increasing the number of genomes in the analyses may also increase the number of clusters identified.

Figure 3: Please explain further Figure 3 A, described as a PCA. No axis (dimension) has been shown with a % explaining the divergence between organisms. This is confusing and does not allow me to know whether the gene sets used to compare the 4 genomes are only shared genes or all genes. The rest of the figure is much clearer and the comments are clear on the response to species specificity (under/over expression of genes) for each genome.
RESPONSE:
The PCA in figure 3 has been updated and now reports in the axis the % of significance, the legend has been changed to clarify the input and an extra supplementary file (Suppl 16) has been added.

Figure 4: "the expression of the orthologous genes was clustered for the four fungal species (Figure 4A)" As written, it is assumed that you used ortholog genes established between the 4 species, this does not appear to be the case with so many genes missing in C.graminicola in figure 4. To continue on this point, I have not found the minimum number of species found in a cluster to set a cluster of orthologs (maybe written but not found). What is the threshold for divergence or sequence similarity? Have you considered sequence length (query coverage vs subject coverage) to allow clustering of potentially split/fragmented genes in annotations?
RESPONSE:
We performed Proteinortho on the four species used in the transcriptomics analyses (evalue: 1e-05; percent identity of best blast hits: 25%; min. coverage of best blast alignments: 50%.) using the synteny of the genomes as input but excluding the singleton genes. We then combine the output of Proteinortho with expression data using as cutoff: log2FoldChange > 2 and p-value < 0.05; the text has been improved based on the reviewer suggestion. In figure 4: white cells indicate gene losses; indeed C. graminicola has a shrink set of genes compared to closely related species and this evidence is also reflected in the number of CAZy encoding genes.

Minor remarks:

The authors limit their analysis to 30 genomes, whereas more than 270 genomes of Colletotrichum are available, from over 70 species. Research time is clearly longer than the time to generate genomic resources, but it could be interesting to list a few new genomes missing from those analysed and that could have significant added value (particularly if sequenced in long reads, providing complete genomes).

Transcriptomic analyses were carried out on 4 genomes. The choice of the genomes was not discussed, and maybe done by convenience with strains available at the lab. In fact, C.higginsianum is well sequenced, assembled and studied and chosen as one of the specific hosts of dicotyledons, whereas it is a member of the C.destructivum complex. Similarly, C.phormii appears to be a recent species with an adaptation to monocots.
RESPONSE:
Exactly! The choice of the isolates selected for the transcriptomic approach was mainly based on evolutionary history of association with the host.
We wanted to selected mono and dicot pathogens with different evolutionary histories and specifically: 1) species that have a long history of host association with mono (like C. graminicola) and 2) species that had only “recently” adapted to mono (like C. phormi).
In the first case we selected as “sister species” C. graminicola and C. higginsianum (both species are model systems and have plant transcriptomic data for future comparison); in the second case we selected C. nymphaeae, which, like C. phormii, is a member of the Acutatum specie complex and also based on the economic impact of this species, to improve the resolution and the focus of the experiment. The text has been improved based on the reviewer’s comment.

113 : "species with bigger genomes are characterized by a lower GC content", please rewrite the link between genome size and GC content. Between species of same genus genome size is most often linked to the invasion of TE element (RIPed or not in fungi). Strongly ripped genomes (Leptosphaeria, Venturia) are not always large compared to the size of other species.
RESPONSE:

Based on this comment and comments done by the first reviewer we performed more analyses to characterize the content of repetitive elements (RE) in the set of genomes analysed. The text has also been changed to improve clarity.

Data availability. All genomes were released in public Databases. I do not find accession numbers for RNA-Seq runs.
RESPONSE:
These accession numbers have been added to the manuscript in supplementary files (Supplementary Table S13).

Many supplementary details have been provided. I appreciate the BUSCO logs for checking the completeness of gene sets, which provide me some clues about the quality of genome annotation, that was never discussed or pointed out in the manuscript as possible source of bias.
RESPONSE:
We do agree that assessing the quality of the genome is essential to avoid bias in comparative genomics analyses; a sentence has been added in the text to point it out.

Overall, the manuscript is very interesting and confirms the results previously identified in terms of specificities of CAZy families associated with host plant adaptation in the Colletotrichum genus. The authors demonstrate a great knowledge of the CAZome and associated biological processes, which provides a great deal of valuable information for the community working on Colletotrichum and more generally for all those working on such enzymes. Finally, the transcriptomic data suggest that species specificity and host adaptation are more related to an expression pattern than to specific gene content, than a specific gene content.
