# Supplementary material for: Genome evolution and transcriptome plasticity is associated with adaptation to monocot and dicot plants in Colletotrichum fungi
Source: Gigascience. 2024 Jun 28;13:giae036. doi: 10.1093/gigascience/giae036 (PMC11212070; doi:10.1093/gigascience/giae036)
Supplement: giae036_Reviewer_1_Report_Original_Submission — Jamie McGowan -- 12/17/2023 Reviewed [file giae036_reviewer_1_report_original_submission.pdf]

Reviewer Report

Title: Genome evolution and transcriptome plasticity is associated with adaptation to monocot and dicot plants in Colletotrichum fungi.

Version: Original Submission **Date:** 12/17/2023

Reviewer name: Jamie McGowan

Reviewer Comments to Author:

In this study, Baroncelli and colleagues carry out a comprehensive analysis of genomic evolution in Colletotrichum fungi, an important group of plant pathogens with diverse and economically significant hosts. Their comparative genomic and phylogenomics analyses are based on the genome sequences of 30 Colletotrichum species spanning the diversity of the genus, including pathogens of dicots, monocots, and both dicots and monocots. This includes 18 genome sequences that are newly reported in this study. They also perform comparative transcriptomic analyses of 4 Colletotrichum species (2 dicot pathogens and 2 monocot pathogens) on different carbon sources.

Overall, I thought the manuscript was very well written and technically sound. The results should be of interest to a broad audience, particularly to those interested in fungal evolutionary genomics and plant pathology. I only have a few minor comments.

Minor comments:

(1) Lines 50 - 51: "The plant cell wall (PCW) consists of many different polysaccharides that are attached not only to each other through a variety of linkages providing the main strength and structure for the PCW".

I found this confusing - is the sentence incomplete?

(2) Line 66: "Some Colletotrichum species show…"

I think there should be a couple of introductory sentences about Colletotrichum before this.

(3) Figure 1: It would be informative to label which genomes were sequenced with PacBio versus just Illumina.

(4) Lines 254 - 255: "As no other enrichment was identified we performed a manual annotation of genes identified in Figure 3D".

I don't think it is clear here what manual annotation this is referring to.

(5) One area where I felt the analysis was lacking was the lack of analyses on genome repeat content. The authors highlight the large variation in genome sizes within Colletotrichum species (~44 Mb vs ~90 Mb) and show in Figure 1 that this correlates with increased non-coding DNA. It would have been interesting to determine if this is driven by the proliferation of particular repeat families.

(6) Another concern is the inconsistent use of genome annotation methods. 12 of the genomes reported in this study were annotated using the JGI annotation pipeline, whereas the other 6 were annotated using the MAKER pipeline. Several studies (e.g., Weisman et al., 2022 - Current Biology) show that inconsistent genome annotation methods can inflate the number of observed lineage specific genes. The authors may wish to comment on this or demonstrate that this isn't an issue in their study (e.g., by

aligning lineage specific proteins against the other genome assemblies).

Methods

Are the methods appropriate to the aims of the study, are they well described, and are necessary controls included? Choose an item.

Conclusions

Are the conclusions adequately supported by the data shown? Choose an item.

Reporting Standards

Does the manuscript adhere to the journal's guidelines on minimum standards of reporting? Choose an item.

Choose an item.

Statistics

Are you able to assess all statistics in the manuscript, including the appropriateness of statistical tests used? Choose an item.

Quality of Written English

Please indicate the quality of language in the manuscript: Choose an item.

Declaration of Competing Interests

Please complete a declaration of competing interests, considering the following questions:

- Have you in the past five years received reimbursements, fees, funding, or salary from an organisation that may in any way gain or lose financially from the publication of this manuscript, either now or in the future?
- Do you hold any stocks or shares in an organisation that may in any way gain or lose financially from the publication of this manuscript, either now or in the future?
- Do you hold or are you currently applying for any patents relating to the content of the manuscript?
- Have you received reimbursements, fees, funding, or salary from an organization that holds or has applied for patents relating to the content of the manuscript?
- Do you have any other financial competing interests?
- Do you have any non-financial competing interests in relation to this paper?

If you can answer no to all of the above, write 'I declare that I have no competing interests' below. If your reply is yes to any, please give details below.

I declare that I have no competing interests.

I agree to the open peer review policy of the journal. I understand that my name will be included on my report to the authors and, if the manuscript is accepted for publication, my named report including any attachments I upload will be posted on the website along with the authors' responses. I agree for my report to be made available under an Open Access Creative Commons CC-BY license (http://creativecommons.org/licenses/by/4.0/). I understand that any comments which I do not wish to be included in my named report can be included as confidential comments to the editors, which will not be published.

Choose an item.

To further support our reviewers, we have joined with Publons, where you can gain additional credit to further highlight your hard work (see: https://publons.com/journal/530/gigascience). On publication of this paper, your review will be automatically added to Publons, you can then choose whether or not to claim your Publons credit. I understand this statement.

Yes Choose an item.
