# Supplementary material for: Genome evolution and transcriptome plasticity is associated with adaptation to monocot and dicot plants in Colletotrichum fungi
Source: Gigascience. 2024 Jun 28;13:giae036. doi: 10.1093/gigascience/giae036 (PMC11212070; doi:10.1093/gigascience/giae036)
Supplement: giae036_Reviewer_2_Report_Original_Submission — Nicolas Lapalu -- 12/22/2023 Reviewed [file giae036_reviewer_2_report_original_submission.pdf]

Reviewer Report

Title: Genome evolution and transcriptome plasticity is associated with adaptation to monocot and dicot plants in Colletotrichum fungi.

Version: Original Submission **Date:** 12/22/2023

Reviewer name: Nicolas Lapalu

Reviewer Comments to Author:

This manuscript describes the adaptation of the Colletotrichum genus to monocotyledonous and dicotyledonous plants with regard to the content and expression of genes from 30 genomes, with a sub-sampling of 4 genomes for transcriptomic analyses.
Major remarks:
"Considering that the analyses carried out are affected by the sampling, as closely related species are likely to have more shared genes compared to species that are more distant from others," Yes, Indeed, it's clearly a possible bias due to the sampling, as you write. As you considered all genomes together to define specific genes, monocot specific species have few specific genes due to their phylogenetic proximity. Based on this, could you address these observations based on combination of figure 1 and 2:
1. The number of specific genes in C.eremochloae (1608) vs in C.sublineola (1643), while divergence time between both seems short and similar to the group of C.lupini, C.costaricense (monoct) … with approximatively 100 genes specific to each species. How could such closely related genomes have acquired so many specific genes in such a short time compared with other species during the same period of evolution?
2. Same remark for in C.phormii (911) vs C.salicis (286), when it's even more disturbing with the switch to dicot and a loss of many genes for C.salicis.
For both cases mentionned above, a detailed comparison between the two genomes could be useful to obtain some explanations of the events and genes involved.
Moreover, interpretation of the phylogenetic tree (Figure 1), could be lead to propose three clusters of genomes, based on evolution time and plant host: Monocot, Dicot "old" (C.orbiculare, C.noveboracense, …) and Dicot "young" (C.melonis, C.cuscutae, …). Did the authors attempt an analysis with a such view of the data? Maybe that will complete the view of C.acutatum complex (46 genes) vs C.graminciola complex (28 genes) form which C.orchidophylum and C.phormis are excluded.
Finally, one of the most interesting thing is the proximity of C. phormii and C.salicis in the same clade but with a recent host specialization. Despite the poor quality of the genome of C.salicis vs C.phormii, an comparative genomic approach with a tool like Synchro could provide clues as to gene losses and their location (all along the genome/ specific regions).
Figure 3: Please explain further Figure 3 A, described as a PCA. No axis (dimension) has been shown with a % explaining the divergence between organisms. This is confusing and does not allow me to know whether the gene sets used to compare the 4 genomes are only shared genes or all genes. The rest of the figure is much clearer and the comments are clear on the response to species specificity (under/over expression of genes) for each genome.

Figure 4: "the expression of the orthologous genes was clustered for the four fungal species (Figure 4A)" As written, it is assumed that you used ortholog genes established between the 4 species, this does not appear to be the case with so many genes missing in C.graminicola in figure 4. To continue on this point, I have not found the minimum number of species found in a cluster to set a cluster of orthologs (maybe written but not found). What is the threshold for divergence or sequence similarity? Have you considered sequence length (query coverage vs subject coverage) to allow clustering of potentially split/fragmented genes in annotations?
Minor remarks:
The authors limit their analysis to 30 genomes, whereas more than 270 genomes of Colletotrichum are available, from over 70 species. Research time is clearly longer than the time to generate genomic resources, but it could be interesting to list a few new genomes missing from those analysed and that could have significant added value (particularly if sequenced in long reads, providing complete genomes).
Transcriptomic analyses were carried out on 4 genomes. The choice of the genomes was not discussed, and maybe done by convenience with strains available at the lab. In fact, C.higginsianum is well sequenced, assembled and studied and chosen as one of the specific hosts of dicotyledons, whereas it is a member of the C.destructivum complex. Similarly, C.phormii appears to be a recent species with an adaptation to monocots.
L 113 : "species with bigger genomes are characterized by a lower GC content", please rewrite the link between genome size and GC content. Between species of same genus genome size is most often linked to the invasion of TE element (RIPed or not in fungi). Strongly ripped genomes (Leptosphaeria, Venturia) are not always large compared to the size of other species.
Data availability: All genomes were released in public Databases. I do not find accession numbers for RNA-Seq runs.
Many supplementary details have been provided. I appreciate the BUSCO logs for checking the completeness of gene sets, which provide me some clues about the quality of genome annotation, that was never discussed or pointed out in the manuscript as possible source of bias.
Overall, the manuscript is very interesting and confirms the results previously identified in terms of specificities of CAZy families associated with host plant adaptation in the Colletotrichum genus. The authors demonstrate a great knowledge of the CAZome and associated biological processes, which provides a great deal of valuable information for the community working on Colletotrichum and more generally for all those working on such enzymes. Finally, the transcriptomic data suggest that species specificity and host adaptation are more related to an expression pattern than to specific gene content, than a specific gene content.

Methods

Are the methods appropriate to the aims of the study, are they well described, and are necessary controls included? Choose an item.

Conclusions

Are the conclusions adequately supported by the data shown? Choose an item.

Reporting Standards

Does the manuscript adhere to the journal's guidelines on minimum standards of reporting? Choose an item.

Choose an item.

Statistics

Are you able to assess all statistics in the manuscript, including the appropriateness of statistical tests used? Choose an item.

Quality of Written English

Please indicate the quality of language in the manuscript: Choose an item.

Declaration of Competing Interests

Please complete a declaration of competing interests, considering the following questions:

- Have you in the past five years received reimbursements, fees, funding, or salary from an organisation that may in any way gain or lose financially from the publication of this manuscript, either now or in the future?
- Do you hold any stocks or shares in an organisation that may in any way gain or lose financially from the publication of this manuscript, either now or in the future?
- Do you hold or are you currently applying for any patents relating to the content of the manuscript?
- Have you received reimbursements, fees, funding, or salary from an organization that holds or has applied for patents relating to the content of the manuscript?
- Do you have any other financial competing interests?
- Do you have any non-financial competing interests in relation to this paper?

If you can answer no to all of the above, write 'I declare that I have no competing interests' below. If your reply is yes to any, please give details below.

I declare that I have no competing interests

I agree to the open peer review policy of the journal. I understand that my name will be included on my report to the authors and, if the manuscript is accepted for publication, my named report including any attachments I upload will be posted on the website along with the authors' responses. I agree for my report to be made available under an Open Access Creative Commons CC-BY license (http://creativecommons.org/licenses/by/4.0/). I understand that any comments which I do not wish to

be included in my named report can be included as confidential comments to the editors, which will not be published.

Choose an item.

To further support our reviewers, we have joined with Publons, where you can gain additional credit to further highlight your hard work (see: https://publons.com/journal/530/gigascience). On publication of this paper, your review will be automatically added to Publons, you can then choose whether or not to claim your Publons credit. I understand this statement.

Yes Choose an item.
